# Wonky whales: the evolution of cranial asymmetry in cetaceans

**DOI:** 10.1186/s12915-020-00805-4

**Published:** 2020-07-10

**Authors:** Ellen J. Coombs, Julien Clavel, Travis Park, Morgan Churchill, Anjali Goswami

**Affiliations:** 1 Genetics, Evolution, and Environment Department, University College London, Gower Street, London, WC1E 6BT UK; 2 Department of Life Sciences, Natural History Museum, London, Cromwell Road, London, SW7 5BD UK; 3 Univ Lyon, Université Claude Bernard Lyon 1, CNRS, ENTPE, UMR 5023 LEHNA, F-69622, Villeurbanne, France; 4 Department of Earth Sciences, University of Oxford, Oxford, OX1 3AN UK; 5 Department of Biology, University of Wisconsin-Oshkosh, Oshkosh, WI 54901 USA; 6 Department of Earth Sciences, University College London, Gower Street, London, WC1E 6BT UK

**Keywords:** Trait evolution, Asymmetry, Cetaceans, Morphometrics, Macroevolution

## Abstract

**Background:**

Unlike most mammals, toothed whale (Odontoceti) skulls lack symmetry in the nasal and facial (nasofacial) region. This asymmetry is hypothesised to relate to echolocation, which may have evolved in the earliest diverging odontocetes. Early cetaceans (whales, dolphins, and porpoises) such as archaeocetes, namely the protocetids and basilosaurids, have asymmetric rostra, but it is unclear when nasofacial asymmetry evolved during the transition from archaeocetes to modern whales. We used three-dimensional geometric morphometrics and phylogenetic comparative methods to reconstruct the evolution of asymmetry in the skulls of 162 living and extinct cetaceans over 50 million years.

**Results:**

In archaeocetes, we found asymmetry is prevalent in the rostrum and also in the squamosal, jugal, and orbit, possibly reflecting preservational deformation. Asymmetry in odontocetes is predominant in the nasofacial region. Mysticetes (baleen whales) show symmetry similar to terrestrial artiodactyls such as bovines. The first significant shift in asymmetry occurred in the stem odontocete family Xenorophidae during the Early Oligocene. Further increases in asymmetry occur in the physeteroids in the Late Oligocene, Squalodelphinidae and Platanistidae in the Late Oligocene/Early Miocene, and in the Monodontidae in the Late Miocene/Early Pliocene. Additional episodes of rapid change in odontocete skull asymmetry were found in the Mid-Late Oligocene, a period of rapid evolution and diversification. No high-probability increases or jumps in asymmetry were found in mysticetes or archaeocetes. Unexpectedly, no increases in asymmetry were recovered within the highly asymmetric ziphiids, which may result from the extreme, asymmetric shape of premaxillary crests in these taxa not being captured by landmarks alone.

**Conclusions:**

Early ancestors of living whales had little cranial asymmetry and likely were not able to echolocate. Archaeocetes display high levels of asymmetry in the rostrum, potentially related to directional hearing, which is lost in early neocetes—the taxon including the most recent common ancestor of living cetaceans. Nasofacial asymmetry becomes a significant feature of Odontoceti skulls in the Early Oligocene, reaching its highest levels in extant taxa. Separate evolutionary regimes are reconstructed for odontocetes living in acoustically complex environments, suggesting that these niches impose strong selective pressure on echolocation ability and thus increased cranial asymmetry.

## Background

Cetaceans (whales, dolphins, and porpoises) are the most taxonomically diverse aquatic mammal clade [1] and inhabit most major ocean basins and some rivers [2]. Whales appear in the fossil record approximately 52.5 million years ago (Mya), with the two extant cetacean suborders, mysticetes (baleen whales) and odontocetes (toothed whales), diverging around 39 million years ago (Mya) [3]. Mysticetes evolved large body sizes and specialisations for bulk filter feeding whilst odontocetes evolved echolocation (biosonar) [4–6] and employ various raptorial and/or suction feeding strategies [7, 8]. Cetaceans have undergone extensive morphological changes to adapt to a fully aquatic lifestyle [9, 10] and show extremely divergent morphologies compared to their terrestrial artiodactyl relatives. Some of the most striking changes have occurred in the skull, including the posterior displacement of the nares, maxilla and premaxilla, and a shortening of the nasals [11–13].

Odontocetes are well-known to have asymmetrical crania [14], whereas mysticetes have bilaterally symmetrical skulls and no asymmetry in the nasofacial region [15]. Fahlke et al. [16] hypothesised that basilosaurids and protocetids (early cetaceans belonging to the archaeocetes) also have cranial asymmetry thought to be linked to aquatic directional hearing with the most conspicuous asymmetry occurring in the rostrum [15, 16]. Asymmetry in odontocetes is always unidirectional, with a posterior and sinistral shift in the bones, linked to the hypertrophied melon, phonic lips, and nasal sacs, all of which are associated with high-frequency sound production and echolocation [16, 17]. Most of this asymmetry appears in the dorsal opening of the nares [14, 15, 18] and appears to be correlated with the degree of elevation in the cranial vertex [11]. Species with high cranial vertices such as physeterids, kogiids, and ziphiids tend to have the most asymmetrical crania, likely because a functional component of asymmetry pertains to soft facial anatomy and consequently drives evolution of the underlying bony structures [11].

Odontocete asymmetry is thought to have evolved as a result of an evolutionary hyperallometric investment into sound-producing structures to facilitate the production of high frequency vocalisations [11, 19–22], but alternative explanations have been put forward. MacLeod et al. [18] proposed that skull asymmetry is a by-product of the selection pressure for an asymmetrically positioned larynx, an aquatic adaptation which enables the swallowing of large prey underwater without mastication. However, this has been argued against because reduction of tooth size and loss of shearing occlusion started after asymmetry was well developed, suggesting that swallowing prey whole may not be the driver of asymmetry [16]. Alternatively, cranial asymmetry in basilosaurids and protocetids is thought to be linked to aquatic directional hearing [16]. The limited or lack of asymmetry in mysticetes, which do not echolocate and instead specialise in low and infrasonic frequencies [23–25], suggests directional cranial asymmetry is more likely related to echolocation than hearing [15].

Previous studies have focused on either odontocete cranial shape and function [13], archaeocete asymmetry [16], or mysticete symmetry with modern odontocetes and archaeocetes for comparison [15]. There is, however, little resolution on how cranial asymmetry evolved during the transition from archaeocetes to modern whales (Neoceti) [16], and little is known about archaeocete asymmetry and its relationship, if any, to that of odontocetes [26]. To assess when and how often asymmetry may have arisen, where and if it is present in the archaeocete skull, and how it relates to the evolution of echolocation, it is necessary to adopt a comparative approach by broadly sampling across living and extinct cetaceans. Here we use geometric morphometric techniques to quantify asymmetry in the skull across modern and fossil species of Cetacea. We then use these data to reconstruct the evolution of asymmetry across cetaceans and test for shifts and jumps in the rate of evolution of cranial asymmetry across the cetacean phylogeny. Finally, we use these results to test potential factors associated with the evolution of asymmetry in specific cetacean clades, including presence or absence of echolocation, echolocation frequency, and inhabiting acoustically complex or high-pressure environments such as shallow rivers, cluttered icy waters, and deep ocean.

## Results

### Cranial asymmetry across cetaceans

Comparing the sum radii (Σ*ρ*_spec_) for each specimen in our data set, we found that odontocetes, especially the monodontids, physeterids, and kogiids, are the most asymmetrical of the cetaceans (Table 1).

**Table 1:** List of cetacean specimens with the highest sum radii across the cranium (Σ*ρ*_spec_)

	Species	Family	Suborder	Sum radii (Σ*ρ*_spec_)
1	*Monodon monoceros* USNM 267959	Monodontidae	Odontocete	0.546
2	*Orycterocetus crocodilinus* USNM 22926	Physeteridae	Odontocete	0.518
3	*Aulophyseter morricei* UCMP 81661	Physeteridae	Odontocete	0.489
4	*Kogia breviceps* USNM 22015	Kogiidae	Odontocete	0.462
5	*Kogia simus* NHMUK 1952.8.28.1	Kogiidae	Odontocete	0.457
6	*Physeter macrocephalus* NHMUK 2007.1	Physeteridae	Odontocete	0.456
7	*Delphinapterus leucas* USNM 305071	Monodontidae	Odontocete	0.453
8	*Platanista gangetica* USNM 172409	Platanistidae	Odontocete	0.449
9	*Globicephala melas* NMNZ MM001946	Delphinidae	Odontocete	0.410
10	*Pseudorca crassidens* USNM 11320	Delphinidae	Odontocete	0.408

*ρ* is the radius value calculated as the Euclidean distance between the computer -mirrored landmark and the manually placed landmark. The larger the value for *ρ*, the longer the radii for a corresponding landmark and the more it is displaced, indicating asymmetry between the two sides of the cranium

**Fig. 1:**
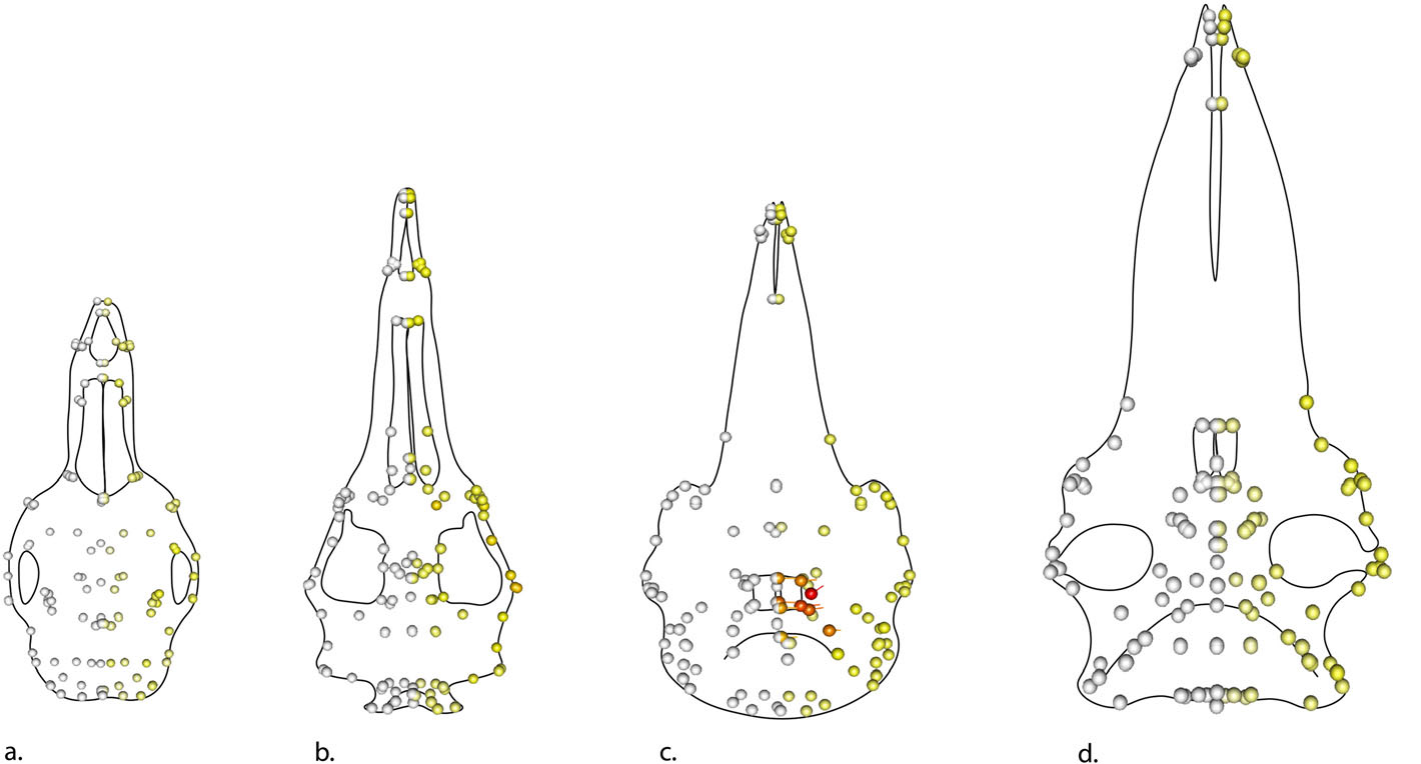
Average radii per landmark (x̄*ρ*_land_) for each taxon group. Landmarks superimposed onto a stylised skull which represents an average specimen for that group. Cooler yellows show less asymmetry, warmer oranges and reds show more asymmetry. The white landmarks are fixed reference landmarks (1-66) and therefore show no movement. From left to right: **a** the average landmark radii (x̄*ρ*_land_) for terrestrial artiodactyls, **b** the average landmark radii for archaeocetes, **c** the average landmark radii for odontocetes, and **d** the average landmark radii for mysticetes. Landmarks on skulls **a** and **d** consist of pale yellows indicating low asymmetry. The landmarks on skull **b** are pale yellow, with darker yellows on the jugal, orbit, and rostrum indicating a higher level of asymmetry. Oranges and red landmarks in the nasal, posterior premaxilla, and posterior maxilla on skull **c** (the odontocete) indicate a high level of asymmetry. Skulls not to scale

Of 172 specimens (162 cetaceans + 10 terrestrial artiodactyls for reference), the top 43 with the highest sum radii per specimen (Σ*ρ*_spec_ ) are odontocetes. The highest ranking mysticetes are a balaenopterid (MNNZ MM001630) (Σ*ρ*_spec_ = 0.300), *Aglaocetus moreni* (Σ*ρ*_spec_ = 0.298), and *Janjucetus hunderi* (Σ*ρ*_spec_ = 0.295), ranked 47th, 50th, and 51st, respectively (Additional file 1: Table S1). The highest ranking archaeocetes are *Basilosaurus isis* (Σ*ρ*_spec_ = 0.308) and *Zygorhiza kochii* (Σ*ρ*_spec_ = 0.306) ranked 44th and 45th, respectively. The highest-ranking terrestrial artiodactyls do not appear until the 129th (*Capricornis sumatrensis,* Σ*ρ*_spec_ = 0.205) and 139th (*Bos* sp.*,* Σ*ρ*_spec_ = 0.195) positions*.* The mysticetes and terrestrial artiodactyls dominate the lower end of the ranking with eight of the last ten positions occupied by extant balaenids and balaenopterids and one fossil pelocetid (Additional file 1: Table S1). For the whole cetacean data set, the most asymmetric landmarks are the nasals, the maxilla at the sutures with the nasals and premaxilla, and the posterior, dorsal premaxilla (Table 2). This distribution is heavily influenced by the odontocete sample (*n* = 120, 74% of cetacean specimens). For this reason, each cetacean suborder and the terrestrial artiodactyls were analysed separately. The mean total cranial radii for odontocetes is the highest of all groups at x̄*ρ* = 0.290 (Table 2). The most asymmetric landmarks for odontocetes are the dorsal maxilla (suture with nasal and premaxilla), nasals, and the posterior-dorsal maxilla (Table 2). Terrestrial artiodactyls have the lowest average total radii value (asymmetry) across the skull (x̄*ρ* = 0.171), followed closely by mysticetes (x̄*ρ* = 0.191). Archaeocetes showed a moderately high level of asymmetry in the skull (x̄*ρ* = 0.251). Cetacean subgroups differ greatly in identity of the most asymmetric landmarks and magnitude of landmark asymmetry (Table 2). For example, the most asymmetric landmark in odontocetes is the dorsal medial maxilla (suture with nasal and premaxilla) with the average sum of radii for that landmark (x̄*ρ*_land_) = 0.013, whereas the most asymmetric landmark in mysticetes is the posterior ventral lateral most point of the maxilla, with x̄*ρ*_land_ = 0.005 (Table 2). This difference is evident when comparing average landmark asymmetry across the groups (Fig. 1).

**Table 2:** List of the five landmarks with the greatest variation across the cranium for all cetaceans, archaeocetes, odontocetes, mysticetes, and terrestrial artiodactyls

Suborder	Average asymmetry in the skull (x̄*ρ*)	1st highest landmark of variation		2nd highest landmark of variation		3rd highest landmark of variation		4th highest landmark of variation		5th highest landmark of variation		Specimen showing top 5 landmarks (red)
		Landmark description	x̄*ρ*_land_	Landmark description	x̄*ρ*_land_	Landmark description	x̄*ρ*_land_	Landmark description	x̄*ρ*_land_	Landmark description	x̄*ρ*_land_	
**All cetaceans**	0.268	**L74**: Dorsal medial maxilla (suture with nasal and premaxilla)	0.010	**L68**: Posterior lateral corner of nasal	0.009	**L71**: Posterior dorsal premaxilla	0.009	**L121**: Posterior point of nasal	0.008	**L67**: Left anterior lateral nasal	0.007	
**Archaeocetes**	0.251	**L72:** Anterior lateral ventral premaxilla	0.007	**L69**: Tip of rostrum, anterior dorsal side, anterior midline of tooth row	0.007	**L70**: Anterior dorsal premaxilla	0.007	**L82:** Jugal posterior ventral	0.006	**L73:** Anterior lateral ventral maxilla	0.006	
**Odontocetes**	0.290	**L74:** Dorsal medial maxilla (suture with nasal and premaxilla)	0.013	**L68:** Posterior lateral corner of nasal	0.011	**L71:** Posterior dorsal premaxilla	0.010	**L121:** Posterior point of nasal	0.009	**L67:** Left anterior lateral nasal	0.009	
**Mysticetes**	0.191	**L78:** Posterior ventral lateral most point of maxilla (near orbit)	0.005	**L79:** Posterior tooth row lateral maxilla	0.005	**L72:** Anterior lateral ventral premaxilla	0.005	**L71:** Posterior dorsal premaxilla	0.005	**L122:** Anterior medial frontal	0.005	
**Terrestrial artiodactyls**	0.171	**L83:** Lateral posterior frontal (posterior lateral parietal suture)	0.006	**L87:** Anterior medial parietal	0.005	**L97:** Posterior lateral most point of the mandibular articular process	0.005	**L80:** Jugal anterior dorsal	0.005	**L98:** Lateral posterior squamosal (occipital suture)	0.004	

*ρ* is the radius value calculated as the Euclidean distance between the computer-mirrored landmark and the manually placed landmark. The larger the value for *ρ*, the more it is displaced, indicating asymmetry between the two sides of the cranium. x̄*ρ* is the average of the total radii (∑*ρ*) values across the skull for all the specimens in that group. x̄*ρ*_land_ is the average radii across all specimens in that group for that landmark. Each image shows the position of the five landmarks of greatest variation for each respective group. Skulls not to scale

The basilosaurid and protocetid archaeocetes show a high level of asymmetry, akin to the levels seen in fossil and extant odontocetes (Additional file 1: Table S1). The contribution of rostral landmarks to overall cranial asymmetry in these archaeocete families ranges from 13.8% in *Aegyptocetus tarfa* to 31.3% in *Artiocetus clavis.* The average amount of asymmetry concentrated in the rostrum is higher in archaeocetes (19.3%) (this includes the families Kekenodontidae, Pakicetidae, and Remingtonocetidae, which are not commonly associated with asymmetry) than in mysticetes (14.2%) and odontocetes (14.7%) (Additional file 1: Table S2–4).

**Fig. 2:**
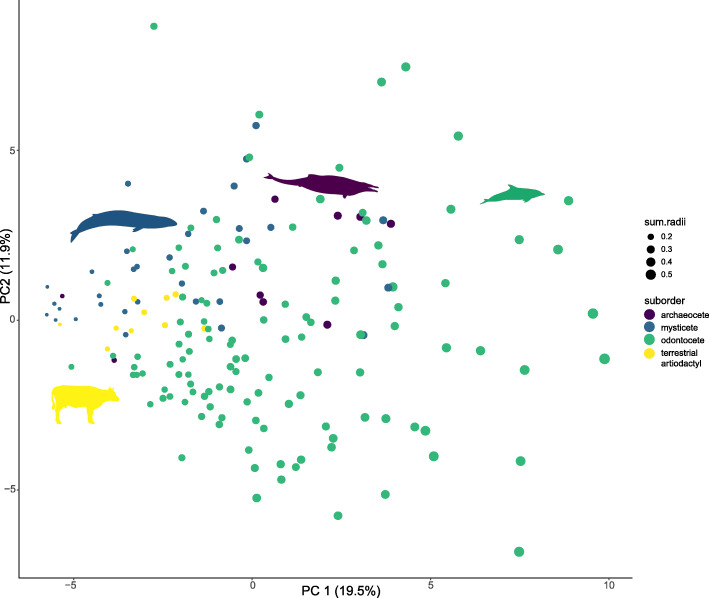
Principal components 1 and 2 for full data set (*n* = 172, including 10 terrestrial artiodactyls). Circle size size reflects the sum radii in the skull for each specimen (∑*p*_spec_), with larger circles indicating higher ∑*p*_spec_. A morphospace labelled with a specimen key is provided in the Additional file 1: Fig. S5—principal components plot with PC1 and PC2 plotted for each specimen. Silhouettes are from Phylopic with credit attributed to Chris Huh and used under the Creative Commons Licence [30]

As deformation during fossil preservation may create nonbiological asymmetry and previous studies suggest this may be concentrated in the rostrum of some fossil cetaceans [15] (see Martínez-Cáceres et al., [27] and Martínez-Cáceres and de Muizon [28]), we also ran analyses without any fossils and without rostral landmarks. When fossils were removed, there was a decrease in asymmetry in mysticetes (x̄*ρ* = 0.142) (extant mysticetes: *n* = 12, 7% of the data set). In contrast, fossil odontocetes are more symmetrical than most extant odontocetes (Additional file 1: Table S1). For this reason, when fossil odontocetes were removed, the level of average asymmetry in the odontocete skull increased marginally (x̄*ρ* = 0.292) (extant odontocetes: *n* = 72, 44% of the data set). Excluding rostral landmarks had the most impact on archaeocetes and mysticetes, as some of the highest levels of asymmetry in those clades are found in the rostral region (Table 2; Additional file 1: Tables S2–4). However, overall, removal of the rostral landmarks had only a minor effect on results (Additional file 1: Fig. S1-S3 [29], Table S5b). Principal component analysis of landmark asymmetries showed that odontocetes exhibit a wide range of cranial asymmetry (Fig. 2 [30]) and cranial shape (Additional file 1: Fig. S4). Mysticetes and terrestrial artiodactyls overlap in asymmetry morphospace, whilst archaeocetes have a higher level of asymmetry, similar to more moderately asymmetric odontocetes (Fig. 2). See Additional file 1: Fig. S5 for identification of each specimen in the morphospace.

**Fig. 3:**
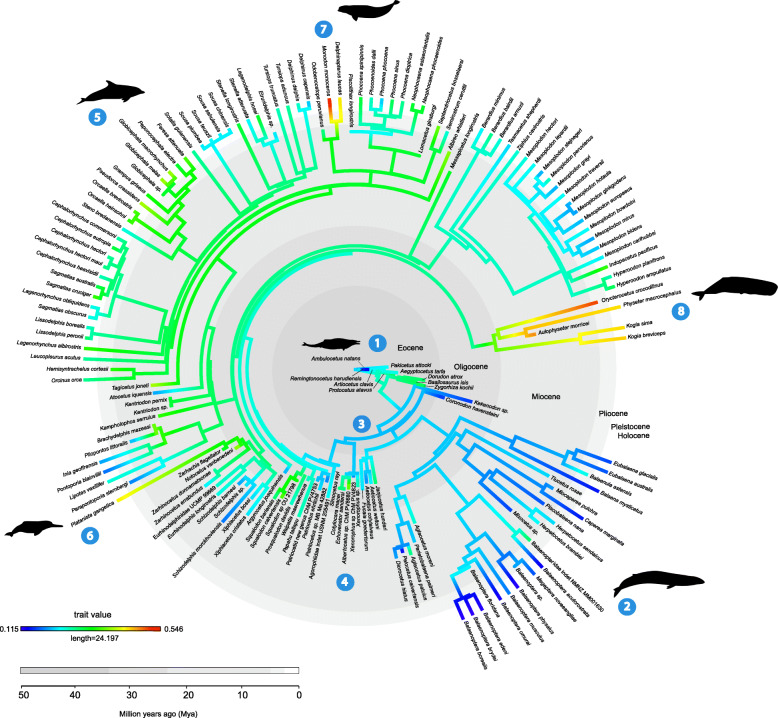
Time-calibrated phylogeny for sampled cetacean species indicating magnitude of cranial asymmetry (∑*p*_spec_). The labels highlight the following points: (1) archaeocetes, (2) mysticetes, (3) the origin of Neoceti (~ 39 Mya) [26], (4) early odontocetes including the xenorophids, (5) odontocetes, (6) the highly asymmetrical *Platantista gangetica*, (7) the highly asymmetrical monodontids, and (8) the highly asymmetrical Physeteroidea. The full data set (*n* = 162) is used. Phylogeny based on Lloyd and Slater [29]. Silhouettes are from Phylopic with credit attributed to Chris Huh and used under the Creative Commons Licence [30]

The basilosaurid and protocetid archaeocetes show a high amount of asymmetry (∑*p*_spec_) in the cranium (Fig. 3 (1)), similar to that observed in early odontocetes, such as xenorophids (Fig. 3 (4)). Asymmetry decreases towards the base of Neoceti, and mysticetes show the lowest level of cetacean asymmetry observed in the entire data set (Fig. 3 (2)), overlapping with terrestrial artiodactyls (Fig. 2). As expected, odontocetes show higher values of asymmetry but span nearly the full range of asymmetry morphospace (Fig. 2). The highest asymmetry is seen in the platanistids, monodontids, and physeteroids (Fig. 3 (6–8)). There are some exceptions within odontocetes, however, such as lower levels of asymmetry in the other extant river dolphins (*Inia*, *Pontoporia*, and *Lipotes*) [21, 31]. Lower asymmetry is also observed in the family Eurhinodelphinidae [32], the extant phocoenids [26, 33], and genus *Sousa* [14] (Fig. 3 (5)).

### Evolutionary models of asymmetry

**Fig. 4:**
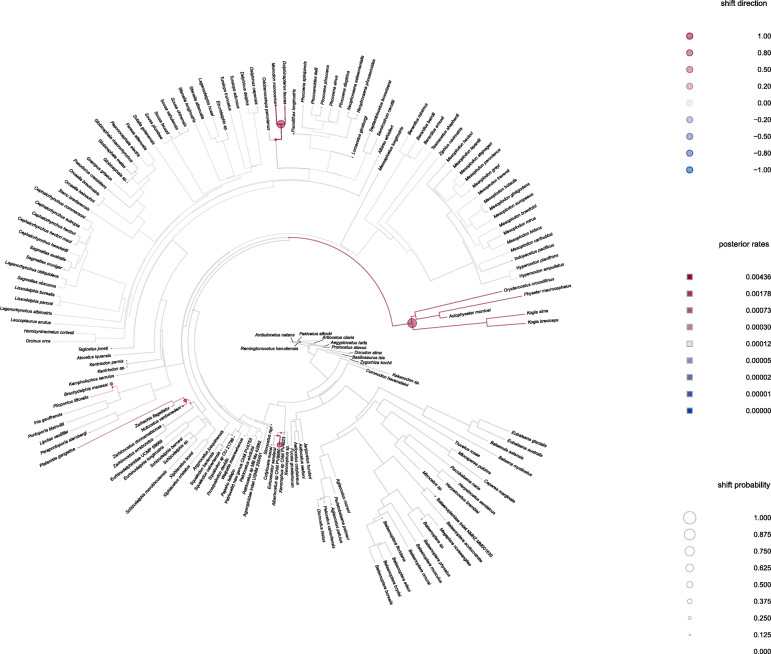
Reconstructed probability of shifts in cetacean cranial asymmetry. Reconstructed probability along each branch of the phylogeny under the assumption of relaxed Brownian motion with a Half-Cauchy distribution for the prior density of the rate scalar. Circles indicate a shift in the trait on either the branch or in the whole clade. The colour of the circle indicates the shift direction with red indicating forward shifts and blue indicating backwards shifts. The size of the circle indicates the probability of the shift occurring in that position in the clade with the largest circle (here, 0.750) indicating the highest probability of a shift occurring. The colour of the branch itself indicates posterior rates for that branch with red showing higher, increasing rates and blue showing lower, decreasing rates. The background rate is shown as grey. The asymmetry value is given as the sum of radii per specimen (∑*p*_spec_). A trace of the chain is provided in Additional file 1: Fig. S10—Gelman diagnostics for the two chains. Phylogeny based on Lloyd and Slater [29]

Reconstructing shifts in the rate of asymmetry evolution supported three shifts with a probability > 0.375, with several additional shifts at lower probabilities (Fig. 4). There is a probability (0.375) of a shift in asymmetry occurring in the family Xenorophidae during the Early Oligocene (~ 30 Mya); this represents the first, large shift in asymmetry in the cetacean phylogeny (Fig. 4). Another shift occurs in the physeteroids (~ 23 Mya; probability = 0.750), and a later shift (probability = 0.625) is seen in the Late Miocene/Early Pliocene in the Monodontidae (Fig. 4). There are two smaller shifts (probability = 0.250) in the Squalodelphinidae and Platanistidae in the Late Oligocene/Early Miocene and later in the ‘inioids’. There are no high probability shifts in asymmetry in the mysticete suborder, nor in the archaeocetes. Shifts with small (< 0.250) probabilities of occurrence are scattered throughout the phylogeny (Fig. 4), including one low probability shift at the root of Archaeoceti, but most shifts occur within the odontocetes. There is no measurable probability of a shift occurring in the archaeocete protocetids and basilosaurids. A slower or decreasing rate of asymmetry evolution is reconstructed within Mysticeti. Surprisingly, no shifts are reconstructed in the ziphiids, an odontocete family with bizarre asymmetrical premaxillary crests in most species (e.g. *Ziphius cavirostris*).

**Fig. 5:**
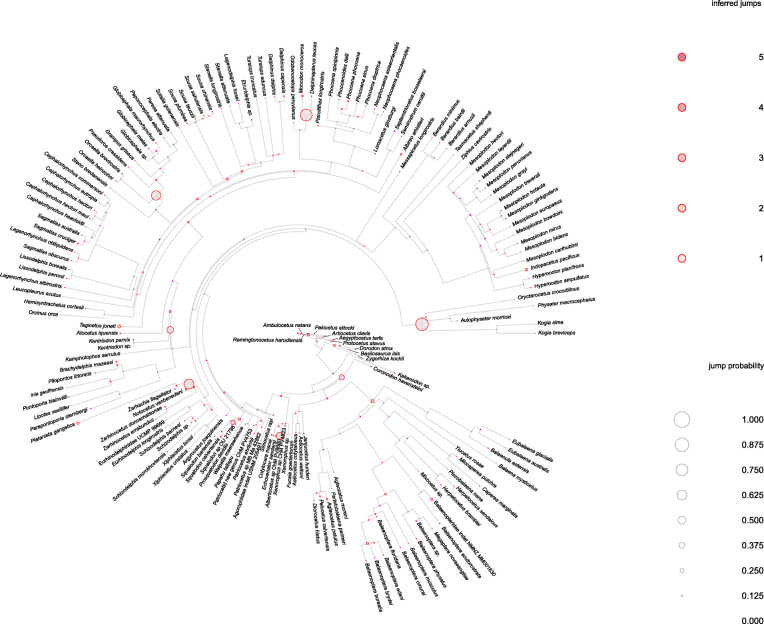
Reconstructed probability of jumps in the rate of cetacean cranial asymmetry. The model also predicts the *number* of jumps which may have occurred. The size of the circle indicates the probability of the jump occurring in that position in the clade with the largest circle (here, 0.750) indicating the highest probability of a jump occurring. The colour of the circle indicates the number of inferred jumps, where dark red = 5 and pale red = 1. The asymmetry value is given as the sum of radii per specimen (∑*p*_spec_). A trace of the chain is provided in Additional file 1: Fig. S10—Gelman diagnostics for the two chains. Phylogeny based on Lloyd and Slater [29]

We found a similar pattern for ‘jumps’ (a temporary or rapid change in the trait) (Fig. 5) as we did for ‘shifts’, with the addition of several jumps occurring in the Mid-Late Oligocene. The largest jumps (probability = 0.750) occur in the physeteroids and the monodontids. Smaller jump probabilities (0.625) occur in the Delphinidae, specifically in the subfamily Globicephalinae (e.g. *Globicephala* spp., *Pseudorca crassidens*) and Platanistidae and Squalodelphinidae and also at around probability = 0.40 in the xenorophids and the kentriodontids (Fig. 5). The traces of the chains for the two models (shifts and jumps) show that a successful burn-in occurs before 25% of the model iterations are run, justifying the use of the default value (Additional file 1: Fig. S6, S7 and Model diagnostics [34–36]). All model diagnostics are provided in Additional file 1: Fig. S6–10; Table S6 and Model diagnostics section [34–36].

### Evolutionary models of influence on asymmetry

Inclusion or exclusion of the rostrum made no difference to the ordering of the ‘goodness of fit’ of the models (Table 3; Additional file 1: Table S5b [29]). There was no difference in the ordering of the ‘goodness of fit’ of the top models when we ran all models with a phylogeny that includes only species that appear in a character matrix from Lloyd and Slater [29] (Additional file 1: Fig. S11-S13 Table S5c [29]). For this reason, the results focus on the analyses which include the rostral landmarks and the original ‘genus tree’ phylogeny.

**Table 3:** Five best-fit evolutionary models for cranial asymmetry ranked according to the Akaike Information Criteria (AIC)

Model	Full landmark data set		No rostrum	
	Rank	Akaike Information Criteria (AIC)	Rank	Akaike Information Criteria (AIC)
1. ‘OUM-regime’	1	− 448	1	− 498
2. ‘OUM-regime-split’	2	− 445	2	− 496
3. ‘OUM-echo-freq’	3	− 403	3	− 449
4. ‘OUM-ancestral’	4	− 379	4	− 424
5. ‘OUM-echo’	5	− 373	5	− 422

Models are detailed in Table 4—models testing whether changes in cetacean cranial asymmetry are associated with other discrete traits

The best fit model for both data sets is the ‘OUM-regime’ (AIC = − 448) (Table 3), which is the model with a selective regime suggesting that the monodontids, physeteroids, and platanistids are evolving under a single different regime (Table 3; Additional file 1: Table S5a [29]), under the assumption of Ornstein–Uhlenbeck (OU). The ‘OUM-regime-split’ model (the ‘regime’ model split into 4 separate ‘regimes’ i.e. one regime (evolutionary state) for the monodontids, one for physeteroids, and one platanistids) also received strong support (AIC = − 445). In both the ‘OUM-regime’ and ‘OUM-regime-split’ models, archaeocetes are placed into one regime, mysticetes in another, and the remaining odontocetes in a third. The third best fit model is the ‘OUM-echo-freq’ model (AIC = − 403) (Table 3), again under an OU assumption, with species categorised by their predominant echolocation/sound group.

Phylogenetic ANOVAs supported the ‘OUM-regime’, ‘OUM-regime-split’, and ‘OUM-echo-freq’ models as factors significantly associated with total cranial asymmetry (Σ*ρ*_spec_) across cetaceans (*F* = 26.97, *p* < 0.001; *F* = 15.78, *p* < 0.001; *F* = 5.83, *p* < 0.001, respectively). Geological age, suborder, and presence/absence of echolocation were not significantly associated with cranial asymmetry (*F* = 1.10, *p* = 0.36; *F* = 1.57, *p* = 0.21; *F* = 1.44, *p* = 0.23, respectively). After correction for false discovery rate (using the Benjamini-Hochberg method [55, 56]), the regime, regime-split, and echolocation frequency models remained significant (*p* < 0.001, *p* < 0.01, *p* < 0.001, respectively) (Additional file 1: Table S7 [55, 56]). Hereafter, results with the Benjamini-Hochberg correction are discussed.

## Discussion

Our analyses of cranial asymmetry through the evolutionary history of whales suggests that the common ancestor of living whales (mysticetes and odontocetes) did not possess an asymmetric cranium, and thus, it is unlikely that echolocation was present at that stage of whale evolution or at any point in mysticete evolution. Cranial asymmetry is highest in crown odontocetes and first becomes a major feature of odontocete crania in the Early Oligocene soon after their divergence from mysticetes. This period has previously been identified as one of unusually high diversity and evolution in neocete skull morphology [13, 37, 57] alongside an explosive and rapid radiation of crown cetaceans [38, 57, 58].

Rostral asymmetry is observed in some archaeocetes and is potentially related to directional hearing, possibly increased by deformation in some cases. Fahlke et al. [16] suggest that *Artiocetus clavis* (GSP-UM 3458—the same specimen as used in this study) was found palate-up with no evident compression or deformation and further suggest that archaeocete asymmetry in the rostrum is consistent in direction. We found this same rostral asymmetry in this and other archaeocetes along with asymmetry in the jugal, orbit, and squamosal. This rostral asymmetry disappears in Neoceti and later arises in the nasofacial region in odontocetes. In archaeocetes, four of the ten most asymmetric landmarks (∑*p*_land_) were located in the rostrum (Table 2). This distribution could be inferred as torsion in the archaeocete rostrum as part of a complex of traits which led to directional hearing [16]. This asymmetry then disappears during the transition from archaeocetes to early neocetes (Fig. 3; Additional file 1: Table S1). It is unclear whether this is due to an actual shift from a primitive form of aquatic directional hearing in specific archaeocetes (the basilosaurids and protocetids, as suggested by Fahlke et al. [16]) to a different regime (i.e. to high-frequency sound production in the odontocetes and low-frequency hearing in the mysticetes), or whether this is simply asymmetry unrelated to function. Asymmetry unrelated to function is reported for other mammals (e.g. dextral twist in the rostral region of some dogs [59]) or even brought on by developmental and environmental stressors [60, 61]. Further, it could be related to specific feeding strategies such as bottom-feeding or other lateralized behaviours. When looking at the primary landmarks displaying asymmetry in the basilosaurids and protocetids, there is no indication that these are dominated by rostral torsion more than in the other archaeocetes (Additional file 1: Table S2), and instead, asymmetry appears to be spread in no particular pattern across the jugal, squamosal (which are possibly more susceptible to deformation), rostrum, and orbit for these families. Rostral asymmetry in the archaeocetes is at least partly caused by fossil distortion in some specimens [27], but perhaps may also be biologically present in more archaecoete families than previously thought.

We found no high probability shifts (Fig. 4) in asymmetry occurring in the protocetids and basilosaurids, despite a rapid change from high asymmetry to a more symmetrical skull in the early mysticetes such as *Coronodon havensteini* (Fig. 3). We did, however, find evidence for small temporary and rapid change (jumps) in asymmetry in the later archaeocetes (Fig. 5). Echolocation, telescoping, and ecological specialisation rapidly evolved shortly after the divergence of Neoceti from Basilosauridae [4, 38], and there may have been a rapid regime change from directional hearing occurring at the same time, possibly with associated asymmetry.

Asymmetry is lowest in basal mysticetes such as *Coronodon havensteini* and the aetiocetids and remains low in mysticetes from the Oligocene to present. There are no high probability shifts in asymmetry in the mysticetes. Rather, Mysticeti largely display a slower or decreasing rate of the trait.

There are some increases in asymmetry observed in individual mysticetes, for example in Balaenopteridae indet NMNZ MM00163 and *Aglaocetus moreni* (FMNH P13407), but this likely represents taphonomic distortion in the rostrum rather than biological asymmetry. Balaenopteridae indet NMNZ MM00163 especially has some distortion in the supraoccipital, postorbital process, lateral posterior squamosal, and the parietal which likely account for its high Σ*ρ*_spec._

Quantifying cranial asymmetry in living and extinct mysticetes allows reconsideration of the evolution of echolocation in this clade. The consensus is that cranial asymmetry in whales evolved due to the production of high-frequency vocalisations [19–21]. The consistent level of symmetry in the mysticetes corroborates the hypothesis that mysticetes never evolved sophisticated echolocation [25, 62] and also contradicts the hypothesis that this suborder secondarily lost their echolocation capabilities [63]. Our analysis further suggests that echolocation was likely not present in the common ancestor of mysticetes and odontocetes [25, 62] but evolved early in the common ancestor of odontocetes shortly after they diverged from mysticetes [4]. As reported in Fahlke and Hampe [15], mysticete crania are similar in magnitude of asymmetry to terrestrial artiodactyls (Table 2; Fig. 2). In mysticetes, the highest level of cranial asymmetry was found across the rostrum (anterior and posterior maxilla and premaxilla), likely due to deformation. In some extant specimens, we observed that the tip of the rostrum has dried out and partly split apart. Even with drying-out and potential taphonomic deformation, the levels of asymmetry in mysticetes were lower than asymmetry seen in archaeocetes and much lower than that of odontocetes.

Cranial asymmetry first appears as a significant morphological trait in the Early Oligocene odontocetes Xenorophidae (Fig. 3, Fig. 4), suggesting that biosonar arose early in odontocete evolution. Odontocete asymmetry is overwhelmingly concentrated in the nasals including the posterior suture with the frontal, maxilla, and premaxilla. Most early odontocetes are less asymmetric (Fig. 3) compared to later extinct and modern forms [14], bar a few exceptions. The extant La Plata dolphin (*Pontoporia blainvillei*) is one of few living odontocetes with cranial symmetry but asymmetric nasal sacs [14], and it ranks here as the least asymmetric odontocete (∑*p*_spec_ = 0.179, Additional file 1: Table S1). Other extant odontocetes with low cranial asymmetry include *Sousa*, *Sotalia*, and *Steno* (Fig. 3) which have been suggested to converge in skull morphology with kentriodontids [14], (Fig. 3). Phocoenids also exhibit a low level of cranial asymmetry (Fig. 3) [26]. This low asymmetry is likely tied to their relatively low peak-power biosonar [22, 64]. Further, many descriptions of eurhinodelphinids have suggested that their crania are only slightly asymmetric [32, 65], as is supported here (Fig. 3). Thus, it should be considered that although some later fossil odontocetes had symmetrical skulls, they may have had asymmetrical nasal sacs as is observed in these extant species.

Macroevolutionary reconstruction of shifts and jumps in cranial asymmetry throughout cetacean evolution supported the first major positive shift (probability = 0.375) in asymmetry occurring in xenorophids during the Early Oligocene (~ 30 Mya) (Fig. 3; Fig. 4). This result adds further evidence to the idea that xenorophids and other odontocetes iteratively evolved specialisations for the production of high-frequency sounds necessary for echolocation [4–6, 39]. The distinct cranial morphology (and by inference, distinct soft tissue morphology) found in xenorophids (e.g. a deep rostral basin, a narrow premaxillary fossa, and a postnarial fossa) indicate a form of echolocation unique to the clade which interestingly, as it became more specialised, also became more asymmetrical, highlighting the importance of this trait for echolocation. The position of xenorophids as the earliest diverging clade within Odontoceti demonstrates that echolocation, telescoping, and ecological specialisation rapidly evolved shortly after the extinction of the Basilosauridae [5, 6, 38]. Since then, cranial asymmetry has increased and remained generally high throughout the odontocete lineage (Fig. 3), bar a few exceptions.

Later shifts are observed in the physeteroids in the Late Oligocene (~ 23 Mya) and in the Squalodelphinidae (this increase in asymmetry is also recently mentioned in Bianucci et al. [66]) and Platanistidae in the Late Oligocene/Early Miocene. The latter two families share marked asymmetry in the premaxillae with the right maxilla narrower than the left in dorsal view [67]. Further, asymmetry is recorded in the frontal and maxillary crests of fossil platanistids such as *Zarhachis flagellator* [67] although the supraorbital crests are not as developed as the extreme maxillary crests in the extant *Platanista gangetica* which is one of the most asymmetric of all odontocete skulls [68]. There is also marked skull asymmetry in the distantly related squalodelphinid, *Notocetus vanbenedeni*, which also sits within the superfamily, Platanistoidea [66, 67].

The best model fit, the regime model (*p* < 0.001) (model: 'regime'), assumes there is a distinct evolutionary regime for the most asymmetrical odontocete specimens (physeteroids, platanistids, and monodontids) indicating a single driver for their extreme asymmetry. We hypothesise that this regime may be linked to the pressures which arise from inhabiting acoustically complex environmental niches. The physeteroids were the first of the major odontocete crown lineages to rapidly diverge and are easily recognisable due to a highly asymmetric facial region and supracranial basin [26]. Their large body size and hypertrophied nasal structures produce a low-frequency multi-pulsed sound [45], which facilitates long range detection of prey [22]. This is highly advantageous when searching for patchy prey, especially as the physical properties of the water itself alter sound velocity and potentially constrain sensory morphology [69].

*Platanista gangetica*, the sole modern survivor of Platanistidae sits alone among river dolphins for having a highly asymmetric cranium and echolocating at broadband low frequency (BBLF). The unique, autapomorphic bony maxillary crests of *Platanista* may help achieve a higher directionality than expected for a cetacean that clicks nearly an octave lower than similar sized odontocetes [43], a feature that would be useful in the turbid, cluttered rivers they inhabit. Other species in this highly asymmetric model include both monodontids: belugas (*Delphinapterus leucas*) and the narwhal (*Monodon monoceros*). *Monodon* remains the most asymmetric skull in the sample, even when the rostrum is removed (Σ*ρ*_spec_ = 0.472) which rules out the possibility that the asymmetric tusk and residual teeth may be skewing the overall Σ*ρ*_spec_ (see below for details). Their unique sound repertoire (narrowband structured, NBS) is ideal for projecting and receiving signals in icy, shallow waters, where the animals can detect targets in high levels of ambient noise and backscatter [44] (Additional file 1: Table S8 [24, 40–42, 45–54, 64, 70–72]). Jumps detected in the delphinids all belong to the subfamily Globicephalinae (Fig. 5). In particular, the highly asymmetrical *Globicephala* (Table 1; Additional file 1: Table S1) has evolved a deep-dive pattern to target a deep-water niche occupied by large, calorific, and fast squid, and its acoustic behaviour is more akin to deep divers than to oceanic delphinids [73]. The cochlea of *Globicephala* is also morphologically different to other delphinids [69], which could also represent adaptation to the extreme acoustic environment of the deep ocean. Further, studies suggest that *Pseudorca* (which also has a highly asymmetric cranium (Table 1)) echolocates with different vertical and horizontal plane patterns to other delphinids [74].

Surprisingly, no jumps or shifts are seen in the deep-diving ziphiids (beaked whales), an odontocete family with bizarre asymmetrical premaxillary crests and an asymmetric prenarial basin (Additional file 1: Fig. S14. The asymmetry of the beaked whale skull is marked [20, 75, 76], so much so that the right premaxilla, premaxillary crest, premaxillary sac fossa, and the nasal bone are around 30% larger than those on the left [77]. Previous studies have suggested that the beaked whale genus *Berardius* (the most basal crown genus) shows the least bilateral asymmetry in the skull [78, 79], and we saw a similar result here. We attribute the underrepresentation of asymmetry in the ziphiid skull to the use of landmarks alone. Whilst detecting asymmetry in the *shifting* of the nasal, premaxilla, and maxilla to the left side of the skull, this method underrepresents the degree of asymmetry in the morphology of the bones themselves. The premaxilla is landmarked with points at the posterior dorsal premaxilla and the dorsal medial maxilla (suture with nasal and premaxilla) which accurately captures asymmetry in the positioning of the bone and its attachment but fails to capture the tapering of the highly asymmetric premaxillary crest itself (Additional file 1: Fig. S14). Future studies in this area should be done with curve sliding semi-landmarks and surface patches to more accurately capture the complex morphology [80] of the premaxillary crests and premaxillary sac fossae in ziphiids which are not represented using fixed landmarks alone.

‘Regime-split’ (model: ‘regime-split’) was the second-best model fit which had a significant effect (*p* < 0.01) on asymmetry in the cranium. This model suggests a different evolutionary regime for each of the most asymmetric groups. As above, it could be hypothesised that the highly asymmetric species live in unique, acoustically complex environments all of which have rather extreme specific environmental selection pressures. The reduction in the *p* value after phylogenetic correction for the regime and regime-split models suggests that the factors influencing asymmetry may be shared by closely related taxa.

Frequency of echolocation (model: ‘echo-freq’) also had a significant effect (*p* < 0.001) on the asymmetry in the cranium and was the third best model fit. Echolocation frequency has been widely suggested as a key driver of asymmetry in the cranium [16, 17] and soft tissues [81]. Although not the best model fit, we suggest that this relationship be investigated in more detail, for example with a more detailed analysis of species-specific echolocation frequencies and associated categories across Cetacea [17]. It is important to note that these methods assume a Brownian motion model, which oversimplifies the actual evolutionary model underlying the evolution of asymmetry (shown here to be better described by an OU model).

We found no support for several other potential drivers for observed patterns of cranial asymmetry, independent of phylogeny. There is no significant effect of geologic age of the specimen (e.g. Eocene, Miocene, extant) on sum radii in the skull (*p* = 0.36). This result is likely because, despite odontocete crania becoming more asymmetrical in most extant families, mysticetes do not. There is no significant effect of ‘suborder’ (*p* = 0.21) on the total sum radii across the cranium. This is not surprising as there is generally a clear phylogenetic relatedness in whether a cetacean is symmetric (mysticete) or asymmetric (odontocete). Presence or absence of echolocation (model: ‘echo’) has no significant effect (*p* = 0.23) on the sum of radii in the cranium. Again, this is not surprising as there is a clear phylogenetic relatedness in whether a cetacean can echolocate, i.e. the odontocete suborder, or not echolocate, i.e. the mysticete suborder.

There is a small chance that skulls used in this study may be more asymmetrical, i.e. deformed or distorted, than a standard skull of the species and therefore this is represented in the placed landmarks and the resulting ∑*p*_spec_. Where possible, we chose skulls based on their overall quality and representation of the species. This was not possible for fossils which are often represented by one specimen, but deformed skulls were removed from the study so as not to falsely imply there is biological asymmetry in the skull when there is none. Further, the sex of the specimen may slightly alter the degree of asymmetry in the skull. Female false killer whales, for example, have a slightly more asymmetrical skull than males [82], and this may partially explain why the individual in this study appears to have a higher level of asymmetry than the other delphinids. However, the sex of this specimen (USNM 11320) is listed as unknown. It is important to note that adult male narwhal exhibit an extreme form of asymmetry in the tusk and vestigial teeth [83]. The specimen in this study (USNM 267959) is female and therefore lacks a highly asymmetric tusk, however, the paired tusks embedded in the maxillae may still exhibit asymmetry [83] and may affect the overlying bone structure. This has not skewed the results seen here as the top 6 landmarks of asymmetry in the *Monodon* skull are in the nasals and posterior premaxilla and maxilla (i.e. not the rostrum or anterior maxilla where imbedded tusks reside). Further, no landmarks were placed on tusks or teeth (see the “Methods” section: “Manually placed landmarks (*Fn*)”), which ensures that extreme asymmetry seen in some tusked species for example, *Odobenocetops*, is not captured in this study. The ‘skew’ in *Globicephala* and monodontid skulls has also been attributed to some asymmetry in the attachment of the neck muscles [84]; however, the asymmetry (Σ*ρ*_land_) in eight landmarks associated with the condyle and posterior cranium do not differ between *Globicephala* and the monodontids compared to other closely related species (e.g. *Feresa attenuata* and *Peponocephala electra*).

Lastly, an argument against the hypothesis that echolocation drives asymmetry in the odontocete skull is that bats also echolocate and do not have cranial asymmetry as the natural condition [18]. However, the extreme differences in the environments in which bats and cetaceans echolocate, as well as other ecological and morphological differences between the two clades, complicate any meaningful comparison [85]. It should be noted that both odontocetes and bats share a remarkable convergence on narrow biosonar beams across species independent of body size [22, 86], with the ability to do this in odontocetes likely a result of cranial asymmetry.

With the most widely supported explanation of asymmetry being sound production, our results support the hypothesis that craniofacial asymmetry (along with concavity in the facial area, hypertrophied naso-facial muscles, air sacs, melon, and premaxillary sac fossa [26]) arose in odontocetes to support high-frequency echolocation. Further, echolocating in complex environments continues to be a primary factor driving the evolution of asymmetry in the odontocete skull, as supported by the independent evolutionary regimes for the most asymmetric odontocetes.

## Conclusions

Our study represents the first comprehensive analysis of cranial asymmetry spanning the evolutionary history of cetaceans. We demonstrate that the common ancestor of living cetaceans had little cranial asymmetry and thus is unlikely to have possessed the ability to echolocate. Odontocetes display increasing cranial asymmetry from the Oligocene to present, reaching their highest levels in extant taxa. Separate evolutionary regimes are supported for three odontocete clades (monodontids, physeteroids, and platanistids) that inhabit acoustically complex environments, suggesting that echolocation and cranial asymmetry are continuing to evolve under strong selection in these niches. Surprisingly, no increases in asymmetry were recovered within the highly asymmetric ziphiids. We attribute this to the extreme, asymmetric shape of the premaxillary crests and sac fossae in these taxa not being captured by landmarks alone.

Mysticetes have maintained a low level of cranial asymmetry since their origin, and if asymmetry reflects ultrasonic sound production ability, it is unlikely that mysticetes were ever able to echolocate. Archaeocetes have a high level of asymmetry in the rostrum which could be linked to directional hearing, as reported by Fahlke et al. [16], but this rostral asymmetry disappears in early neocetes as the dichotomous hearing abilities of the two suborders became established.

Modelling the evolution of cranial asymmetry across living and extinct cetaceans recovered the highest probabilities of shifts in the trait at three main points: first, in the extinct odontocete xenorophids in the Early-Mid Oligocene, then in the physeteroids (Late Oligocene), and finally in the monodontids in the Late Miocene/Early Pliocene. Smaller shifts were found in the Squalodelphinidae and Platanistidae. This was also true for ‘jumps’ in the trait, with an additional jump in a branch of the delphinids (namely the Globicephalinae e.g. pilot whales and false killer whales). Additional episodes of rapid change were found in the Mid-Late Oligocene, a period of rapid evolution in cranial asymmetry in odontocetes. These results support studies suggesting that biosonar, the signature adaptation of odontocetes, and associated asymmetry were acquired at or soon after the origin of this clade [4–6, 39].

## Methods

### Specimens

The data set comprises stem cetaceans (archaeocetes, *n* = 10) and both extant suborders: the baleen whales (mysticetes, *n* = 32) and toothed whales (odontocetes, *n* = 120). The final data set comprised 162 cetacean crania, of which 78 (48%) are extinct, ranging in age from 48.6 to 2.59 Mya. Additionally, 10 terrestrial artiodactyls (representing 7 of the 10 Arctiodactyla families) were included to provide a baseline for symmetry as cetaceans are nested within Artiodactyla. Specimen details (Additional file 1: Table S9) and museum abbreviations are provided in Additional file 1.

Specimens were selected to cover the widest possible phylogenetic spread, representing 38 families and 101 genera from the Eocene to the present.

The Early-Middle Eocene is represented by the land-dwelling family Pakicetidae through to semi-aquatic Ambulocetidae and Remingtonocetidae. The Pelagiceti are represented by the fully aquatic Basilosauridae of the Late Eocene through to the modern Neoceti. This includes representation of some early stem toothed mysticetes such as the Mammalodontidae and the Aetiocetidae. Three of the four extant mysticete families are represented. The odontocetes are represented by early stem families: the Xenorophidae and the Simocetidae of the Early-Mid Oligocene and the ‘Patriocetidae’ (phylogenetic position is still being clarified) of the Late Oligocene. The more crownward odontocetes of the Miocene are represented by the Eurhinodelphinidae, Kentriodontidae, Albireonidae, Squalodelphinidae, Squalodontidae, and Allodelphinidae among other extinct families. All ten extant odontocete families are represented. See Additional file 1: Table S9 for details.

Because many extant and all fossil specimens lack information on sex, sexual dimorphism could not be considered. All specimens are adult except for one subadult, *Mesoplodon traversii*. Specimens were selected based on completeness but some bones were broken (e.g. jugal) and were treated as missing data. Sixty-four (~ 39%) of the specimens, including some extant specimens, had missing data concentrated in the pterygoid, palatine, jugal, squamosal, and tip of the rostrum. For this reason and because fossils often have a higher proportion of missing data, we also ran analyses without any fossils and without rostral landmarks. Specimens with obvious taphonomic or other deformation were excluded from further analysis (Additional file 1: Table S10). Excluded specimens include the basilosaurid *Cynthiacetus peruvianus* which shows sinistral torsion in the rostrum. Although a potential natural feature in protocetids and basilosaurids [15, 16], it is suggested that rostral distortion in this particular specimen (MNHN.F.PRU10) is at least partly the original morphology of the skull and potentially a result of some taphonomic distortion [27]. Inevitably, some fossil specimens have sections of reconstructed bone. Their inclusion in the study was based upon the extent and accuracy of the reconstruction and the unavailability of alternative specimens.

**Fig. 6.**
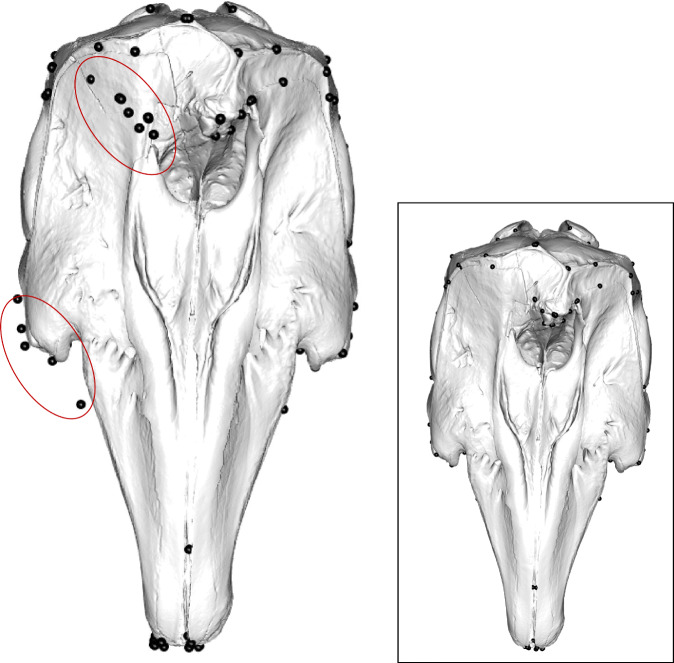
Misalignment of mirrored landmarks when using the mirrorfill function on a specimen without bilateral symmetry. Landmarks mirrored in the geomorph package [90] on an asymmetric specimen. Note the incorrect mirroring of landmarks on the nasal and to a lesser extent on the lateral point of the maxilla near the orbit (circled) in this specific specimen. Inset shows the same skull with the landmarks correctly placed. Specimen is *Delphinapterus leucas* USNM 305071

Skulls were scanned using a Creaform Go!SCAN 20, or Creaform Go!SCAN 50 depending on the size of the skull. Scans were cleaned, prepared, and merged in VX Elements v.6.0 and exported in ply format before being further cleaned and decimated in Geomagic Wrap software (3D Systems). Models were decimated down to 1,500,000 triangles, reducing computational demands without compromising on detail for further morphometric analyses. In many studies of morphology when the skull is incomplete, it is possible to digitally reconstruct bilateral elements by mirroring across the midline plane if preserved on one side [87–89]. However, due to the substantial asymmetry observed in many taxa in this study, mirroring a complete half of the skull was not possible (Fig. 6; Additional file 1: Fig. S15). For this reason, we limited mirroring to marginally damaged bones or easily mirrored missing bones only, where it was clear that mirroring would not mask any biological asymmetry, using the ‘mirror’ function in Geomagic Wrap (3D Systems).

### Morphometric data collection

#### Manually placed landmarks (*Fn*)

**Fig. 7.**
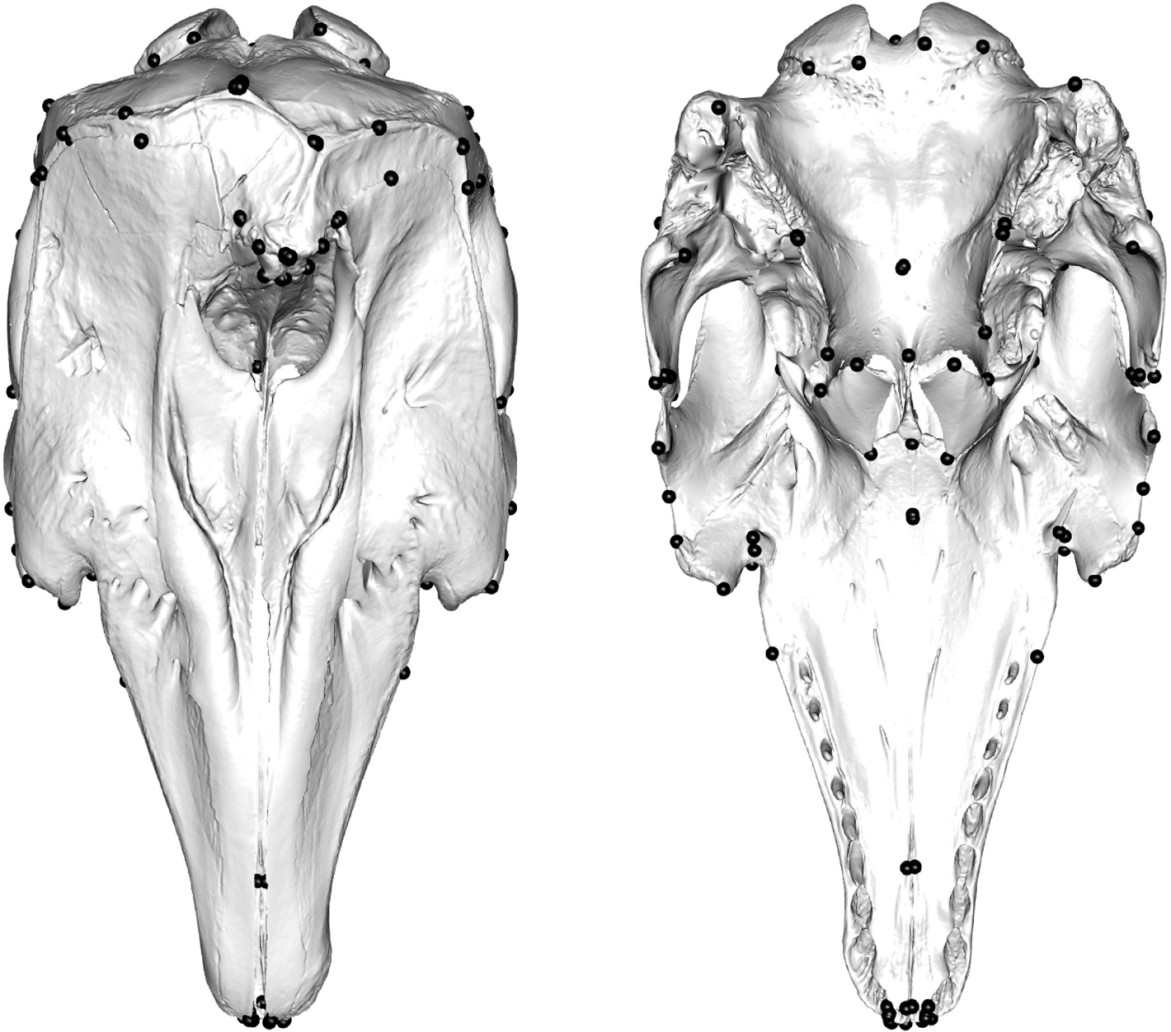
123 landmarks (in black) placed on the dorsal (**a**) and ventral (**b**) of the skull. 9 landmarks were placed on the midline (for landmark details, see Additional file 1: Table S11–123 landmarks added to the entire surface of the skull). Specimen is *Delphinapterus leucas* USNM 305071

We placed 123 anatomically defined landmarks over the surface of the skull in Stratovan Checkpoint (Stratovan, Davis, CA, USA) using the ‘single point’ option. We placed 57 landmarks on both the left-hand side (LHS) and right-hand side (RHS) of the skull, and 9 landmarks on the midline, totalling 123 landmarks covering both the dorsal and ventral sides of the skull (Fig. 7). Type I and II landmarks [91] were selected to comprehensively represent the full cranium (Fig. 7; Additional file 1: Table S11). ‘Landmark 15’ and the subsequent mirrored ‘landmark 79’ denote the back of the toothrow in most species. In some ziphiids, e.g. *Mesoplodon carlhubbsi*, the teeth (or tusks) erupt midway along the mandible [92] whilst other species present multiple pairs of tusks [93]. In others (e.g. *Hyperoodon ampullatus*), teeth typically erupt as a single pair on the anterior mandible which often protrudes *beyond* the upper jaw [92]. Without the mandible, it is challenging to pinpoint the positioning of the back of the toothrow, and even then, the presence and number of teeth is negligible in some species. Further, these tusks only erupt in adult males. For these reasons, and to avoid simply estimating where the true tooth row may be, ‘landmark 15’ and ‘landmark 79’ in specimens with mandibular prognathism, absent, maxillary-only, or vestigial dentition (including all ziphiids, narwhals (*Monodon monoceros)* and sperm whales (*Physeter macrocephalus*)) were consistently placed on the proximal lateral maxilla where the posterior end of a standard tooth row would be located (Additional file 1: Fig. S16).

As previously noted, some specimens have missing data. Geometric morphometric analyses and plotting functions implemented in geomorph v.3.1.0 [90] require a full complement of landmarks [90]. This complement can consist of actual landmarks and estimated positions for ‘missing’ landmarks. To estimate positions for missing landmarks, we placed ‘missing’ landmarks as close to the missing bone (areas that could not be digitally mirrored) as possible and then marked it as a ‘missing landmark’ in Checkpoint which automatically assigns a coordinate of − 9999. We then used the estimate.missing function in geomorph and the ‘TPS’ (thin plate spline) method to estimate the location of landmarks on incomplete specimens. A reference specimen which has a complete complement of landmarks is selected, and incomplete specimens are aligned against it using common landmarks [87]. In a TPS-based estimation, missing landmarks are placed so that the overall bending energy between the reference and the incomplete specimen is smallest which creates a smooth deformation [87]. TPS was chosen over regression-based methods (i.e. ‘Reg’ in geomorph) because it performs better in simulations with missing data [87].

#### Phylogeny

Our study uses a phylogenetic framework to reconstruct macroevolutionary patterns of cranial asymmetry across Cetacea. To generate a tree that included all of our sampled taxa, we used the time-calibrated phylogeny from Lloyd and Slater [29]. This ‘genus tree’ includes all species belonging to a genus that appear in a character matrix using taxonomic constraints to place taxa that lack data. We modified it as follows: First, we added several additional extant species (which were already represented to the genus level in the Lloyd and Slater phylogeny [29]) with position based on recently published studies. We placed *Neophocaena asiaeorientalis* in the same genus as *Neophocaena phocaenoides* [94], *Sousa plumbea* + *Sousa teuszii* + *Sousa sahulensis* in the same genus as *Sousa chinensis* [95], *Orcaella heinsohni* in the same genus as *Orcaella brevirostris* [96, 97], and *Mesoplodon hotaula* in the genus *Mesoplodon* next to *Mesoplodon gingkodens* [98]. Finally, we placed *Berardius minimus* in the genus *Berardius* next to *Berardius bairdii* and *Berardius arnuxii* following its recent description by Yamada et al. [78]. The following fossil species were directly swapped with their corresponding monophyletic congener as follows. We placed *Balaneoptera* sp. (SDNHM 83695) as a sister taxon to *Balaenoptera siberi* (although not present in our sample), close to extant *Megaptera novaeangliae* as in Martin [99], *Balaenoptera floridana* as a sister taxon to *Balaenoptera davidsonnii* [100, 101] (again, the latter species is not present in our sample), and *Orycterocetus crocodilinus* is placed in the physeterids according to Lambert et al. [102]. We placed *Globicephala* sp*.* as a sister taxon to *Globicephala etruriae* [103–105] and *Hemisyntrachelus cortesii* in the same genus as *Hemisyntrachelus oligodon* according to Post and Bosselaers [106]. We caution that Kentriodontidae is often considered a non-monophyletic ‘waste-basket’ for Late Oligocene and Miocene homodont odontocetes [107]. Restrictions according to Peredo et al. [108] leave *Tagicetus* and *Atocetus* (previously referred to as Kentriodontidae) outside of the family (Additional file 1: Table S9). The positioning of *Argyrocetus joaquinensis* is also unclear [109]. Two *s*pecimens (*Xenorophus* ChM PV7677 and Patriocetid or Waipatiid CCNHM 1078) were excluded from the analysis due to uncertainty in their position (Additional file 1: Table S10).

### Data analysis

#### Quantifying asymmetry

We generated mirrored landmarks for the right-hand side (RHS) of the skull and compared their positions to those of the original manually placed landmarks, measuring the amount of landmark displacement between the two. To do so, we used the 57 LHS landmarks and 9 midline landmarks (total = 66) (Additional file 1: Table S11) and mirrored the LHS landmarks onto the RHS using the mirrorfill function in the R package paleomorph v.0.1.4 [110]. Before carrying out further analyses, we superimposed the specimens to remove all non-shape elements, i.e. size (scaling), translation, and rotation (positioning) from the data using Generalized Procrustes Analysis implemented in the gpagen function from the geomorph R package v.3.1.0 [90].

We used the R package landvR v.0.4 [111] to calculate the Euclidean distances between a reference specimen (the computer -mirrored, landmarked specimen) (*Rn*) and a focal specimen (the manually landmarked specimen) (*Fn*). Both *Rn* and *Fn* are defined by three coordinates (*x*, *y*, *z*). The landmark displacements were measured for each landmark individually using the spherical coordinates system which measures between the *n*^th^ landmark of the *Fn* and the *Rn* specimens respectively [111]. This method provides 3 outputs (from Guillerme and Weisbecker [111]):

- *ρ*, the Euclidean distance between *Fn* and *Rn*
- *ϕ*, the azimuth angle formed by the projection of *Rn* on the equatorial plane *(f(x) = 0)*
- *θ* (when using 3D data only), the polar angle formed by the projection *Rn* on the polar plane *(f(y) = 0)*

We estimated differences between *Fn* and *Rn* in the spherical coordinates system using the coordinates.difference function in landvR and extracted the *ρ* (radius) for each landmark, for each specimen. This provides a measure of the Euclidean distance between a manually placed landmark which accurately represented the specimen’s morphology (*Fn)* and a computer -mirrored landmark (*Rn)*. If the specimen is asymmetric, the computer -mirrored landmark does not accurately reflect its morphology (Fig. 6).

**Fig. 8.**
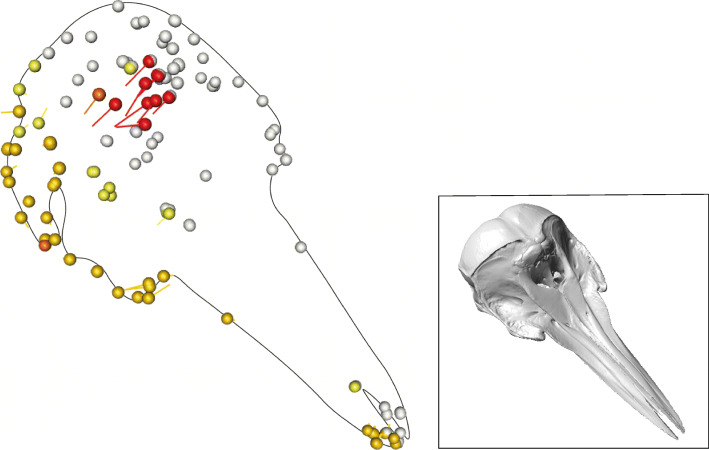
Visualisation of *p* (radii) from landvR showing asymmetry in the dolphin skull. Landmarks are placed on a stylised outline of a dolphin skull. The 3D surface scan of a dolphin skull (inset) is shown for orientation and is *Lissodelphis borealis* USNM 550188. The white spheres on the landvR output show the fixed landmarks (1–66) on the left-hand side (LHS) of the skull (looking down on the skull with the rostrum pointing north). The landmarks on the right-hand side (RHS) of the skull vary in colour depending on how much difference there is between a computer -mirrored landmark (*Rn*) (which assumes the skull is bilaterally symmetrical) and a manually placed landmark (*Fn*) (which accurately depicts asymmetry). The larger the difference between the computer -mirrored landmark and the manually placed landmark, the hotter the colour. The highest amount of asymmetry is shown in red and dark orange, less asymmetry is shown in pale orange and yellow. Note the red landmarks on the nasal and posterior premaxilla of this odontocete. The tails coming from each of the landmarks show how much and in which direction the landmarks have moved from where the computer mirrored them, to where the landmarks sit when manually placed

The spherical coordinates system is preferable because it directly measures landmark displacement in any direction, and further, the values for each landmark displacement is discrete in space (i.e. independent from other landmarks) [111]. This is important because it allows identification of asymmetry which may occur in discrete parts of the skull, e.g. the posterior nasal without blanket labelling all landmarks as ‘asymmetric’. We obtained *ρ* for each of the 123 landmarks for each specimen, including the terrestrial artiodactyls (21,156 radii values in total). The larger the radii (and consequently the larger the *ϕ* for a corresponding landmark) the more displacement between *Fn* and *Rn.* We interpret a higher *ρ* as an indication of more asymmetry in the skull (see Fig. 8 for a visualisation of this). Higher displacement means that there is a greater difference between the placement of *Fn* and *Rn,* indicating asymmetry in those landmarks [111]. The closer the radius to 0, the more symmetrical the specimen as *Fn* has not displaced far from *Rn*. We took the averaged sum of radii for each landmark (x̄*ρ*_land_) to find the most asymmetrical landmarks and identify their location on the skull for each group (archaeocetes, odontocetes, mysticetes, and terrestrial artiodactyls) (Table 2), as well as an average total cranial asymmetry (x̄*ρ*) for each group. We also took the sum radii for each of the individual specimens (Σ*ρ*_spec_). We ran a principal component analysis (PCA) on the Procrustes aligned data using the ‘factoextra’ package v.1.0.7 [112] in R to identify PC scores of maximum radii variation using the sum radii for each of the individual specimens (Σ*ρ*_spec_) (Fig. 2). We then used overall asymmetry in each specimen (Σ*ρ*_spec_) to reconstruct the evolution of cranial asymmetry.

For computational purposes, all polytomies in the tree were resolved by adding zero branch lengths using multi2di in ape v.5.0 [113] prior to downstream phylogenetic analyses. All models were run with cetacean data only (i.e. no terrestrial artiodactyls), and model fit was assessed using AIC. We also conducted the analyses after removing the rostrum (NR) as it may be more easily deformed through both taphonomic deformation and drying out in extant specimens. We also ran all models with a phylogeny that includes only taxa that appear in a character matrix from Lloyd and Slater [29]. We found no differences in the ordering of the best-fitting models with NR, nor with the phylogeny which only uses taxa that appear in the character matrix (see Additional file 1: Figs. 1-3, 11–13; Tables S5a-c); we thus focus the analyses on data including the rostral landmarks and the original ‘genus tree’.

#### Modelling the evolution of cranial asymmetry

We assessed how asymmetry has evolved across Cetacea using phylogenetic models of trait evolution. We investigated variation in rates of cranial asymmetry evolution using a relaxed Brownian motion (BM) process with the rjmcmc.bm function implemented in the R package geiger v.2.0.6.4 [114, 115]. This model uses a Bayesian framework with a reversible jump Markov Chain Monte Carlo (rjMCMC) algorithm to find the position and the amplitude of evolutionary rates changes and ‘shifts’ across the tree [115]. ‘Jumps’ indicate a temporary or rapid change in the trait.

We ran the rjMCMC chain for 10^6^ generations sampling each 10,000 generations with the combined ‘jump-rbm’ class model [115]. We used the weakly informative Half-Cauchy distribution with scale parameter 25 [35] as the prior density of the rate scalar and measurement error instead of the default exponential distribution and used default priors for the number of shifts (a Poisson distribution with mean equal to log(2) which places a 50% probability on a scenario with no shifts). For comparison and as a proposal mechanism for exploring the parameter space, we ran the same model with the ‘rbm’ class model and no jumps. We checked the effective sample size (ESS) and assessed convergence of the chains with Gelman and Rubin’s diagnostics [35, 36] using the ‘effectiveSize’ and ‘gelman.diag’ functions implemented in the R package coda [34] (Additional file 1: Figs. S6–10; Table S6 [34]).

#### Hypothesised evolutionary regimes for cranial asymmetry

Several state-dependent models were proposed as potential predictors for the level of asymmetry seen in the cetacean skull. For example, ‘echolocation’ (model: ‘echo’) (Table 4) is one model used to investigate whether the rate of evolution for skull asymmetry differs between species that can echolocate and those that cannot. We name two other models, the regime model (model: ‘regime’) and the ‘regime split’ model (model: ‘regime-split’). In these models we test whether evolutionary changes in asymmetry in the cetacean cranium (the studied trait) can be associated with the states of another discrete trait. By regime, we mean a particular condition or process that may be underlying the observed patterns of cranial asymmetry. We further fitted a ‘frequency echolocation’ model (model: ‘echo-freq’) (Table 4; Additional file 1: Table S8 [40–42, 45–54, 64, 70–72]). This study is not a specialist analysis of acoustics (nor behaviours affecting acoustics) in cetaceans, and we use these values to *indicate* potential drivers for the evolution of cranial asymmetry. We assigned species to several categories depending on how they predominantly produce sound (Table 4; Additional file 1: Table S8 [40–42, 45–54, 64, 70–72]). We fitted our several state-dependent models with different variants of the BM and OU model (Table 5). The multiple models and different rates are summarised below.

**Table 4:** Models testing whether changes in cetacean cranial asymmetry are associated with other discrete traits

Scenario (model name)	Description	Model assumptions and references
Ancestral state reconstruction (‘ancestral’)	Species belong to one of three ancestral categories: ‘archaeocete’, ‘odontocete’, and ‘mysticete’	The placing of species into ‘archaeocete’, ‘odontocete’, and ‘mysticete’ was based on the literature and published fossil descriptions [26, 37]
‘Regime’ model (‘regime’)	Assumes selective evolutionary regimes. Archaeocetes are assigned to ‘regime1’, mysticetes to ‘regime2’, and most odontocetes to ‘regime3’. The highly asymmetric monodontids, platanistids, and superfamily physeteroids are classified as a separate ‘regime4’	Regimes are based on a preliminary trait plot (Fig. 3) which shows that the monodontids, platanistids, and superfamily physeteroids have a much higher trait value (sum radii for the individual specimen (Σ*ρ*_spec_)) (≥ 0.42, Fig. 3) than other odontocetes and therefore may be evolving asymmetry under one different selective regime
‘Regime-split’ model (‘regime-split’)	As in the regime model, archaeocetes are assigned to ‘regime1’, mysticetes to ‘regime2’, odontocetes in general to ‘regime3’, and the highly asymmetric odontocetes (monodontids, platanistids, and physeteroids) are placed in their own separate selective regimes	Each highly asymmetric group is evolving under its own separate selective regime: (1) monodontids, (2) platanistids, and (3) physeteroids
Echolocation model (‘echo’)	Species assigned to one of four groups depending on whether the species could echolocate Band0: Cannot echolocate Band1: Not capable of echolocation, although reception of ultrasonic signals cannot be ruled out Band2: Early echolocation, e.g. *Cotylocara macei* [4] and *Echovenator* [5, 38] Band3: Fully echolocating	i. Although rudimentary, echolocation evolved very early in whale evolution, likely soon after odontocetes diverged from the ancestors of baleen whales [4] ii. The ability to produce ultrasonic sounds, and therefore echolocate, has been inferred for almost all fossil odontocetes [9] although *Odobenocetops* likely had greatly reduced echolocation abilities [26] iii. Mysticetes do not echolocate iv. All extant odontocetes echolocate [39]
Echolocation-frequency model (‘echo-freq’)	Categorising by echolocation in the extant odontocetes and sound production in the extant mysticetes	i. Data on frequency specifics is not available for fossils ii. Narrowband high-frequency (NBHF) cetaceans designated according to Kastelein et al. [40] and Khyn et al. [41, 42] iii. The non-NBHF delphinids were assigned to broadband low frequency (BBLF) according to Jensen et al. [43] and Turl et al. [44] iv. The sperm whale sits in its own category. The hypertrophied nasal structures and deep-diving behaviour produce a low-frequency multi-pulsed sound [45] v. Ziphiids sit in their own category. They produce frequency-modulated buzz clicks (FM-buzz) [46–50] vi. Mysticetes do not echolocate and produce low-frequency sound [24, 51] vii. The Monodontidae sit in their own category. They produce narrowband structured (NBS) pulses [52–54] See Additional file 1: Table S8 for further details

Models tested to assess whether evolutionary changes in asymmetry in the cetacean cranium are associated with the states of another discrete trait. The ‘scenario’ names the type of model fitted, for example the echolocation model is based on whether a cetacean can echolocate or not. The description and assumptions outline the conventions of the model

**Table 5:** Models implemented using a maximum-likelihood inference to test evolutionary models for changes in asymmetry

Model name	State	Model type	Description
‘OU-ancestral’	Ancestral state	OU	A classic Ornstein-Ulenbeck (OU) model
‘BM-ancestral’	Ancestral state	BM	A classic Brownian motion (BM) model
‘BMtr-ancestral’	Ancestral state	BMtr	A classic BM model with an independent trend
‘BMsm-ancestral’	Ancestral state	BMsm	A classic BM model with no selective regime and which estimates separate phylogenetic means
‘BMM-ancestral’, ‘BMM-regime’, ‘BMM-regime-split’, ‘BMM-echo’, BMM-echo-freq’	Ancestral state, regime, regime-split, echolocation, echolocation-frequency	BMM	A BM model with a selective regime
‘BMMtr-ancestral’, ‘BMMtr-regime’, ‘BMMtr-regime-split’, ‘BMMtr-echo’, BMMtr-echo-freq’	Ancestral state, regime, regime-split, echolocation, echolocation-frequency	BMMtr	A BM model with a selective regime and an independent trend
‘BMMsm-ancestral’, ‘BMMsm-regime’, ‘BMMsm-regime-split’, ‘BMMsm-echo’, BMMsm-echo-freq’	Ancestral state, regime, regime-split, echolocation, echolocation-frequency	BMMsm	A BM model with a selective regime which estimates separate phylogenetic means
‘OUM-ancestral’, ‘OUM-regime’, ‘OUM-regime-split’, ‘OUM-echo’, ‘OUM-echo-freq’	Ancestral state, regime, regime-split, echolocation, echolocation-frequency	OUM	An OU model with a selective regime

Models test whether evolutionary changes in asymmetry (the studied trait) are associated with the states of another discrete trait. The model name is a combination of the model state and the model type and is used throughout the study for consistency. The state describes the model scenario. The model types are variations of an Ornstein-Uhlenbeck (OU) model of continuous trait evolution and a Brownian motion (BM) model of continuous trait evolution (see description). All models were run using an ‘equal-rates’ (ER) likelihood model (Additional file 1: Table S12—likelihood model results (AIC) for each potential scenario for asymmetry in the cetacean cranium). For details on the model assumptions, see Table 4—models testing whether changes in cetacean cranial asymmetry are associated with other discrete traits

In addition, we evaluated the fit of 24 alternative models (all listed in Table 5) based on the states of a discrete character (Table 4) implemented in the mvMORPH package in R [116] using a maximum likelihood inference. We used the ‘fitDiscrete’ function in ‘geiger’ v.1.3-1 to fit various likelihood models for discrete character evolution. The model arguments tested were an ‘equal-rates’ model (ER) where all transitions occur at equal rates, a ‘symmetric transitions are equal’ model (SYM), and an ‘all rates different’ model (ARD) where each rate is a separate parameter [114, 117]. The ER model gave the best fit (Additional file 1: Table S12 [114, 117]) and was thus used in all of our alternative models using maximum-likelihood inference (Table 5).

The multiple models described above (Table 4) relax the assumption of a common dynamic for modelling the trait evolution by allowing the estimation of the model parameters that depend on the states of a discrete character. For these, we first had to ‘paint’ the evolutionary history (e.g. ancestral state) of the selective regime onto the tree. To do this, we used write.simmap in the phytools package v.0.6–99 [118]. We ran these models under OUM and BMM assumptions (Table 5). We repeated these analyses for the data with the rostrum removed (Table 3) and with the phylogeny that includes only taxa that appear in a character matrix [29] (Additional file 1: Table S5b-c [29]). All analyses were done in R v.3.5.0 [119].

Although the relaxed BM process described above is very flexible and allows the investigation of changes in evolutionary rates across the tree without strong a priori, it is however limited for assessing and interpreting changes in evolutionary modes. Moreover, recovered changes in rates might result from long-term trends in the average asymmetry rather than actual changes in the pace of evolution. We considered multiple models including parameterizations of the BM and OU process (Table 5). Our models assume constant dynamics of trait evolution but a directional drift of the clade average value that might be interpreted as shifts in evolutionary rates in the relaxed BM model considered above. We also considered models with specific optimums (model “OUM” in ‘*mvOU’*), and ancestral states and/or rates (model “BMM” in ‘*mvBM*’) in different parts of the tree. The more parameterized and refined models allow for testing of evolutionary changes in the studied trait and can be associated with the states of another discrete trait. In this study, we consider different scenarios to assess whether the evolution of the skull asymmetry shows marked differences between the three major clades (archaeocetes, odontocetes, and mysticetes) and if it is related to the evolution of echolocation.

#### Analysis of variance

Lastly, we ran phylogenetically corrected ANOVAs on each of the different scenarios using the R package nlme (v.3.1-137) [120] and function ‘gls’ to test for correlations between the level of asymmetry seen in the skull and the potential scenarios (or regimes) hypothesised above (Table 4). ‘gls’ allows for a more flexible model with better power. Simulations were run using a ‘Pagel’s Lambda’ (λ) correlation structure (corPagel) in the ape package [113] by multiplying the off-diagonal of the phylogenetic covariance matrix by "lambda" constrained to be within [0, 1]. As we ran multiple models, we controlled for a false discovery rate using the Benjamini-Hochberg method [55, 56] (Additional file 1: Table S7 [55, 56]). The data sets generated and/or analysed during the current study are available in the Github repository: https://github.com/EllenJCoombs/Asymmetry-evolution-cetaceans [121].

## Supplementary information

**Additional file 1 : Tables S1–12, Figure S1-S16. Table S1.** All specimens ranked by sum radius (Σ*ρ*_spec_). **Table S2.** Percentage of asymmetry in the rostrum – archaeocetes. **Table S3.** Percentage of asymmetry in the rostrum – mysticetes. **Table S4.** Percentage of asymmetry in the rostrum – odontocetes. **Figure S1.** Asymmetry in the cetacean skull with the rostrum removed. **Figure S2.** Reconstructed probability of shifts in cetacean cranial asymmetry (∑*p*_spec_) with the rostrum removed. **Figure S3.** Reconstructed jumps in the rate of cetacean cranial asymmetry (∑*p*_spec_) with the rostrum removed. **Table S5**. Akaike information criterion (AIC) rankings for each evolutionary model for asymmetry in the cetacean cranium. **Figure S4.** Additional morphospace occupation of cetacean crania used in this study. **Figure S5.** Principal Components plot with PC1 and PC2 for each specimen in the study. **Model diagnostics**. **Figure S6.** Trace of the chain for model 1. **Figure S7.** Trace of the chain for model 2. **Figure S8.** Further model diagnostics for chain 1. **Figure S9.** Further model diagnostics for chain 2. **Table S6.** Effective size (ES) for estimating the mean for each of the chains 1 and 2. **Figure S10.** Gelman diagnostics for the two chains. **Figure S11.** Asymmetry in the cetacean skull shown using a phylogeny that includes only taxa that appear in a character matrix. **Figure S12.** Reconstructed probability of shifts in cetacean cranial asymmetry (∑*p*_spec_) using a phylogeny that includes only taxa that appear in a character matrix. **Figure S13.** Reconstructed jumps in the rate of cetacean cranial asymmetry (∑*p*_spec_) using a phylogeny that includes only taxa that appear in a character matrix. **Table S7.** ANOVA results for each potential scenario for asymmetry in the cetacean cranium. **Table S8.** Frequency categories used to group all extant cetaceans for the ‘frequency echolocation’ model. **Figure S14.** Ziphiid skulls showing the marked asymmetry in the premaxillary crests. **Table S9.** List of specimens used in the study. **Table S10.** Skulls scanned but excluded from analysis. **Figure S15.** The landmark configuration with manually placed landmarks on half of the skull to be mirrored to the other half of the skull. **Table S11.** 123 landmarks added to the entire surface of the skull. **Figure S16.** The position of landmark 15 (to be mirrored as landmark 79). **Table S12.** Likelihood model results (AIC) for each potential scenario for asymmetry in the cetacean cranium.

## Data Availability

The data sets generated and/or analysed during the current study are available in the Github repository: https://github.com/EllenJCoombs/Asymmetry-evolution-cetaceans [121]. All code and raw data are available to download, complete with MIT licence. Please cite this paper and the Zenodo doi: 10.5281/zenodo.3893943 when using the data or raw code. See Github for details.

## Abbreviations

AIC: Akaike information criterion
ANOVA: Analysis of variance
ARD: ‘All rates different’ model
BM: Brownian motion
ER: ‘Equal-rates’ model
FM-buzz: Frequency-modulated buzz clicks
*Fn*: Manually placed landmark
Mya: Million years ago
NBHF: Narrowband high-frequency
NBS: Narrowband structured
NR: No rostrum
OU: Ornstein-Uhlenbeck
*p*: Radius
rjMCMC: Reversible jump Markov Chain Monte Carlo
*Rn*: Computer -mirrored landmark
SYM: ‘Symmetric transitions are equal’ model
x̄*ρ*: Average of the total radii (Σ*ρ*) values across the skull
x̄*ρ*_land_: Averaged sum of radii for each landmark
Σ*ρ*_spec_: Sum radii for each of the individual specimens
Σ*ρ*_land_: Sum radii for specific landmarks
