# Supplementary material for: Wonky whales: the evolution of cranial asymmetry in cetaceans
Source: BMC Biol. 2020 Jul 10;18:86. doi: 10.1186/s12915-020-00805-4 (PMC7350770; doi:10.1186/s12915-020-00805-4)
Supplement: Supplementary file 1 — Additional file 1 : Tables S1–12, Figure S1-S16. Table S1. All specimens ranked by sum radius (Σρspec). Table S2. Percentage of asymmetry in the rostrum – archaeocetes. Table S3. Percentage of asymmetry in the rostrum – mysticetes. Table S4. Percentage of asymmetry in the rostrum – odontocetes. Figure S1. Asymmetry in the cetacean skull with the rostrum removed. Figure S2. Reconstructed probability of shifts in cetacean cranial asymmetry (∑pspec) with the rostrum removed. Figure S3. Reconstructed jumps in the rate of cetacean cranial asymmetry (∑pspec) with the rostrum removed. Table S5. Akaike information criterion (AIC) rankings for each evolutionary model for asymmetry in the cetacean cranium. Figure S4. Additional morphospace occupation of cetacean crania used in this study. Figure S5. Principal Components plot with PC1 and PC2 for each specimen in the study. Model diagnostics. Figure S6. Trace of the chain for model 1. Figure S7. Trace of the chain for model 2. Figure S8. Further model diagnostics for chain 1. Figure S9. Further model diagnostics for chain 2. Table S6. Effective size (ES) for estimating the mean for each of the chains 1 and 2. Figure S10. Gelman diagnostics for the two chains. Figure S11. Asymmetry in the cetacean skull shown using a phylogeny that includes only taxa that appear in a character matrix. Figure S12. Reconstructed probability of shifts in cetacean cranial asymmetry (∑pspec) using a phylogeny that includes only taxa that appear in a character matrix. Figure S13. Reconstructed jumps in the rate of cetacean cranial asymmetry (∑pspec) using a phylogeny that includes only taxa that appear in a character matrix. Table S7. ANOVA results for each potential scenario for asymmetry in the cetacean cranium. Table S8. Frequency categories used to group all extant cetaceans for the ‘frequency echolocation’ model. Figure S14. Ziphiid skulls showing the marked asymmetry in the premaxillary crests. Table S9. List of specimens used in the study. Tab [file 12915_2020_805_MOESM1_ESM.docx]

**The following supplemental accompanies the article:**

**Wonky whales: The evolution of cranial asymmetry in cetaceans**

Ellen J. Coombs^1,2^*, Julien Clavel^3^, Travis Park^2,4^, Morgan Churchill^5^, Anjali Goswami^1,2, 6^

1 University College London, Genetics, Evolution and Environment Department, Gower Street, WC1E 6BT, UK.

2 Department of Life Sciences, Natural History Museum, London, Cromwell Road, London, SW7 5BD. UK.

3 Univ Lyon, Université Claude Bernard Lyon 1, CNRS, ENTPE, UMR 5023 LEHNA, F-69622, Villeurbanne, France.

4 Department of Earth Sciences, University of Oxford, Oxford, United Kingdom, OX1 3AN, UK.

5 Department of Biology, University of Wisconsin-Oshkosh, Oshkosh WI 54901, USA.

6 Department of Earth Sciences, University College London, Gower Street, London WC1E 6BT, UK.

*Corresponding author

**Contents page**

[List of museum abbreviations 3](#_Toc41483728)

[Table S1. All specimens ranked by sum radius (Σ*ρ*_spec_). 3](#_Toc41483729)

[Table S2. Percentage of asymmetry in the rostrum – archaeocetes. 8](#_Toc41483730)

[Table S3. Percentage of asymmetry in the rostrum – mysticetes. 9](#_Toc41483731)

[Table S4. Percentage of asymmetry in the rostrum – odontocetes. 9](#_Toc41483733)

[Fig S1. Asymmetry in the cetacean skull with the rostrum removed. 14](#_Toc41483734)

[Fig S2. Reconstructed probability of shifts in cetacean cranial asymmetry (∑*p*_spec_) with the rostrum removed 16](#_Toc41483735)

[Fig S3. Reconstructed jumps in the rate of cetacean cranial asymmetry (∑*p*_spec_) with the rostrum removed. 18](#_Toc41483736)

[Table S5. Akaike information criterion (AIC) rankings for each evolutionary model for asymmetry in the cetacean cranium. 18](#_Toc41483737)

[Fig S4. Additional morphospace occupation of cetacean crania used in this study. 22](#_Toc41483738)

[Fig S5. Principal Components plot with PC1 and PC2 for each specimen in the study. 24](#_Toc41483739)

[Model diagnostics 25](#_Toc41483740)

[Fig S6. Trace of the chain for model 1 25](#_Toc41483741)

[Fig S7. Trace of the chain for model 2 26](#_Toc41483742)

[Fig S8. Further model diagnostics for chain 1. 28](#_Toc41483743)

[Fig S9. Further model diagnostics for chain 2. 30](#_Toc41483744)

[Table S6. Effective size (ES) for estimating the mean for each of the chains 1 and 2. 31](#_Toc41483745)

[Fig S10. Gelman diagnostics for the two chains 32](#_Toc41483746)

[Fig S11. Asymmetry in the cetacean skull shown using a phylogeny that includes only taxa that appear in a character matrix. 34](#_Toc41483747)

[Fig S12. Reconstructed probability of shifts in cetacean cranial asymmetry (∑*p*_spec_) using a phylogeny that includes only taxa that appear in a character matrix. 36](#_Toc41483748)

[Fig S13. Reconstructed jumps in the rate of cetacean cranial asymmetry (∑*p*_spec_) using a phylogeny that includes only taxa that appear in a character matrix 38](#_Toc41483750)

[Table S7. ANOVA results for each potential scenario for asymmetry in the cetacean cranium. 39](#_Toc41483751)

[Table S8. Frequency categories used to group all extant cetaceans for the ‘frequency echolocation’ model. 40](#_Toc41483752)

[Fig S14. Ziphiid skulls showing the marked asymmetry in the premaxillary crests 41](#_Toc41483753)

[Table S9. List of specimens used in the study 41](#_Toc41483754)

[Table S10. Skulls scanned but excluded from analysis 46](#_Toc41483755)

[Fig S15. The landmark configuration with manually placed landmarks on half of the skull to be mirrored to the other half of the skull 47](#_Toc41483756)

[Table S11. 123 landmarks added to the entire surface of the skull. 47](#_Toc41483757)

[Fig S16. The position of landmark 15 (to be mirrored as landmark 79) 50](#_Toc41483758)

[Table S12. Likelihood model results (AIC) for each potential scenario for asymmetry in the cetacean cranium 51](#_Toc41483759)

# **List of museum abbreviations**

AMNH – American Museum of Natural History

CCNHM - College of Charleston Natural History Museum

ChM - Charleston Museum

FMNH – Field Museum Natural History

GSM - Georgia Southern Museum

GSP-UM - Geological Survey of Pakistan, University of Michigan

IRSNB - Institute Royal des Sciences Naturelle’s de Belgique

LACM – Natural History Museum of Los Angeles County

MB - Berlin Museum für Naturkunde

MBGPT - Museo Regionale di Storia Naturale di Torino

MLP - Museo de La Plata

MNHN - Muséum national d'Histoire naturelle

MSNTUP – Museo di Storia Naturale e del Territorio, Univer sità di Pisa

MSNUP - Museo di Storia Naturale di Pisa - Università di Pisa

NHMUK – Natural History Museum UK

NMNS-PV - National Museum of Nature and Science, Tsukuba

NMNZ - Museum of New Zealand Te Papa Tongarewa

NMV - National Museum of Victoria

OU – University of Otago

PV M – Museum of Paleontology, University of Michigan

SDNHM – San Diego Museum Natural History

SMNK PAL- Staatliches Museum für Naturkunde Karlsruhe

SMNS - Staatliches Museum für Naturkunde Stuttgart

UCMP - University of California Museum of Paleontology

[Next page]

Table S1. All specimens ranked by sum radius (Σ*ρ*_spec_). Odontocetes in green, mysticetes in blue, archaeocetes in orange, and terrestrial artiodactyls in yellow. Note how the top rankings are dominated by the odontocetes, and the bottom rankings by the mysticetes and terrestrial artiodactyls.

| Rank | Specimen | Family | Suborder | sum radius  (Σ*ρ*_spec_) |
| --- | --- | --- | --- | --- |
| 1 | *Monodon monoceros* USNM 267959 | Monodontidae | odontocete | 0.546 |
| 2 | *Orycterocetus crocodilinus* USNM 22926 | Physeteridae | odontocete | 0.518 |
| 3 | *Aulophyseter morricei* UCMP 81661 | Physeteridae | odontocete | 0.489 |
| 4 | *Kogia breviceps* USNM 22015 | Kogiidae | odontocete | 0.462 |
| 5 | *Kogia simus* NHM.1952.8.28.1 | Kogiidae | odontocete | 0.457 |
| 6 | *Physeter macrocephalus* NHM 2007.1 | Physeteridae | odontocete | 0.456 |
| 7 | *Delphinapterus leucas* USNM 305071 | Monodontidae | odontocete | 0.453 |
| 8 | *Platanista gangetica* USNM 172409 | Platanistidae | odontocete | 0.449 |
| 9 | *Globicephala melas* NMNZ MM001946 | Delphinidae | odontocete | 0.410 |
| 10 | *Pseudorca crassidens* USNM 11320 | Delphinidae | odontocete | 0.408 |
| 11 | *Globicephala macrorhynchus* NHM 1912.10.27 | Delphinidae | odontocete | 0.407 |
| 12 | *Tagicetus joneti* IRSNB M. 1892 | Delphinida | odontocete | 0.406 |
| 13 | *Notocetus vanbenedeni* MLP 55 | Squalodelphinidae | odontocete | 0.404 |
| 14 | *Albertocetus* ChM PV8680 | Xenorophidae | odontocete | 0.394 |
| 15 | *Albireo whistleri* UCR 14589 | Albireonidae | odontocete | 0.393 |
| 16 | *Brachydelphis mazeasi* MNHN.F.PPI. 266 | Pontoporiidae | odontocete | 0.382 |
| 17 | *Septemtriocetus bosselaersi* IRSNB M.1928 | Phocoenidae | odontocete | 0.382 |
| 18 | *Orcaella brevirostris* NHM.1883.11.20.2 | Delphinidae | odontocete | 0.374 |
| 19 | *Squalodon calvertensis* NMNZ MM001996 | Squalodontidae | odontocete | 0.372 |
| 20 | *Feresa attenuata* USNM 504916 | Delphinidae | odontocete | 0.369 |
| 21 | *Lagenorhynchus cruciger* NHMUK 1960.8.24.1 (Sagmatias) | Delphinidae | odontocete | 0.368 |
| 22 | *Indopacetus pacificus* USNM 593534 | Ziphiidae | odontocete | 0.363 |
| 23 | *Kampholophus serrulus* UMCP 36045 | Kentriodontidae | odontocete | 0.357 |
| 24 | *Lagenorhynchus albirostris* AMNH 37162 | Delphinidae | odontocete | 0.355 |
| 25 | *Hemisyntrachelus cortesii* MBGPT NN | Delphinidae | odontocete | 0.354 |
| 26 | *Orcaella heinsohni* USNM 284430 | Delphinidae | odontocete | 0.350 |
| 27 | *Grampus griseus* USNM 571602 | Delphinidae | odontocete | 0.343 |
| 28 | Shark-toothed *squalodon* OU 21798 | Squalodontidae | odontocete | 0.340 |
| 29 | *Neophocaena phocaenoides* NHM 1903.9.12.3 | Phocoenidae | odontocete | 0.328 |
| 30 | *Lagenodelphis hosei* USNM 571619 | Delphinidae | odontocete | 0.328 |
| 31 | *Messapicetus longirostris* MSNUP NN | Ziphiidae | odontocete | 0.327 |
| 32 | *Zarhachis flagellator* USNM 10911 | Platanistidae | odontocete | 0.325 |
| 33 | *Globicephala* sp USNM 21867 | Delphinidae | odontocete | 0.324 |
| 34 | *Neophocaena asiaeorientalis* USNM 240001 | Phocoenidae | odontocete | 0.323 |
| 35 | *Peponocephala electra* USNM 504511 | Delphinidae | odontocete | 0.322 |
| 36 | *Lomacetus ginsburgi* MNHN.F.PPI.104 | Phocoenidae | odontocete | 0.322 |
| 37 | *Cephalorhynchus eutropia* NHM 1881.8.17.1 | Delphinidae | odontocete | 0.321 |
| 38 | *Kentriodon pernix* USNM 10670 | Kentriodontidae | odontocete | 0.320 |
| 39 | *Stenella longirostris* USNM 395270 | Delphinidae | odontocete | 0.320 |
| 40 | *Phocoena spinipinnis* NHM 1900.5.7.29 | Phocoenidae | odontocete | 0.320 |
| 41 | *Phocoena sinus* SDNHM 20697 | Phocoenidae | odontocete | 0.309 |
| 42 | *Lagenorhynchus australis* 1944.11.30.1 (Sagmatias) | Delphinidae | odontocete | 0.309 |
| 43 | *Hyperoodon ampullatus* NHM 1992.42 | Ziphiidae | odontocete | 0.308 |
| 44 | *Basilosaurus isis* SMNS 11787 | Basilosauridae | archaeocete | 0.308 |
| 45 | *Zygorhiza kochii* USNM 11962 | Basilosauridae | archaeocete | 0.306 |
| 46 | *Lissodelphis peronii* NMNZ MM002116 | Delphinidae | odontocete | 0.304 |
| 47 | Balaenopteridae NMNZ MM001630 | Balaenopteridae | mysticete | 0.300 |
| 48 | *Protocetus atavus* SMNS 11084 | Protocetidae | archaeocete | 0.298 |
| 49 | *Dorudon atrox* PV M 100149 | Basilosauridae | archaeocete | 0.298 |
| 50 | *Aglaocetus moreni* FMNH P13407 | Aglaocetidae | mysticete | 0.298 |
| 51 | *Janjucetus hunderi* NMV P216929 | Mammalodontidae | mysticete | 0.295 |
| 52 | *Cotylocara macei* CCNHM 101 | Xenorophidae | odontocete | 0.293 |
| 53 | *Kentriodon* sp NN | Kentriodontidae | odontocete | 0.293 |
| 54 | *Cephalorhynchus hectori maui* NMNZ MM002607 | Delphinidae | odontocete | 0.291 |
| 55 | *Squalodon bariensis* IRSNB 2372 | Squalodontidae | odontocete | 0.288 |
| 56 | *Berardius bairdii* NHM 1954.9.21.1 | Ziphiidae | odontocete | 0.285 |
| 57 | *Odobenocetops peruvianus* SMNK PAL 2491 | Odobenocetopsidae | odontocete | 0.284 |
| 58 | *Phocoena dioptrica* NHM 1939.9.30.1 | Phocoenidae | odontocete | 0.284 |
| 59 | *Orcinus orca* USNM 11980 | Delphinidae | odontocete | 0.284 |
| 60 | *Lagenorhynchus acutus* USNM 504196 (Leucopleurus) | Delphinidae | odontocete | 0.282 |
| 61 | *Sotalia guianensis* USNM 571558 | Delphinidae | odontocete | 0.281 |
| 62 | *Tursiops aduncus* NHM 1882.1.2.3 | Delphinidae | odontocete | 0.279 |
| 63 | *Cephalorhynchus hectori* NMNZ MM002288 | Delphinidae | odontocete | 0.279 |
| 64 | *Zarhinocetus donnamatsonae* UCMP 86139 | Allodelphinidae | odontocete | 0.279 |
| 65 | *Cephalorhynchus commersonii* USNM 252568 | Delphinidae | odontocete | 0.271 |
| 66 | *Xenorophus* new sp Yap CCNHM 168 | Xenorophidae | odontocete | 0.271 |
| 67 | *Hyperoodon planifrons* NHM 1952.9.30.1 | Ziphiidae | odontocete | 0.271 |
| 68 | *Mesoplodon peruvianus* USNM 571258 | Ziphiidae | odontocete | 0.269 |
| 69 | *Tursiops truncatus* SDNHM 23798 | Delphinidae | odontocete | 0.268 |
| 70 | *Phocoenoides dalli* USNM 276062 | Phocoenidae | odontocete | 0.268 |
| 71 | *Papahu taitapu* OU 22066 | Waipatiidae | odontocete | 0.267 |
| 72 | *Delphinus delphis* AMNH 75332 | Delphinidae | odontocete | 0.267 |
| 73 | *Ambulocetus natans* MSNUP I-16826 | Ambulocetidae | archaeocete | 0.266 |
| 74 | *Cephalorhynchus heavisidii* NHM 1948.7.27.1 | Delphinidae | odontocete | 0.266 |
| 75 | *Waipatia maerewhenua* OU 22095 | Waipatiidae | odontocete | 0.263 |
| 76 | *Prosqualodon davidis* USNM 467596 | Prosqualodontidae | odontocete | 0.260 |
| 77 | Eurhinodelphinidae UCMP 99669 | Eurhinodelphinidae | odontocete | 0.257 |
| 78 | *Ziphius cavirostris* NHM 2006.15 | Ziphiidae | odontocete | 0.257 |
| 79 | *Aglaocetus patulus* USNM 23690 | Aglaocetidae | mysticete | 0.256 |
| 80 | *Lipotes vexillifer* AMNH 57333 | Lipotidae | odontocete | 0.255 |
| 81 | *Semirostrum cerutti* SDNHM 65276 | Phocoenidae | odontocete | 0.254 |
| 82 | *Berardius arnuxii* NHM 1935.10.23.1 | Ziphiidae | odontocete | 0.254 |
| 83 | *Lissodelphis borealis* USNM 550188 | Delphinidae | odontocete | 0.253 |
| 84 | *Mesoplodon hectori* NHM 1949.8.19.1 | Ziphiidae | odontocete | 0.252 |
| 85 | *Steno bredanensis* USNM 572789 | Delphinidae | odontocete | 0.250 |
| 86 | *Artiocetus clavis* GSP-UM 3458 | Protocetidae | archaeocete | 0.249 |
| 87 | *Balaenula astensis* MSNUP I-12555 | Balaenidae | mysticete | 0.249 |
| 88 | *Eurhinodelphis longirostris* USNM 244404 | Eurhinodelphinidae | odontocete | 0.249 |
| 89 | *Aegyptocetus tarfa* MSNTUP I-15459 | Protocetidae | archaeocete | 0.247 |
| 90 | *Lagenorhynchus obscurus* NHMUK 1846.3.11.8 (Sagmatias) | Delphinidae | odontocete | 0.247 |
| 91 | *Lagenorhynchus obliquidens* NHM 1992.83 | Delphinidae | odontocete | 0.245 |
| 92 | *Mesoplodon bowdoini* NMNZ MM001900 | Ziphiidae | odontocete | 0.244 |
| 93 | *Mesoplodon traversii* (juvenile) NMNZ TMP012996 | Ziphiidae | odontocete | 0.242 |
| 94 | Agorophiid USNM 205491 | Agorophiidae | odontocete | 0.242 |
| 95 | *Sousa teuszii* NHM 1992.138 | Delphinidae | odontocete | 0.241 |
| 96 | *Herpetocetus bramblei* UCMP 219111 | Cetotheriidae | mysticete | 0.241 |
| 97 | *Piscolithax longirostris* SAS 933 | Phocoenidae | odontocete | 0.240 |
| 98 | *Mesoplodon layardii* USNM 550150 | Ziphiidae | odontocete | 0.240 |
| 99 | *Pakicetus attocki* PV M 100148 | Pakicetidae | archaeocete | 0.238 |
| 100 | *Balaenoptera floridana* USNM 529244 | Balaenopteridae | mysticete | 0.238 |
| 101 | *Stenella attenuata* NHM 1966.11.18.5 | Delphinidae | odontocete | 0.238 |
| 102 | *Aetiocetus weltoni* UCMP 122900 | Aetiocetidae | mysticete | 0.236 |
| 103 | *Berardius minimus* USNM 276366 | Ziphiidae | odontocete | 0.234 |
| 104 | *Mesoplodon carlhubbsi* USNM 504128 | Ziphiidae | odontocete | 0.232 |
| 105 | *Zarhinocetus errabundus* LACM 149588 | Allodelphinidae | odontocete | 0.232 |
| 106 | *Sousa chinensis* NHM 1992.97 | Delphinidae | odontocete | 0.232 |
| 107 | *Sousa sahulensis* NHM 1992.92 | Delphinidae | odontocete | 0.231 |
| 108 | *Xiphiacetus cristatus* USNM 21363 | Eurhinodelphinidae | odontocete | 0.229 |
| 109 | *Chonecetus goedertorum* LACM 131146 (Fucaia) | Aetiocetidae | mysticete | 0.229 |
| 110 | *Schizodelphis* sp CCNHM 141 | Eurhinodelphinidae | odontocete | 0.229 |
| 111 | *Sousa plumbea* USNM 550941 | Delphinidae | odontocete | 0.228 |
| 112 | *Xiphiacetus bossi* USNM 8842 | Eurhinodelphinidae | odontocete | 0.226 |
| 113 | *Etruridelphis* sp MGPT PU 13884 | Delphinidae | odontocete | 0.226 |
| 114 | *Patriocetus ehrlichii* 1999-3 Cet. 4 | Patriocetidae | odontocete | 0.225 |
| 115 | *Herpetocetus sendaicus* NMNS-PV 19540 | Cetotheriidae | mysticete | 0.225 |
| 116 | *Tasmacetus shepherdi* USNM 484878 | Ziphiidae | odontocete | 0.224 |
| 117 | *Simocetus rayi* USNM 256517 | Simocetidae | odontocete | 0.223 |
| 118 | *Tiucetus rosae* MNHN.F. PPI261 | Cetotheriidae | mysticete | 0.222 |
| 119 | *Parietobalaena palmeri* USNM 24883 | Pelocetidae | mysticete | 0.216 |
| 120 | *Mesoplodon bidens* USNM 593438 | Ziphiidae | odontocete | 0.216 |
| 121 | *Phocoena* *phocoena* AMNH 212161 | Phocoenidae | odontocete | 0.214 |
| 122 | *Mesoplodon hotaula* USNM 593426 | Ziphiidae | odontocete | 0.213 |
| 123 | Patriocetid new genus ChM PV4753 | Patriocetidae | odontocete | 0.212 |
| 124 | *Delphinus capensis* NHM 1981.7.11 | Delphinidae | odontocete | 0.211 |
| 125 | *Mesoplodon europaeus* USNM 571665 | Ziphiidae | odontocete | 0.211 |
| 126 | *Echovenator sandersi* GSM 1098 | Xenorophidae | odontocete | 0.208 |
| 127 | *Mesoplodon grayi* USNM 49880 | Ziphiidae | odontocete | 0.207 |
| 128 | *Pliopontos littoralis* SAS 193 | Pontoporiidae | odontocete | 0.207 |
| 129 | *Capricornis sumatrensis* NHM 24.5.29.1 | Bovidae | terrestrial artiodactyl | 0.205 |
| 130 | *Argyrocetus joaquinensis* USNM 11996 | Unclear | odontocete | 0.205 |
| 131 | *Mesoplodon stejnegeri* USNM 504330 | Ziphiidae | odontocete | 0.204 |
| 132 | *Schizodelphis barnesi* MNHN AMN 19 | Eurhinodelphinidae | odontocete | 0.203 |
| 133 | *Mesoplodon mirus* USNM 504612 | Ziphiidae | odontocete | 0.202 |
| 134 | *Atocetus iquensis* MNHN.F.PPI. 113 | Delphinida | odontocete | 0.202 |
| 135 | *Balaenoptera* sp SDNHM 83695 | Balaenopteridae | mysticete | 0.201 |
| 136 | *Inia geoffrensis* AMNH 93415 | Iniidae | odontocete | 0.201 |
| 137 | *Diorocetus hiatus* USNM 16783 | Pelocetidae | mysticete | 0.200 |
| 138 | *Miocaperea pulchra* SMNS 46978 | Cetotheriidae | mysticete | 0.196 |
| 139 | *Bos* sp NHM 1981.984 | Bovidae | terrestrial artiodactyl | 0.195 |
| 140 | *Aetiocetus cotylalveus* USNM 25210 | Aetiocetidae | mysticete | 0.193 |
| 141 | *Mesoplodon ginkgodens* USNM 298237 | Ziphiidae | odontocete | 0.193 |
| 142 | *Choeropsis liberiensis* NHM 1967.3.20.1 | Hippopotamidae | terrestrial artiodactyl | 0.189 |
| 143 | *Parapontoporia sternbergi* SDNHM 75060 | Lipotidae | odontocete | 0.189 |
| 144 | *Patriocetus* sp MB Ma. 42882 | Patriocetidae | odontocete | 0.188 |
| 145 | *Tragulus kanchil* NHM 9.1.5.850 | Tragulidae | terrestrial artiodactyl | 0.187 |
| 146 | *Balaenoptera musculus* NHM 1892.3.1.1 | Balaenopteridae | mysticete | 0.184 |
| 147 | *Xenorophus* new sp ChM PV4823 | Xenorophidae | odontocete | 0.183 |
| 148 | *Schizodelphis morckhoviensis* USNM 13873 | Eurhinodelphinidae | odontocete | 0.183 |
| 149 | *Pontoporia blainvillei* USNM 482727 | Pontoporiidae | odontocete | 0.179 |
| 150 | *Cervus elaphus* NHM 2005.16 | Cervidae | terrestrial artiodactyl | 0.177 |
| 151 | *Saiga tatarica* NHM 1961.5.30.1 | Bovidae | terrestrial artiodactyl | 0.176 |
| 152 | *Piscobalaena nana* MNHN 1618 | Cetotheriidae | mysticete | 0.169 |
| 153 | *Eubalaena australis* NHM 1873.3.3 | Balaenidae | mysticete | 0.169 |
| 154 | *Megaptera novaeangliae* GERM.792a | Balaenopteridae | mysticete | 0.168 |
| 155 | *Eubalaena glacialis* MSNUP NN | Balaenidae | mysticete | 0.164 |
| 156 | *Mixocetus* sp LACM 143474 | Tranatocetidae | mysticete | 0.163 |
| 157 | *Kekenodon* OU 22294 | Kekenodontidae | archaeocete | 0.160 |
| 158 | *Coronodon havensteini* CCNHM 108 | Aetiocetidae | mysticete | 0.158 |
| 159 | *Hydropotes inemis* NHM 1551c | Cervidae | terrestrial artiodactyl | 0.157 |
| 160 | *Tayassu pecari labiatus* NHM 47.4.6.8 | Tayassuidae | terrestrial artiodactyl | 0.154 |
| 161 | *Giraffa camelopardalis* NHM NN | Giraffidae | terrestrial artiodactyl | 0.151 |
| 162 | *Caperea marginata* NHM 1876.2.16.1 | Cetotheriidae | mysticete | 0.145 |
| 163 | *Balaena mysticetus* 1986.1.16 | Balaenidae | mysticete | 0.143 |
| 164 | *Pelocetus calvertensis* USNM 11976 | Pelocetidae | mysticete | 0.137 |
| 165 | *Remingtonocetus harudiensis* USNM PAL 559313 | Remingtonocetidae | archaeocete | 0.135 |
| 166 | *Balaenoptera acutorostrata* NHM 1965.11.2.1 | Balaenopteridae | mysticete | 0.128 |
| 167 | *Balaenoptera omurai* NN | Balaenopteridae | mysticete | 0.124 |
| 168 | *Camelus dromedarius* NHM NN | Camelidae | terrestrial artiodactyl | 0.121 |
| 169 | *Balaenoptera edeni* NHM 1920.12.31.1 | Balaenopteridae | mysticete | 0.120 |
| 170 | *Balaenoptera borealis* NHM 1934.5.25.1 | Balaenopteridae | mysticete | 0.119 |
| 171 | *Balaenoptera physalus* NHM 1862.2.7.181 | Balaenopteridae | mysticete | 0.119 |
| 172 | *Balaenoptera brydei* USNM 572922 | Balaenopteridae | mysticete | 0.115 |

**Removal of fossils**

The landmarks of highest variation change slightly when the fossils are removed. Firstly, in the mysticetes, the average amount of total cetacean cranial variation decreases to Σ*p* = 0.142 (with fossils: Σ*p =*0.191) when only the extant mysticete skulls are analysed. This is likely because there is a higher amount of deformation in the rostrums of fossil mysticetes. When the fossils are removed there is a slight reordering in the landmarks of variation, including less asymmetry in the rostrum, and a low level of asymmetry in the orbit, squamosal, and parietal. In the odontocetes there is very little change in the top 10 landmarks of variation when the fossils are removed. The top landmarks of variation for all odontocetes combined are the nasal, frontal, and posterior premaxilla and maxilla, this does not change considerably when the fossils are removed. This is likely due to the odontocete signal being dominated by extant taxa. The average total skull variation in the extant odontocetes increased marginally when the fossils were removed: Σ*p =*0.292 (with fossils Σ*p =* 0.290). This is probably due to fossil odontocetes having more symmetrical crania compared to extant odontocetes.

**Exclusion of the rostrum**

In the archaeocetes, 4 of the top 5 landmarks of variation are found in the rostrum. In the mysticetes, the rostrum, and landmarks on the anterior lateral ventral of the maxilla, and the premaxilla ventral midline posterior suture (both located on the rostrum) make up 6 of the 10 top highest landmarks of variation. In odontocetes, rostral landmarks accounted for 2 of the 10 highest landmarks of variation, and therefore removing them had the lowest impact for this group.  These models were designed as sensitivity analyses to assess the impact of the rostrum on the overall skull asymmetry. Excluding the rostral landmarks had no effect on the ordering of the model fits), i.e., the ranking of the models by AIC weights **(Table S5b** - Akaike information criterion (AIC) rankings for each evolutionary model for asymmetry in the cetacean cranium) nor on the trait **(Fig. S1** - Asymmetry in the cetacean skull with rostrum removed). Therefore, we ran all our remaining analyses on the whole dataset with the rostrum included. Rostral landmarks were landmarks: 5, 6, 8, 9, 62, 63, 64, 65, 66, 69, 70, 72, 73, 115, 116, 117, 118, 119 (see **Table S11** -123 landmarks added to the entire surface of the skull) for details.

Table S2. Percentage of asymmetry in the rostrum – archaeocetes. The sum radii of the archaeocete skull, the sum radii of the archaeocete skull with the rostral landmarks removed, and the percentage (%) of the sum radii that is found in the rostrum of archaeocetes

| **Species** | **Family** | **Sum radii with rostrum** | **Sum radii with rostrum removed** | **% of asymmetry in rostrum** |
| --- | --- | --- | --- | --- |
| *Aegyptocetus tarfa* MSNTUP I-15459 | Protocetidae | 0.247 | 0.213 | 13.8 |
| *Ambulocetus natans* MSNUP I-16826 | Ambulocetidae | 0.266 | 0.227 | 14.8 |
| *Artiocetus clavis* GSP-UM 3458 | Protocetidae | 0.249 | 0.171 | 31.3 |
| *Basilosaurus isis* SMNS 11787 | Basilosauridae | 0.308 | 0.257 | 16.4 |
| *Dorudon atrox* PV M 100149 | Basilosauridae | 0.298 | 0.244 | 18.1 |
| *Kekenodon sp* OU 22294 | Kekenodontidae | 0.160 | 0.137 | 14.5 |
| *Pakicetus attocki* PV M 100148 | Pakicetidae | 0.238 | 0.159 | 33.2 |
| *Protocetus atavus* SMNS 11084 | Protocetidae | 0.298 | 0.247 | 17.1 |
| *Remingtonocetus harudiensis* USNM PAL 559313 | Remingtonocetidae | 0.135 | 0.111 | 18.1 |
| *Zygorhiza kochii* USNM 11962 | Basilosauridae | 0.306 | 0.258 | 15.8 |

Table S3. Percentage of asymmetry in the rostrum – mysticetes. The sum radii of the mysticete skull, the sum radii of the mysticete skull with the rostral landmarks removed, and the percentage (%) of the sum radii that is found in the rostrum of mysticetes

| **Species** | **Family** | **Sum radii with rostrum** | **Sum radii with rostrum removed** | **% of asymmetry in rostrum** |
| --- | --- | --- | --- | --- |
| *Aetiocetus cotylalveus* USNM 25210 | Aetiocetidae | 0.193 | 0.158 | 18.0 |
| *Aetiocetus weltoni* UCMP 122900 | Aetiocetidae | 0.236 | 0.215 | 8.7 |
| *Aglaocetus moreni* FMNH P13407 | Aglaocetidae | 0.298 | 0.262 | 11.8 |
| *Aglaocetus patulus* USNM 23690 | Aglaocetidae | 0.256 | 0.216 | 15.7 |
| *Balaena mysticetus* NHMUK 1986.1.16 | Balaenidae | 0.143 | 0.127 | 11.4 |
| *Balaenoptera acutorostrata* NHM 1965.11.2.1 | Balaenopteridae | 0.128 | 0.114 | 10.4 |
| *Balaenoptera borealis* NHMUK 1934.5.25.1 | Balaenopteridae | 0.119 | 0.103 | 13.8 |
| *Balaenoptera brydei* USNM 572922 | Balaenopteridae | 0.115 | 0.101 | 11.8 |
| *Balaenoptera sp* SDNHM 83695 | Balaenopteridae | 0.201 | 0.178 | 11.1 |
| *Balaenoptera edeni* NHMUK 1920.12.31.1 | Balaenopteridae | 0.120 | 0.104 | 14.0 |
| *Balaenoptera floridana* USNM 529244 | Balaenopteridae | 0.238 | 0.210 | 11.9 |
| *Balaenoptera musculus* NHMUK 1892.3.1.1 | Balaenopteridae | 0.184 | 0.159 | 13.8 |
| *Balaenoptera omurai* NN | Balaenopteridae | 0.124 | 0.108 | 12.6 |
| *Balaenoptera physalus* NHMUK 1862.2.7.181 | Balaenopteridae | 0.119 | 0.100 | 16.2 |
| Balaenopteridae NMNZ MM001630 | Balaenopteridae | 0.300 | 0.263 | 12.4 |
| *Balaenula astensis* MSNUP I-12555 | Balaenidae | 0.249 | 0.210 | 15.6 |
| *Caperea marginata* NHMUK 1876.2.16.1 | Cetotheriidae | 0.145 | 0.122 | 16.1 |
| *Chonecetus goedertorum* LACM 131146 (Fucaia) | Aetiocetidae | 0.229 | 0.189 | 17.6 |
| *Coronodon havensteini* CCNHM 108 | Aetiocetidae | 0.158 | 0.137 | 13.1 |
| *Diorocetus hiatus* USNM 16783 | Pelocetidae | 0.200 | 0.170 | 15.2 |
| *Eubalaena australis* NHM 1873.3.3 | Balaenidae | 0.169 | 0.147 | 13.0 |
| *Eubalaena glacialis* MSNUP NN | Balaenidae | 0.164 | 0.140 | 14.6 |
| *Herpetocetus bramblei* UCMP 219111 | Cetotheriidae | 0.241 | 0.183 | 24.3 |
| *Herpetocetus sendaicus* NMNS-PV 19540 | Cetotheriidae | 0.225 | 0.184 | 18.0 |
| *Janjucetus hunderi* NMV P216929 | Mammalodontidae | 0.295 | 0.252 | 14.8 |
| *Megaptera novaeangliae* GERM.792a | Balaenopteridae | 0.168 | 0.146 | 13.2 |
| *Miocaperea pulchra* SMNS 46978 | Cetotheriidae | 0.196 | 0.173 | 12.0 |
| *Mixocetus sp* LACM 143474 | Tranatocetidae | 0.163 | 0.140 | 14.2 |
| *Parietobalaena palmeri* USNM 24883 | Pelocetidae | 0.216 | 0.185 | 14.5 |
| *Pelocetus calvertensis* USNM 11976 | Pelocetidae | 0.137 | 0.118 | 13.5 |
| *Piscobalaena nana* MNHN 1618 | Cetotheriidae | 0.169 | 0.142 | 16.0 |
| *Tiucetus rosae* MNHN.F. PPI261 | Cetotheriidae | 0.222 | 0.187 | 16.1 |

[Next page]

Table S4. Percentage of asymmetry in the rostrum – odontocetes. The sum radii of the odontocete skull, the sum radii of the odontocete skull with the rostral landmarks removed, and the percentage (%) of the sum radii that is found in the rostrum of odontocetes

| **Species** | **Family** | **Sum radii with rostrum** | **Sum radii with rostrum removed** | **% of asymmetry in rostrum** |
| --- | --- | --- | --- | --- |
| Agorophiid USNM 205491 | Agorophiidae | 0.242 | 0.209 | 13.8 |
| *Albertocetus* ChM PV8680 | Xenorophidae | 0.394 | 0.304 | 22.7 |
| *Albireo whistleri* UCR 14589 | Albireonidae | 0.393 | 0.346 | 11.8 |
| *Argyrocetus joaquinensis* USNM 11996 | Unclear | 0.205 | 0.178 | 13.1 |
| *Atocetus iquensis* MNHN.F.PPI. 113 | Delphinida | 0.202 | 0.186 | 8.2 |
| *Aulophyseter morricei* UCMP 81661 | Physeteridae | 0.489 | 0.420 | 14.2 |
| *Berardius arnuxii* NHM 1935.10.23.1 | Ziphiidae | 0.254 | 0.198 | 22.0 |
| *Berardius bairdii* NHM 1954.9.21.1 | Ziphiidae | 0.285 | 0.231 | 19.0 |
| *Berardius minimus* USNM 276366 | Ziphiidae | 0.234 | 0.191 | 18.2 |
| *Brachydelphis mazeasi* MNHN.F.PPI. 266 | Pontoporiidae | 0.382 | 0.317 | 17.0 |
| *Cephalorhynchus commersonii* USNM 252568 | Delphinidae | 0.271 | 0.237 | 12.7 |
| *Cephalorhynchus eutropia* NHM 1881.8.17.1 | Delphinidae | 0.321 | 0.272 | 15.4 |
| *Cephalorhynchus heavisidii* NHM 1948.7.27.1 | Delphinidae | 0.266 | 0.233 | 12.3 |
| *Cephalorhynchus hectori maui* NMNZ MM002607 | Delphinidae | 0.291 | 0.253 | 13.1 |
| *Cephalorhynchus hectori* NMNZ MM002288 | Delphinidae | 0.279 | 0.243 | 12.9 |
| *Cotylocara macei* CCNHM 101 | Xenorophidae | 0.293 | 0.237 | 19.1 |
| *Delphinapterus leucas* USNM 305071 | Monodontidae | 0.453 | 0.390 | 14.0 |
| *Delphinus capensis* NHM 1981.7.11 | Delphinidae | 0.211 | 0.180 | 14.8 |
| *Delphinus delphis* AMNH 75332 | Delphinidae | 0.267 | 0.222 | 16.9 |
| *Echovenator sandersi* GSM 1098 | Xenorophidae | 0.208 | 0.178 | 14.1 |
| *Etruridelphis* sp MGPT PU 13884 | Delphinidae | 0.226 | 0.207 | 8.5 |
| Eurhinodelphinidae UCMP 99669 | Eurhinodelphinidae | 0.257 | 0.231 | 10.1 |
| *Eurhinodelphis* longirostris USNM 244404 | Eurhinodelphinidae | 0.249 | 0.187 | 25.0 |
| *Feresa attenuata* USNM 504916 | Delphinidae | 0.369 | 0.318 | 13.9 |
| *Globicephala macrorhynchus* NHM 1912.10.27 | Delphinidae | 0.407 | 0.332 | 18.4 |
| *Globicephala melas* NMNZ MM001946 | Delphinidae | 0.410 | 0.370 | 9.7 |
| *Globicephala* sp USNM 21867 | Delphinidae | 0.324 | 0.274 | 15.5 |
| *Grampus griseus* USNM 571602 | Delphinidae | 0.343 | 0.298 | 13.3 |
| *Hemisyntrachelus cortesii* MBGPT NN | Delphinidae | 0.354 | 0.299 | 15.5 |
| *Hyperoodon ampullatus* NHM 1992.42 | Ziphiidae | 0.308 | 0.262 | 14.9 |
| *Hyperoodon planifrons* NHM 1952.9.30.1 | Ziphiidae | 0.271 | 0.228 | 15.9 |
| *Indopacetus pacificus* USNM 593534 | Ziphiidae | 0.363 | 0.288 | 20.6 |
| *Inia geoffrensis* AMNH 93415 | Iniidae | 0.201 | 0.168 | 16.2 |
| *Kampholophus serrulus* UMCP 36045 | Kentriodontidae | 0.357 | 0.312 | 12.6 |
| *Kentriodon pernix* USNM 10670 | Kentriodontidae | 0.320 | 0.275 | 14.3 |
| *Kentriodon* sp NN | Kentriodontidae | 0.293 | 0.227 | 22.5 |
| *Kogia breviceps* USNM 22015 | Kogiidae | 0.462 | 0.427 | 7.8 |
| *Kogia simus* NHM.1952.8.28.1 | Kogiidae | 0.457 | 0.416 | 9.0 |
| *Lagenodelphis hosei* USNM 571619 | Delphinidae | 0.328 | 0.280 | 14.5 |
| *Lagenorhynchus acutus* USNM 504196 (Leucopleurus) | Delphinidae | 0.282 | 0.232 | 17.5 |
| *Lagenorhynchus albirostris* AMNH 37162 | Delphinidae | 0.355 | 0.296 | 16.8 |
| *Lagenorhynchus australis* 1944.11.30.1 (Sagmatias) | Delphinidae | 0.309 | 0.278 | 10.0 |
| *Lagenorhynchus cruciger* NHMUK 1960.8.24.1  (Sagmatias) | Delphinidae | 0.368 | 0.316 | 14.1 |
| *Lagenorhynchus obliquidens* NHM 1992.83 | Delphinidae | 0.245 | 0.220 | 10.1 |
| *Lagenorhynchus obscurus* NHMUK 1846.3.11.8  (Sagmatias) | Delphinidae | 0.247 | 0.217 | 12.0 |
| *Lipotes vexillifer* AMNH 57333 | Lipotidae | 0.255 | 0.211 | 17.3 |
| *Lissodelphis borealis* USNM 550188 | Delphinidae | 0.253 | 0.220 | 12.9 |
| *Lissodelphis peronii* NMNZ MM002116 | Delphinidae | 0.304 | 0.253 | 16.6 |
| *Lomacetus ginsburgi* MNHN.F.PPI.104 | Phocoenidae | 0.322 | 0.269 | 16.3 |
| *Mesoplodon bidens* USNM 593438 | Ziphiidae | 0.216 | 0.188 | 13.0 |
| *Mesoplodon bowdoini* NMNZ MM001900 | Ziphiidae | 0.244 | 0.200 | 18.2 |
| *Mesoplodon carlhubbsi* USNM 504128 | Ziphiidae | 0.232 | 0.197 | 15.2 |
| *Mesoplodon europaeus* USNM 571665 | Ziphiidae | 0.211 | 0.173 | 17.8 |
| *Mesoplodon ginkgodens* USNM 298237 | Ziphiidae | 0.193 | 0.161 | 16.6 |
| *Mesoplodon grayi* USNM 49880 | Ziphiidae | 0.207 | 0.172 | 17.0 |
| *Mesoplodon hectori* NHM 1949.8.19.1 | Ziphiidae | 0.252 | 0.211 | 16.4 |
| *Mesoplodon hotaula* USNM 593426 | Ziphiidae | 0.213 | 0.187 | 12.1 |
| *Mesoplodon layardii* USNM 550150 | Ziphiidae | 0.240 | 0.196 | 18.5 |
| *Mesoplodon mirus* USNM 504612 | Ziphiidae | 0.202 | 0.179 | 11.6 |
| *Mesoplodon peruvianus* USNM 571258 | Ziphiidae | 0.269 | 0.229 | 14.8 |
| *Mesoplodon stejnegeri* USNM 504330 | Ziphiidae | 0.204 | 0.174 | 14.6 |
| *Mesoplodon traversii* (juvenile) NMNZ TMP012996 | Ziphiidae | 0.242 | 0.192 | 20.6 |
| *Messapicetus longirostris* MSNUP NN | Ziphiidae | 0.327 | 0.298 | 9.0 |
| *Monodon monoceros* USNM 267959 | Monodontidae | 0.546 | 0.472 | 13.4 |
| *Neophocaena asiaeorientalis* USNM 240001 | Phocoenidae | 0.323 | 0.281 | 12.9 |
| *Neophocaena phocaenoides* NHM 1903.9.12.3 | Phocoenidae | 0.328 | 0.295 | 10.3 |
| *Notocetus vanbenedeni* MLP 55 | Squalodelphinidae | 0.404 | 0.338 | 16.4 |
| *Odobenocetops peruvianus* SMNK PAL 2491 | Odobenocetopsidae | 0.284 | 0.237 | 16.5 |
| *Orcaella brevirostris* NHM.1883.11.20.2 | Delphinidae | 0.374 | 0.321 | 14.3 |
| *Orcaella heinsohni* USNM 284430 | Delphinidae | 0.350 | 0.301 | 14.1 |
| *Orcinus orca* USNM 11980 | Delphinidae | 0.284 | 0.250 | 12.1 |
| *Orycterocetus crocodilinus* USNM 22926 | Physeteridae | 0.518 | 0.456 | 11.9 |
| *Papahu taitapu* OU 22066 | Waipatiidae | 0.267 | 0.236 | 11.7 |
| *Parapontoporia sternbergi* SDNHM 75060 | Lipotidae | 0.189 | 0.156 | 17.5 |
| Patriocetid new genus ChM PV4753 | Patriocetidae | 0.212 | 0.192 | 9.3 |
| *Patriocetus ehrlichii* 1999-3 Cet. 4 | Patriocetidae | 0.225 | 0.193 | 14.1 |
| *Patriocetus* sp MB Ma. 42882 | Patriocetidae | 0.188 | 0.166 | 12.0 |
| *Peponocephala electra* USNM 504511 | Delphinidae | 0.322 | 0.276 | 14.4 |
| *Phocoena dioptrica* NHM 1939.9.30.1 | Phocoenidae | 0.284 | 0.249 | 12.4 |
| *Phocoena phocoena* AMNH 212161 | Phocoenidae | 0.214 | 0.182 | 15.0 |
| *Phocoena sinus* SDNHM 20697 | Phocoenidae | 0.309 | 0.266 | 14.1 |
| *Phocoena spinipinnis* NHM 1900.5.7.29 | Phocoenidae | 0.320 | 0.278 | 12.9 |
| *Phocoenoides dalli* USNM 276062 | Phocoenidae | 0.268 | 0.221 | 17.6 |
| *Physeter macrocephalus* NHM 2007.1 | Physeteridae | 0.456 | 0.381 | 16.5 |
| *Piscolithax longirostris* SAS 933 | Phocoenidae | 0.240 | 0.192 | 20.3 |
| *Platanista gangetica* USNM 172409 | Platanistidae | 0.449 | 0.365 | 18.6 |
| *Pliopontos littoralis* SAS 193 | Pontoporiidae | 0.207 | 0.186 | 10.0 |
| *Pontoporia blainvillei* USNM 482727 | Pontoporiidae | 0.179 | 0.163 | 8.9 |
| *Prosqualodon davidis* USNM 467596 | Prosqualodontidae | 0.260 | 0.224 | 13.7 |
| *Pseudorca crassidens* USNM 11320 | Delphinidae | 0.408 | 0.357 | 12.6 |
| *Schizodelphis barnesi* MNHN AMN 19 | Eurhinodelphinidae | 0.203 | 0.184 | 9.5 |
| *Schizodelphis morckhoviensis* USNM 13873 | Eurhinodelphinidae | 0.183 | 0.150 | 18.0 |
| *Schizodelphis* sp CCNHM 141 | Eurhinodelphinidae | 0.229 | 0.194 | 15.0 |
| *Semirostrum cerutti* SDNHM 65276 | Phocoenidae | 0.254 | 0.230 | 9.3 |
| *Septemtriocetus bosselaersi* IRSNB M.1928 | Phocoenidae | 0.382 | 0.323 | 15.5 |
| Shark-toothed *squalodon* OU 21798 | Squalodontidae | 0.340 | 0.283 | 16.7 |
| *Simocetus rayi* USNM 256517 | Simocetidae | 0.223 | 0.180 | 19.4 |
| *Sotalia guianensis* USNM 571558 | Delphinidae | 0.281 | 0.237 | 15.6 |
| *Sousa chinensis* NHM 1992.97 | Delphinidae | 0.232 | 0.201 | 13.0 |
| *Sousa plumbea* USNM 550941 | Delphinidae | 0.228 | 0.204 | 10.6 |
| *Sousa sahulensis* NHM 1992.92 | Delphinidae | 0.231 | 0.183 | 20.5 |
| *Sousa teuszii* NHM 1992.138 | Delphinidae | 0.241 | 0.208 | 13.7 |
| *Squalodon bariensis* IRSNB 2372 | Squalodontidae | 0.288 | 0.264 | 8.5 |
| *Squalodon calvertensis* NMNZ MM001996 | Squalodontidae | 0.372 | 0.307 | 17.4 |
| *Stenella attenuata* NHM 1966.11.18.5 | Delphinidae | 0.238 | 0.203 | 14.8 |
| *Stenella longirostris* USNM 395270 | Delphinidae | 0.320 | 0.272 | 15.1 |
| *Steno bredanensis* USNM 572789 | Delphinidae | 0.250 | 0.215 | 14.0 |
| *Tagicetus joneti* IRSNB M. 1892 | Delphinida | 0.406 | 0.344 | 15.4 |
| *Tasmacetus shepherdi* USNM 484878 | Ziphiidae | 0.224 | 0.199 | 10.9 |
| *Tursiops aduncus* NHM 1882.1.2.3 | Delphinidae | 0.279 | 0.231 | 17.3 |
| *Tursiops truncatus* SDNHM 23798 | Delphinidae | 0.268 | 0.227 | 15.1 |
| *Waipatia maerewhenua* OU 22095 | Waipatiidae | 0.263 | 0.204 | 22.5 |
| *Xenorophus* new sp ChM PV4823 | Xenorophidae | 0.183 | 0.153 | 16.4 |
| *Xenorophus* new sp Yap CCNHM 168 | Xenorophidae | 0.271 | 0.218 | 19.6 |
| *Xiphiacetus bossi* USNM 8842 | Eurhinodelphinidae | 0.226 | 0.201 | 10.9 |
| *Xiphiacetus cristatus* USNM 21363 | Eurhinodelphinidae | 0.229 | 0.201 | 12.2 |
| *Zarhachis flagellator* USNM 10911 | Platanistidae | 0.325 | 0.258 | 20.7 |
| *Zarhinocetus donnamatsonae* UCMP 86139 | Allodelphinidae | 0.279 | 0.247 | 11.3 |
| *Zarhinocetus errabundus* LACM 149588 | Allodelphinidae | 0.232 | 0.195 | 16.1 |
| *Ziphius cavirostris* NHM 2006.15 | Ziphiidae | 0.257 | 0.213 | 17.3 |


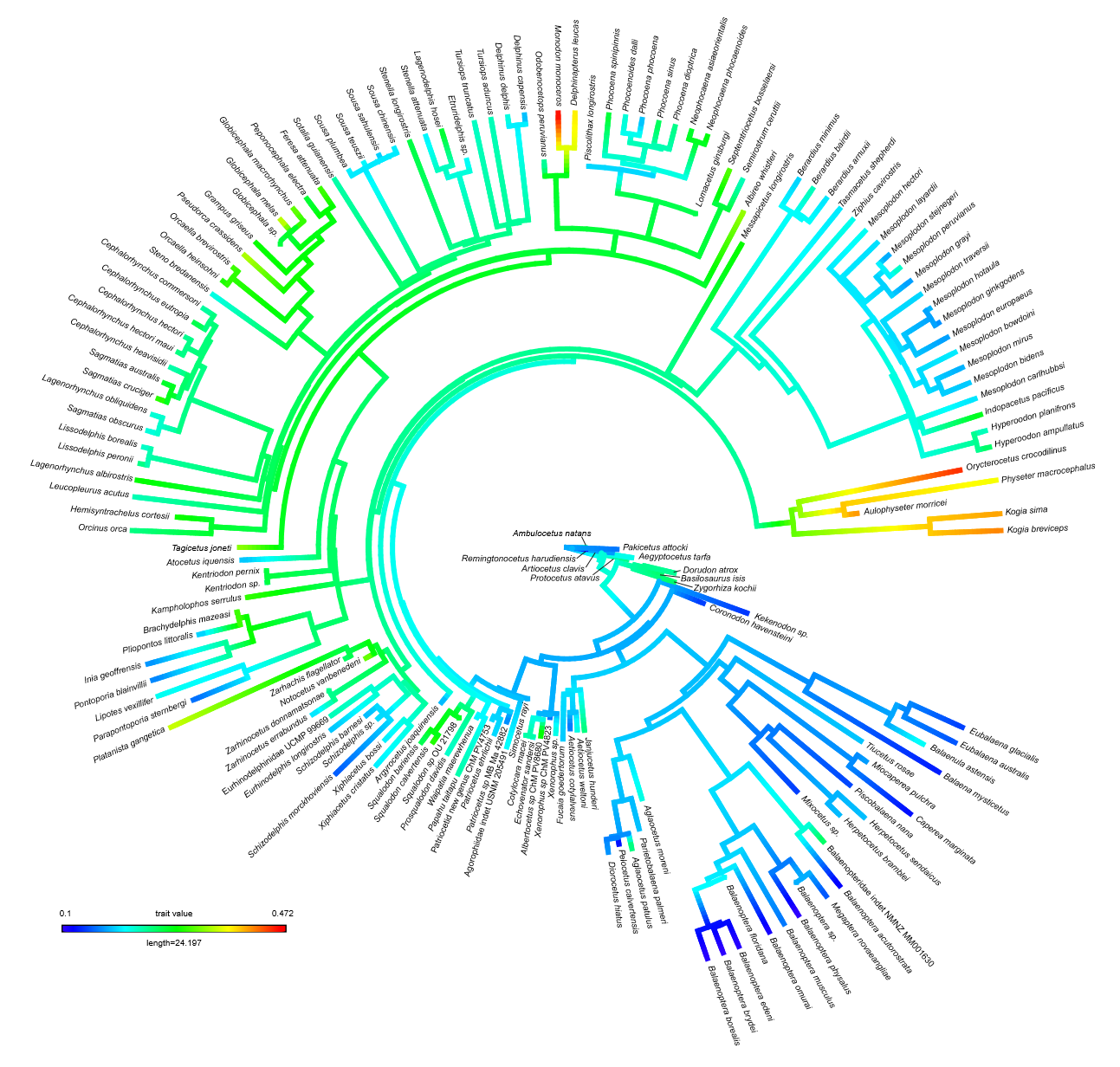


[previous page]

Fig S1. Asymmetry in the cetacean skull with the rostrum removed. Time-calibrated phylogeny adapted from Lloyd and Slater [29] for sampled cetacean species with branches showing the level of asymmetry (∑*p*_spec_) after rostral landmarks have been removed. The trait value is the sum of Euclidean distances between the computer mirrored landmark and the manually placed landmark. The larger the value for Σ*p*_spec_, the more the landmarks have been displaced, indicating asymmetry between the two sides of the cranium.


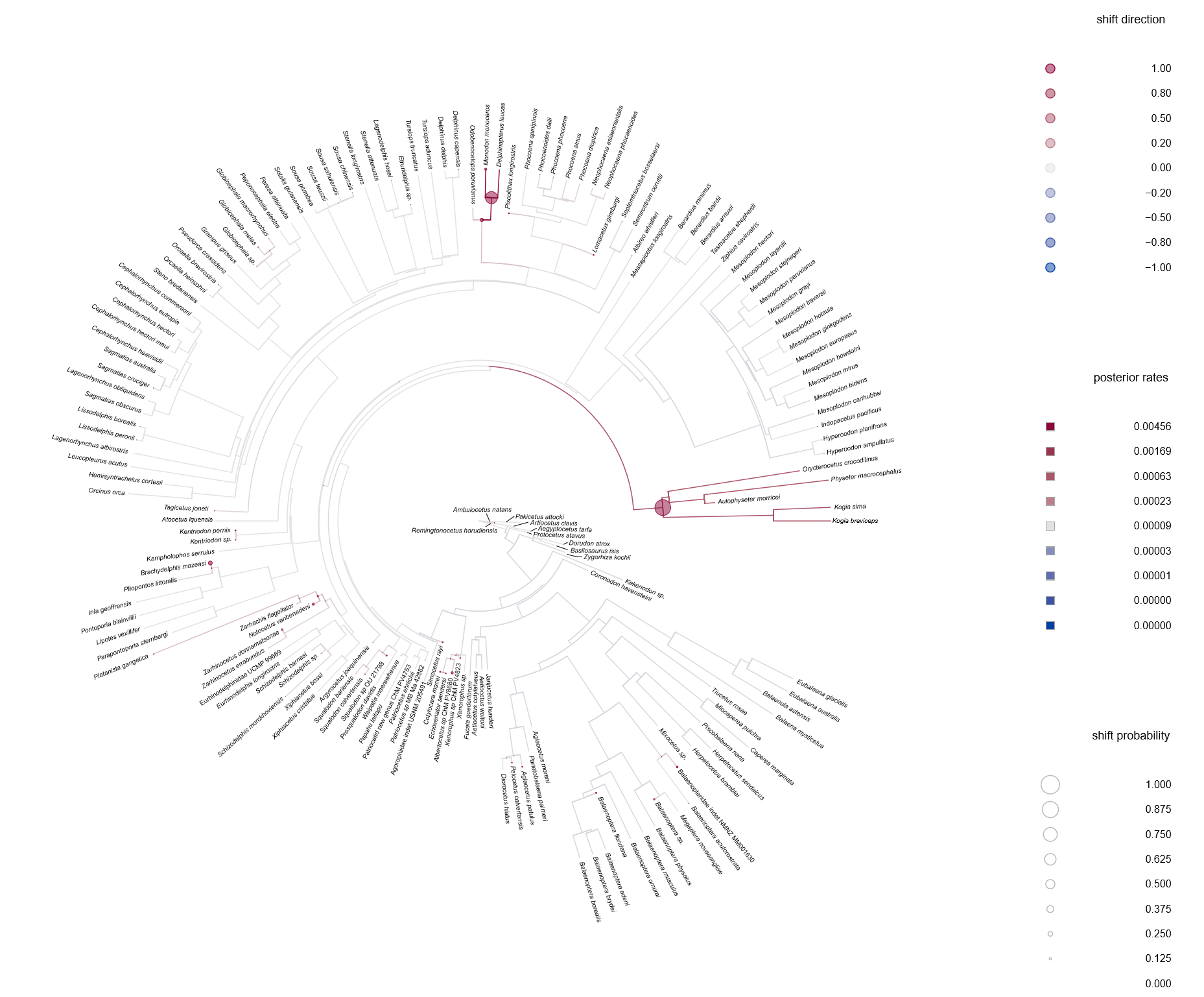


[previous page]

Fig S2. Reconstructed probability of shifts in cetacean cranial asymmetry (∑*p*_spec_) with the rostrum removed. Reconstructed probability along each branch of the phylogeny under the assumption of relaxed Brownian Motion with a Half-Cauchy distribution for the prior density of the rate scalar. Circles indicate a shift in the trait on either the branch or in the whole clade. The colour of the circle indicates the shift direction with red indicating forward shifts and blue indicating backwards shifts. The size of the circle indicates the probability of the shift occurring in that position in the clade with the largest circle (here, 0.875) indicating the highest probability of a shift occurring. The colour of the branch itself indicates posterior rates for that branch with red showing higher, increasing rates and blue showing lower, decreasing rates. The background rate is shown as grey. Phylogeny based on Lloyd and Slater [29].


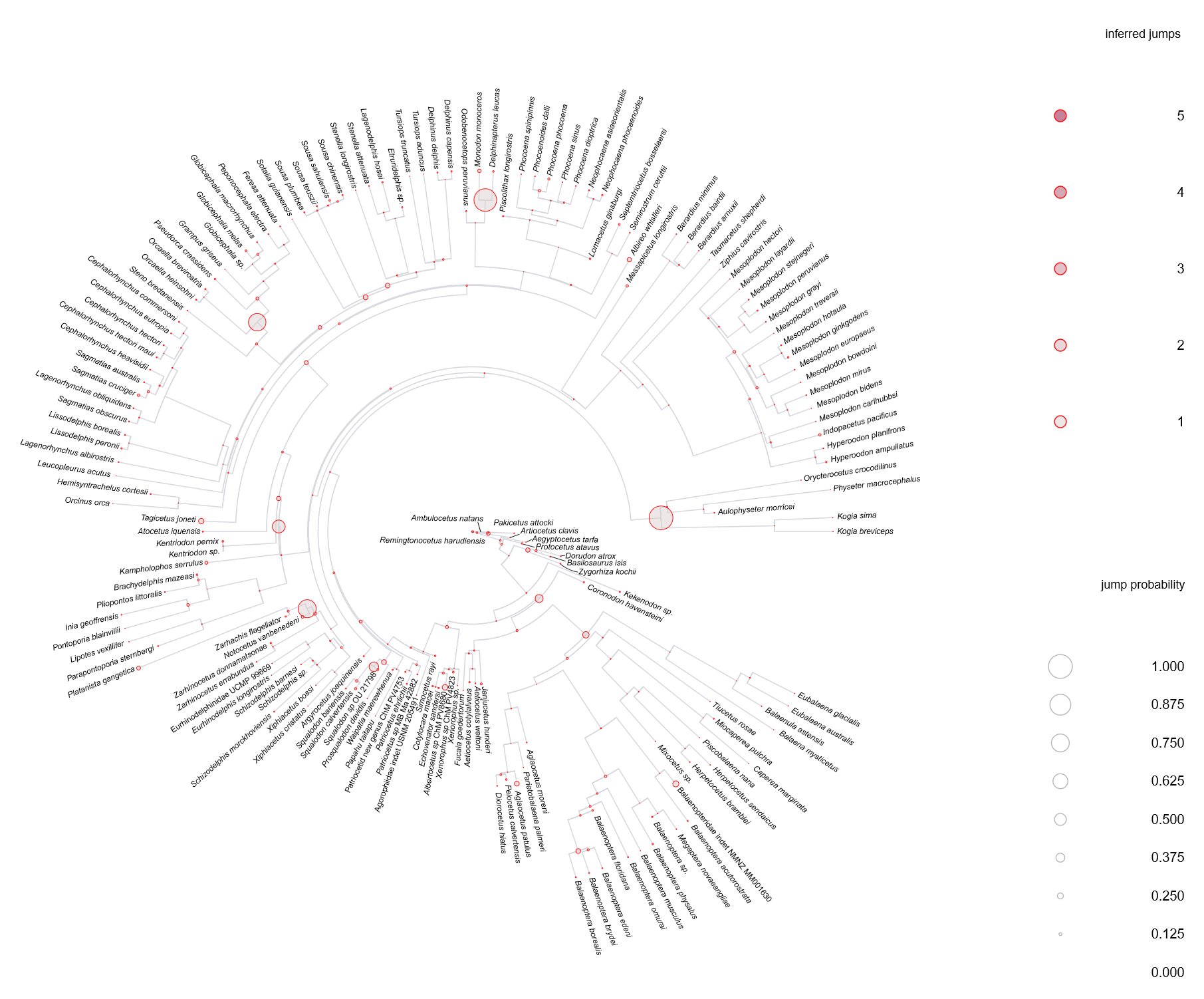


[previous page]

Fig S3. Reconstructed jumps in the rate of cetacean cranial asymmetry (∑*p*_spec_) with the rostrum removed. The model also predicts the number of jumps which may have occurred. The size of the circle indicates the probability of the jump occurring there. The colour of the circle indicates the number of inferred jumps, where dark red = 5 and pale red = 1. Phylogeny based on Lloyd and Slater [29].

Table S5. Akaike information criterion (AIC) rankings for each evolutionary model for asymmetry in the cetacean cranium. The models were a classic Brownian Motion model (BM), a BM model with a selective regime (BMM), a BM model with a selective regime and an independent trend (BMMtr), a BM model with a selective regime which estimates separate phylogenetic means (BMMsm), a classic BM model with *no* selective regime and which estimates separate phylogenetic means (BMsm), a classic Ornstein-Ulenbeck (OU) model, and an OU model with a selective regime (OUM). a) with all landmarks b) with rostral landmarks removed c) with a phylogeny that includes only taxa that appear in a character matrix [29].

a)

| **Model** | **Rank** | **AIC** | **Diff** |
| --- | --- | --- | --- |
| OUM-regime | 1 | -448 | 0.00 |
| OUM-regime-split | 2 | -445 | 3.48 |
| OUM-echo-freq | 3 | -403 | 45.04 |
| OUM-ancestral | 4 | -379 | 69.66 |
| OUM-echo | 5 | -373 | 75.02 |
| BMM-echo | 6 | -372 | 75.99 |
| BMMtr-echo | 7 | -371 | 77.51 |
| BMMsm-echo | 8 | -367 | 81.08 |
| BMMsm-echo-freq | 9 | -343 | 105.82 |
| OU-ancestral | 10 | -342 | 106.55 |
| BMM-echo-freq | 11 | -323 | 124.96 |
| BMMtr-echo-freq | 12 | -322 | 126.13 |
| BMMsm-regime | 13 | -295 | 152.94 |
| BMMsm-regime-split | 14 | -294 | 154.17 |
| BMMtr-ancestral | 15 | -286 | 162.63 |
| BMM-ancestral | 16 | -285 | 163.57 |
| BMM-regime | 17 | -284 | 164.41 |
| BMM-regime-split | 18 | -283 | 165.55 |
| BMMtr-regime | 19 | -282 | 166.33 |
| BMMsm-ancestral | 20 | -282 | 166.71 |
| BMMtr-regime-split | 21 | -280 | 168.23 |
| BM-ancestral | 22 | -275 | 172.87 |
| BMtr-ancestral | 23 | -273 | 175.22 |
| BMsm-ancestral | 24 | -272 | 176.73 |

b)

| Model | Rank | AIC | Diff |
| --- | --- | --- | --- |
| OUM-regime | 1 | -498 | 0 |
| OUM-regime-split | 2 | -496 | 2.75 |
| OUM-echo-freq | 3 | -449 | 48.94 |
| OUM-ancestral | 4 | -424 | 74.7 |
| OUM-echo | 5 | -422 | 76.75 |
| BMM-echo | 6 | -402 | 96.02 |
| BMMtr-echo | 7 | -396 | 102.86 |
| BMMsm-echo | 8 | -394 | 104.47 |
| BMMsm-echo-freq | 9 | -391 | 107.78 |
| OU-ancestral | 10 | -390 | 108.08 |
| BMM-echo-freq | 11 | -380 | 117.99 |
| BMMtr-echo-freq | 12 | -379 | 119.26 |
| BMMsm-regime | 13 | -357 | 141.54 |
| BMMsm-regime-split | 14 | -356 | 142.39 |
| BMMtr-ancestral | 15 | -348 | 150.6 |
| BMM-ancestral | 16 | -346 | 151.89 |
| BMM-regime | 17 | -346 | 152.6 |
| BMM-regime-split | 18 | -345 | 153.8 |
| BMMtr-regime | 19 | -344 | 154.4 |
| BMMsm-ancestral | 20 | -344 | 154.81 |
| BMMtr-regime-split | 21 | -342 | 156.4 |
| BM-ancestral | 22 | -341 | 156.92 |
| BMtr-ancestral | 23 | -341 | 156.6 |
| BMsm-ancestral | 24 | -338 | 160.83 |

c)

| Model | Rank | AIC | diff |
| --- | --- | --- | --- |
| OUM-regime | 1 | -383 | 0 |
| OUM-regime-split | 2 | -380 | 3.41 |
| OUM-echo-freq | 3 | -354 | 29.07 |
| OUM-ancestral | 4 | -329 | 54.22 |
| OUM-echo | 5 | -323 | 60.2 |
| BMM-echo | 6 | -304 | 79 |
| BMMtr-echo | 7 | -302 | 80.72 |
| BMMsm-echo | 8 | -300 | 83.3 |
| OU-ancestral | 9 | -292 | 91.36 |
| BMM-echo-freq | 10 | -269 | 113.84 |
| BMMtr-echo-freq | 11 | -267 | 115.76 |
| BMMsm-echo-freq | 12 | -266 | 116.84 |
| BMMsm-echo-freq | 13 | -228 | 155.43 |
| BMMsm-regime | 14 | -227 | 156.07 |
| BMM-ancestral | 15 | -221 | 162.32 |
| BMMtr-ancestral | 16 | -219 | 164.03 |
| BMM-regime | 17 | -219 | 164.08 |
| BMMtr-regime | 18 | -217 | 165.92 |
| BMMsm-regime | 19 | -217 | 166 |
| BMM-regime-split | 20 | -217 | 166.57 |
| BMMtr-regime-split | 21 | -215 | 168.53 |
| BM-ancestral | 22 | -208 | 175.58 |
| BMtr-ancestral | 23 | -206 | 177.46 |
| BMsm-ancestral | 24 | -204 | 179.45 |


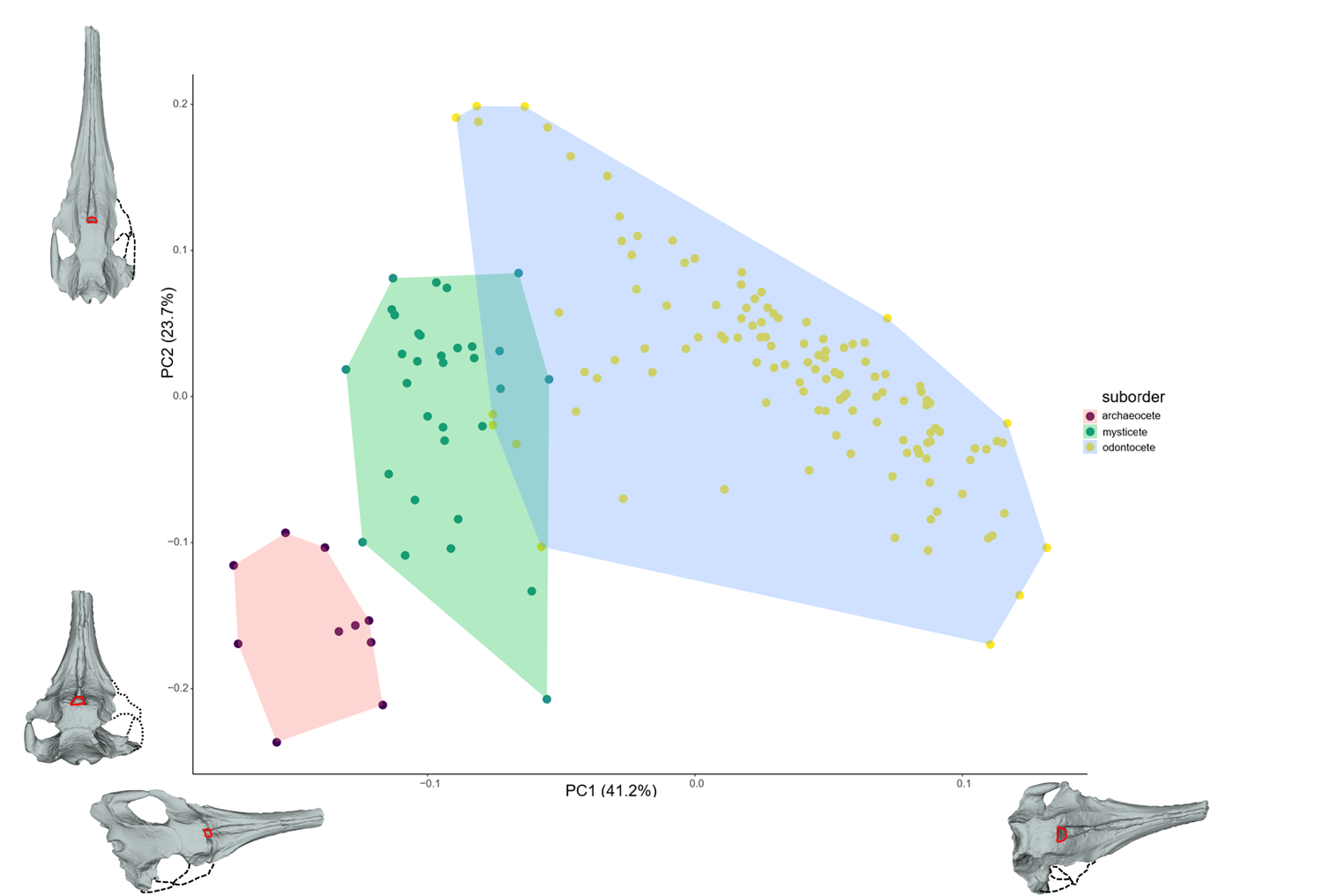


Fig S4. Additional morphospace occupation of cetacean crania used in this study. Principal components (PC) 1 and 2 using 123 landmarks over the surface of the skull. PC1 (41.2%) shows variation in the positioning of the nares – the shift posteriorly on the skull from archaeocetes to extant cetaceans. PC2 (23.7%) shows variation in the length of the rostrum. Axes extremes indicated on a warped skull of a patriocetid. On the skulls; dotted lines show missing data, and the red outline indicates the position of the nares. n = 162.


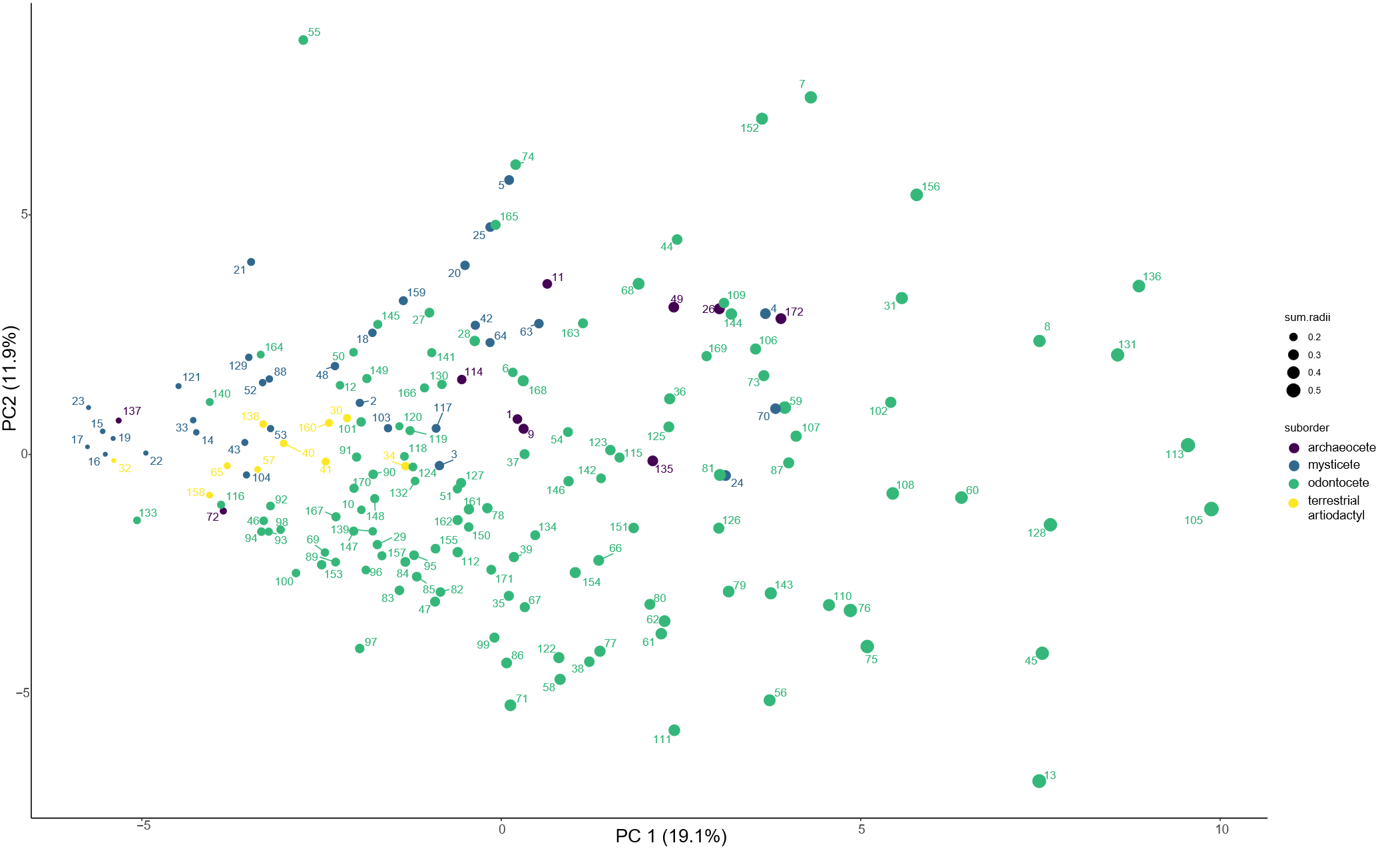


[previous page]

Fig S5. Principal Components plot with PC1 and PC2 for each specimen in the study. The PC scores represent the sum radii (∑*p*_spec_) in the skull for each specimen. The larger the circle, the higher the ∑*p*_spec_. Terrestrial artiodactyls are shown in yellow, archaeocetes in deep purple, mysticetes in blue, and odontocetes in green. For number corresponding to specimen see Table S9 - List of specimens used in the study. n = 172, including 10 terrestrial artiodactyls.

# Model diagnostics

We ran diagnostics on our two proposals to test their robustness. We assessed the trace of the variable (chain) for each of the models to ensure that burn-in had completed (we used the default value of 0.25 for the burn-in). The trace of the shifts (supplemental figs) shows that the burn-in occurred long before 25% of the iterations had run, we therefore know that the default value is a safe assumption to use. We then assessed the trace to check that the MCMC sampler mixed well (i.e., it remains stationary after an initial burn-in) (Supplemental FIGS of Trace of vars for both models). The same chain was obtained when the model was run with 5^6 iterations, and a sampling frequency of 5,000, as well as with different starting values. If the runs had not converged, longer MCMC chains would have been run. We also checked the effective sample size (ESS) using the coda package in R [34]. This looks at the effective sample size for controlling the mean when the sample size has been adjusted for autocorrelation. Here the ES values for each model were all much higher than 100-200 (the generally accepted lower value of the standard error (SE), root, and likelihood value for each chain). See the supplemental information for the ES values of these two chains. We can also see the point (where the chains roughly converge) on the Gelman plot **(Fig. S10** - Gelman diagnostics for the two chains). We then used Gelman and Rubin’s convergence diagnostics [35, 36] to get the scale reduction factor for each parameter. For these chains the point estimate for the potential scale reduction was 1, and the associated upper confidence limit was 1.01. Generally, values below 1.1 are accepted as a ‘good’ fit.


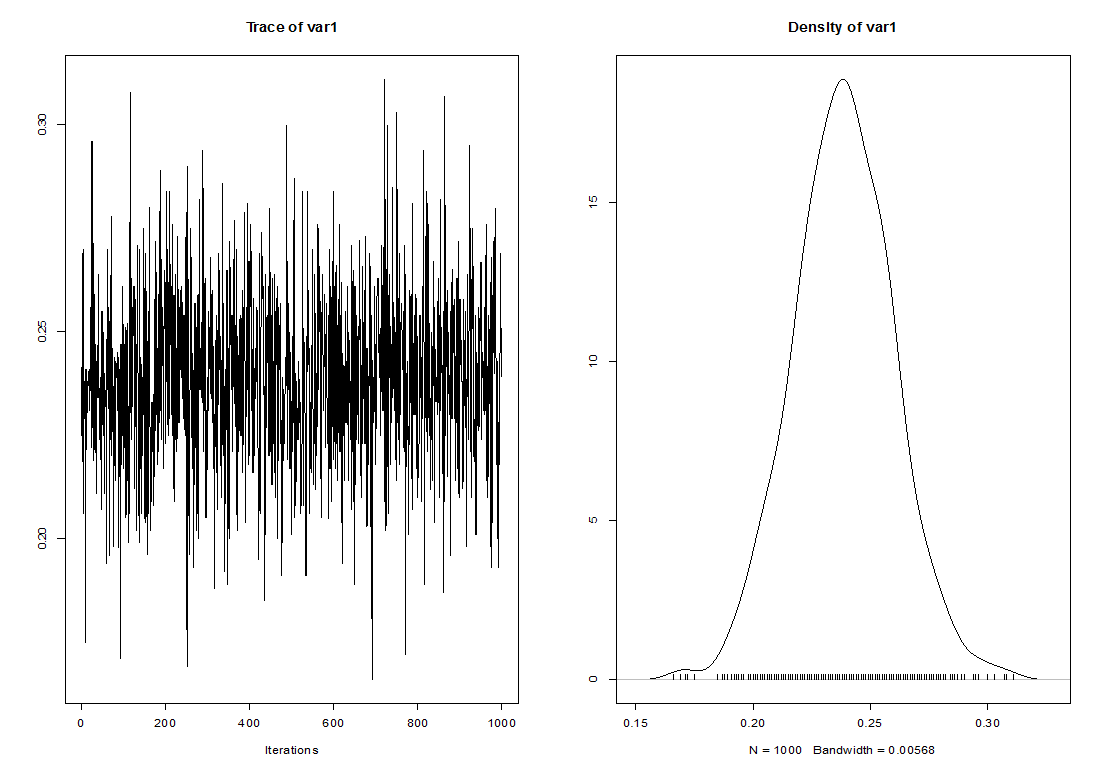


Fig S6. Trace of the chain for model 1. The plot on the left, the trace plot shows the values the parameter took during the runtime of the chain (1000 iterations). The plot on the right, the density var plot (or marginal density plot) is the (smoothened) histogram of the values in the trace-plot, i.e. the distribution of the values of the parameter in the chain. Note the similarities between the models (**Fig S7** - Trace of the chain for model 2).


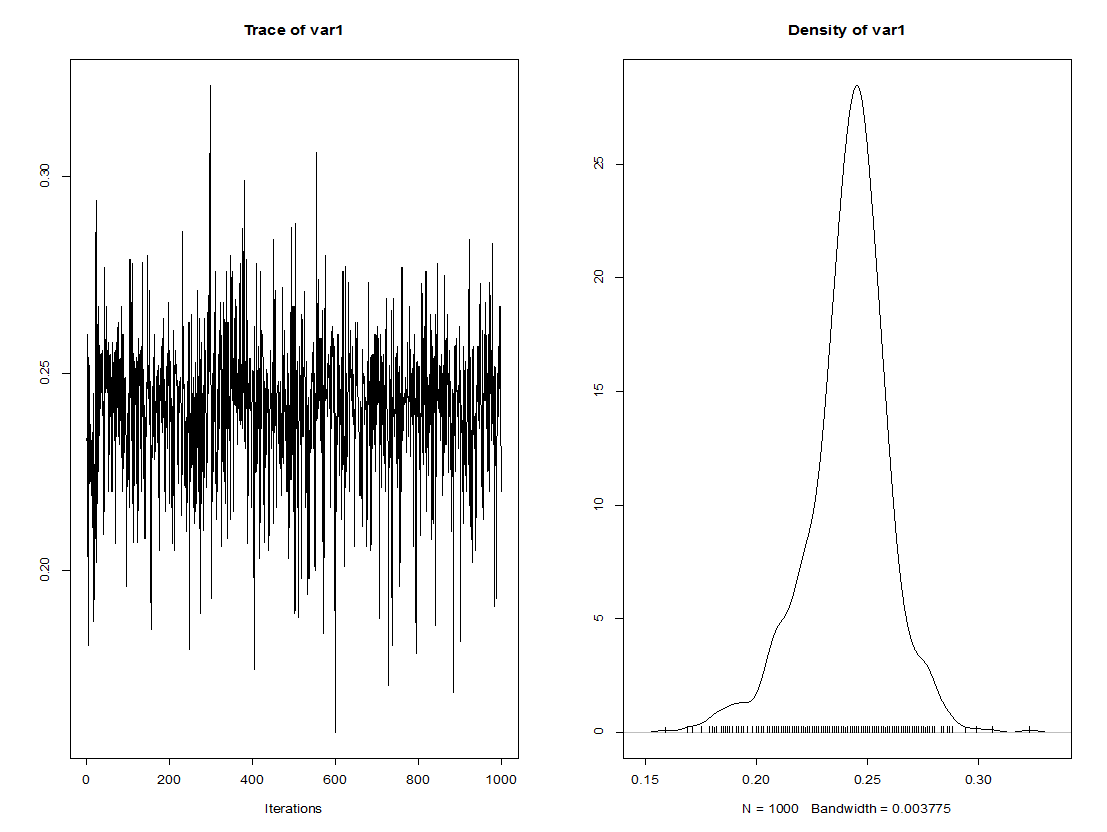


Fig S7. Trace of the chain for model 2. The plot on the left, the trace plot shows the values the parameter took during the runtime of the chain (1000 iterations). The plot on the right, the density var plot (or marginal density plot) is the (smoothened) histogram of the values in the trace-plot, i.e. the distribution of the values of the parameter in the chain. Note the similarities between the models (**Fig S6** - Trace of the chain for model 1).

Fig S8. Further model diagnostics for chain 1. Visualisation of results in **Table S6** - Effective size (ES) for estimating the mean for each of the chains 1 and 2. The trace plots (left hand column) shows the values the parameter took during the runtime of the chain (1000 iterations). Note the early burn-in and then stabilisation of the chain before 250 iterations (25%). The density plots (right hand column) (or marginal density plots) are the (smoothened) histogram of the values in the trace-plot, i.e., the distribution of the values of the parameter in the chain.

Fig S9. Further model diagnostics for chain 2. Visualisation of results in **Table S6** - Effective size (ES) for estimating the mean for each of the chains 1 and 2. The trace plots (left hand column) shows the values the parameter took during the runtime of the chain (1000 iterations). Note the early burn-in and then stabilisation of the chain before 250 iterations (25%). The density plots (right hand column) (or marginal density plots) are the (smoothened) histogram of the values in the trace-plot, i.e., the distribution of the values of the parameter in the chain.

Table S6. Effective size (ES) for estimating the mean for each of the chains 1 and 2. Chains used in our main model (model 1) (Fig 4; Fig 5) and in an alternate model (named model 2, chain 2 here). Using the effectiveSize function in the R package coda [34] to look at the sample size adjusted for autocorrelation. Here the ES values for each model were all higher than 100-200 (the generally accepted lower value of the standard error (SE), root, and likelihood value for each chain). There are no jumps, and therefore jumps.var is zero in chain 1(Model 1). Outputs are visualised in **Fig S6 -**  Trace of the chain for model 1 and **Fig S7** - Trace of the chain for model 2.

Chain 1

| min | max | median | shifts | jumps | jumpvar | SE | root | InL | ppos |
| --- | --- | --- | --- | --- | --- | --- | --- | --- | --- |
| 0.0000 | 1000.0000 | 1000.0000 | 760.4919 | 0.0000 | 0.0000 | 848.8171 | 1000.000 | 903.8943 | 1000.0000 |

Chain 2

| min | max | median | shifts | jumps | jumpvar | SE | root | InL | ppos |
| --- | --- | --- | --- | --- | --- | --- | --- | --- | --- |
| 0.0000 | 0.0000 | 0.0000 | 0.0000 | 298.48390 | 645.49524 | 863.92187 | 1000.00000 | 80.65584 | 125.28040 |

a)


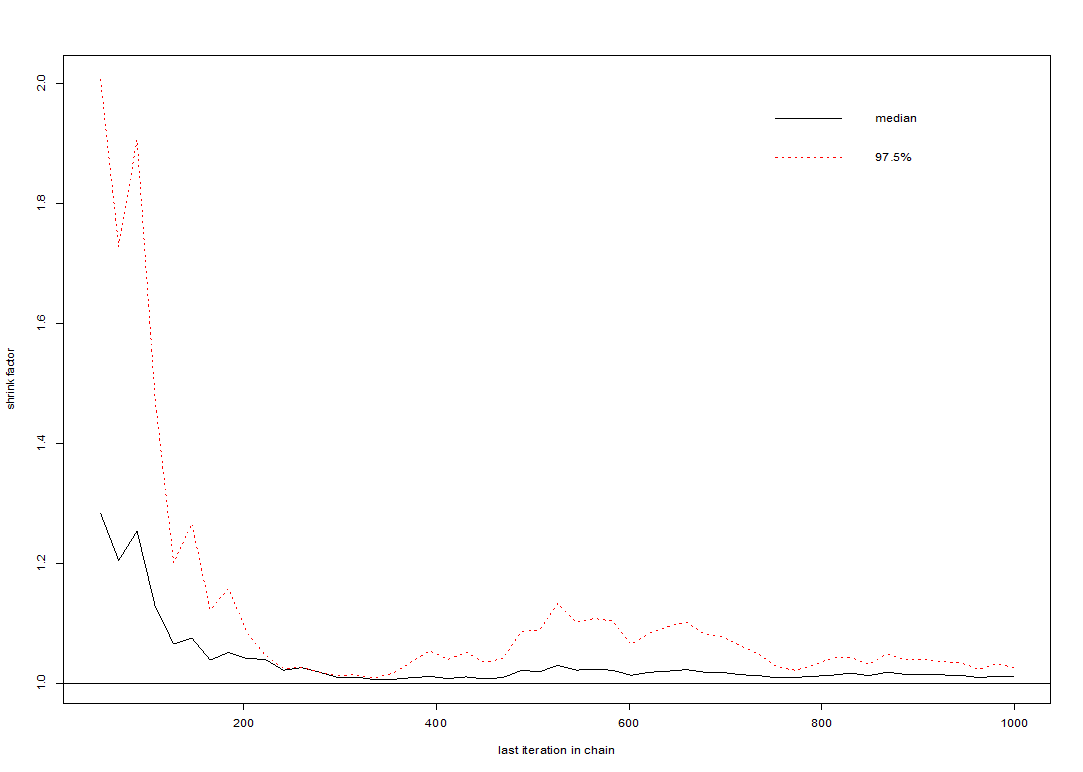


b)


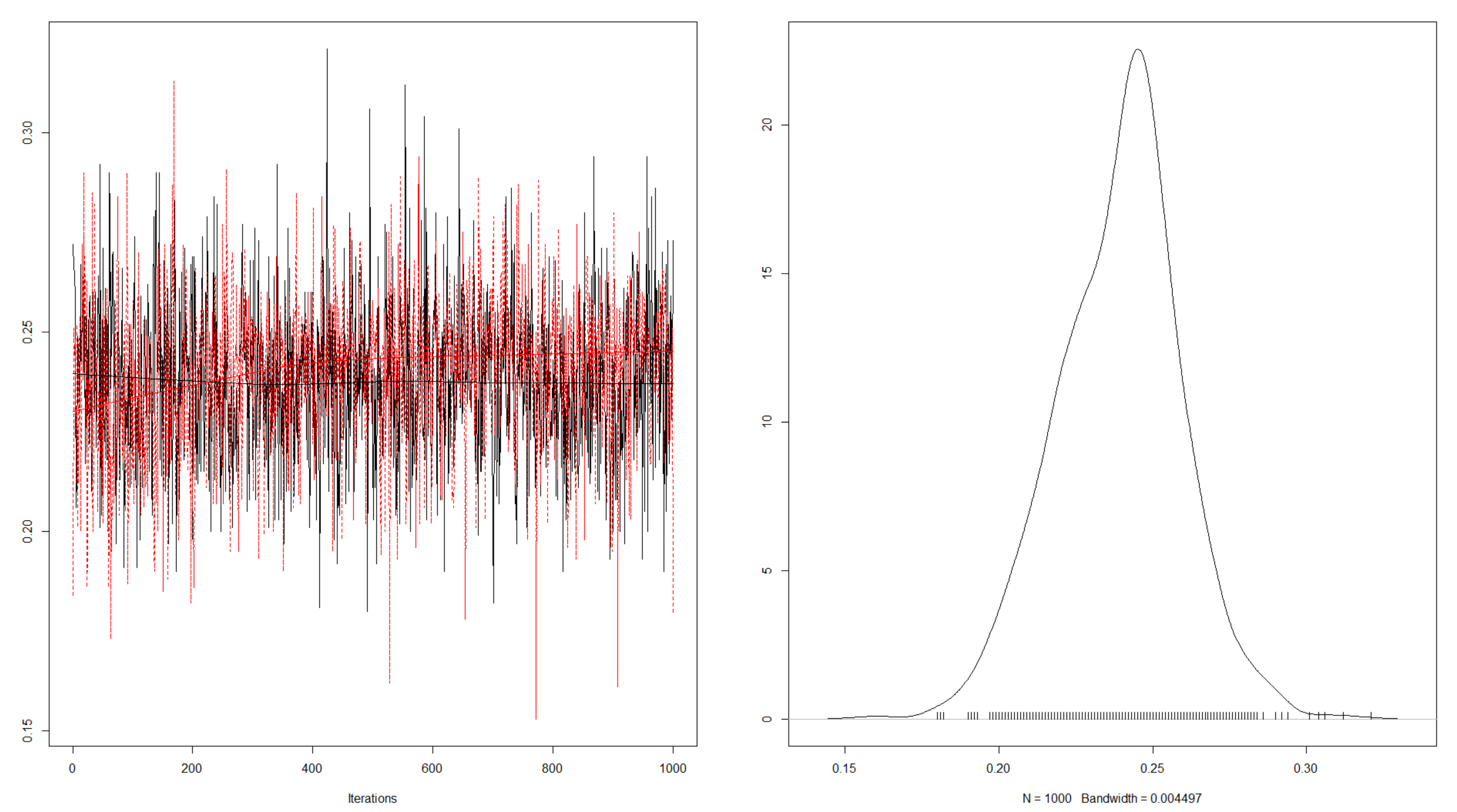


[this and previous page]

Fig S10. Gelman diagnostics for the two chains. a) The evolution of the shrink factor [35, 36] as the number of iterations increases. The gelman.diag gives you the scale reduction factors for each parameter. Approximate convergence is diagnosed when the upper limit is close to 1. A factor of 1 means that between variance and within chain variance are equal. Larger values mean that there is still a notable difference between chains. A values of 1.1 or below is generally accepted. The confidence limits assume that the variable is normal. This plot was run as a diagnostic based on our chains which had 10^6 iterations. The plot shows the development of the scale-reduction over time (chain steps). This is useful because it allows you to see whether a low chain reduction is also stable. b) The convergence of the chains.


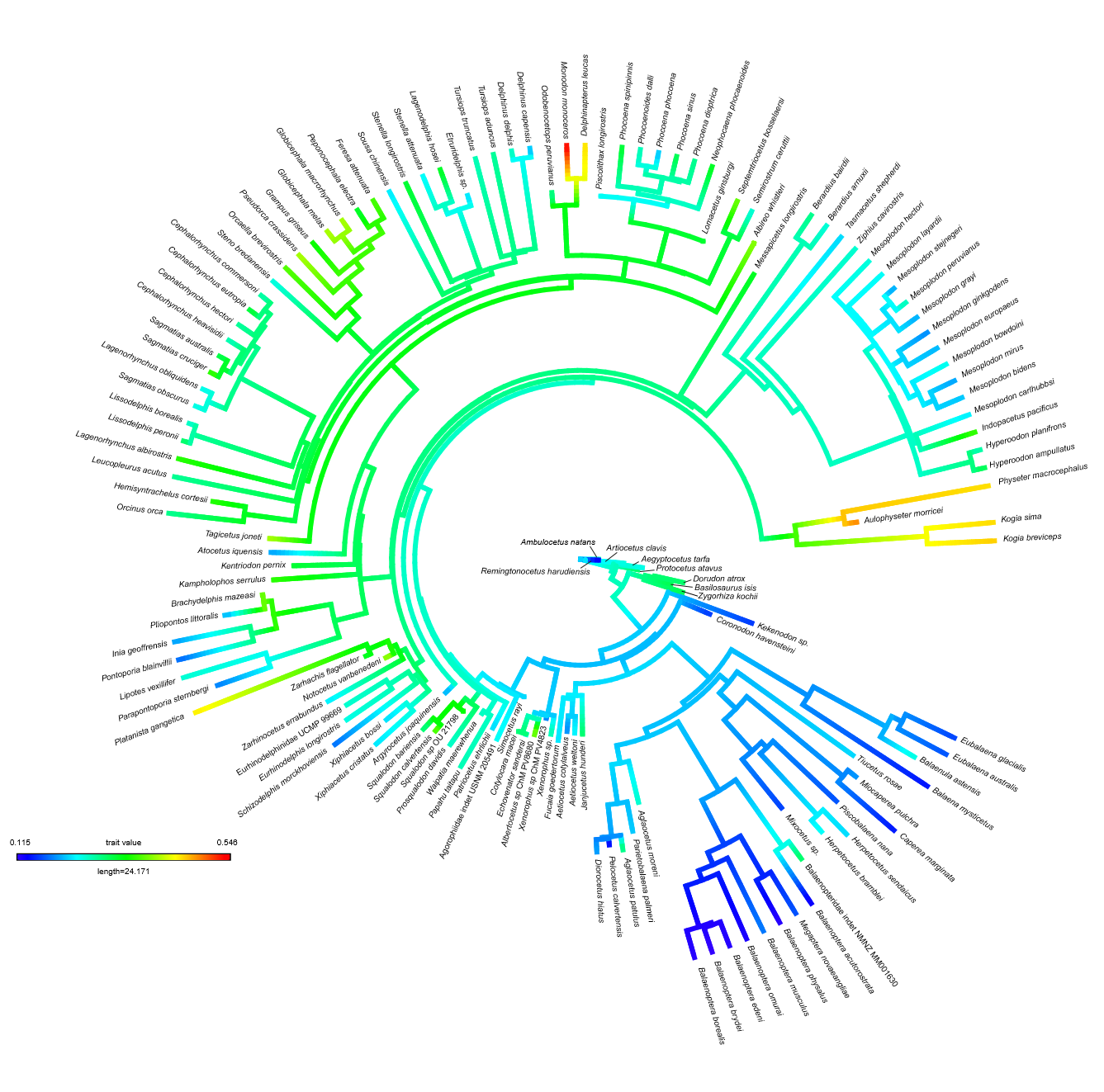


[previous page]

Fig S11. Asymmetry in the cetacean skull shown using a phylogeny that includes only taxa that appear in a character matrix. Adapted from Lloyd and Slater [29]. Phylogeny for sampled cetacean species with branches showing the level of asymmetry (∑*p*_spec_). The trait value is the sum of Euclidean distances between the computer mirrored landmark and the manually placed landmark. The larger the value for Σ*p*_spec_, the more the landmarks have been displaced, indicating asymmetry between the two sides of the cranium. The full complement of landmarks was used.


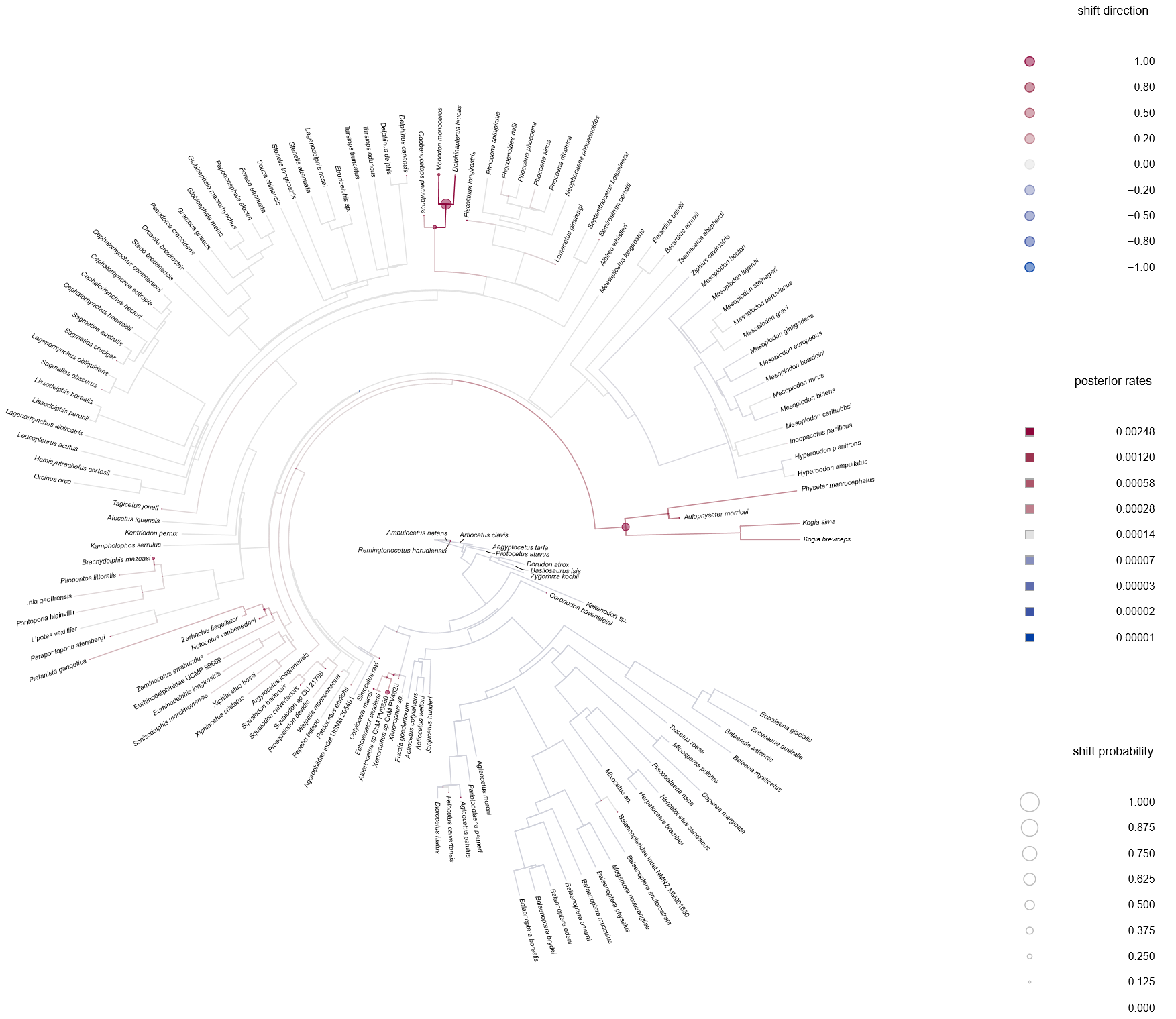


[Previous page]

Fig S12. Reconstructed probability of shifts in cetacean cranial asymmetry (∑*p*_spec_) using a phylogeny that includes only taxa that appear in a character matrix. Adapted from Lloyd and Slater [29]. Probability of shifts are shown along each branch of the phylogeny under the assumption of relaxed Brownian Motion with a Half-Cauchy distribution for the prior density of the rate scalar. Circles indicate a shift in the trait on either the branch or in the whole clade. The colour of the circle indicates the shift direction with red indicating forward shifts and blue indicating backwards shifts. The size of the circle indicates the probability of the shift occurring in that position in the clade with the largest circle (here, ~0.500) indicating the highest probability of a shift occurring. The colour of the branch itself indicates posterior rates for that branch with red showing higher, increasing rates and blue showing lower, decreasing rates. The background rate is shown as grey. Phylogeny based on Lloyd and Slater [29]. Full complement of landmarks used.


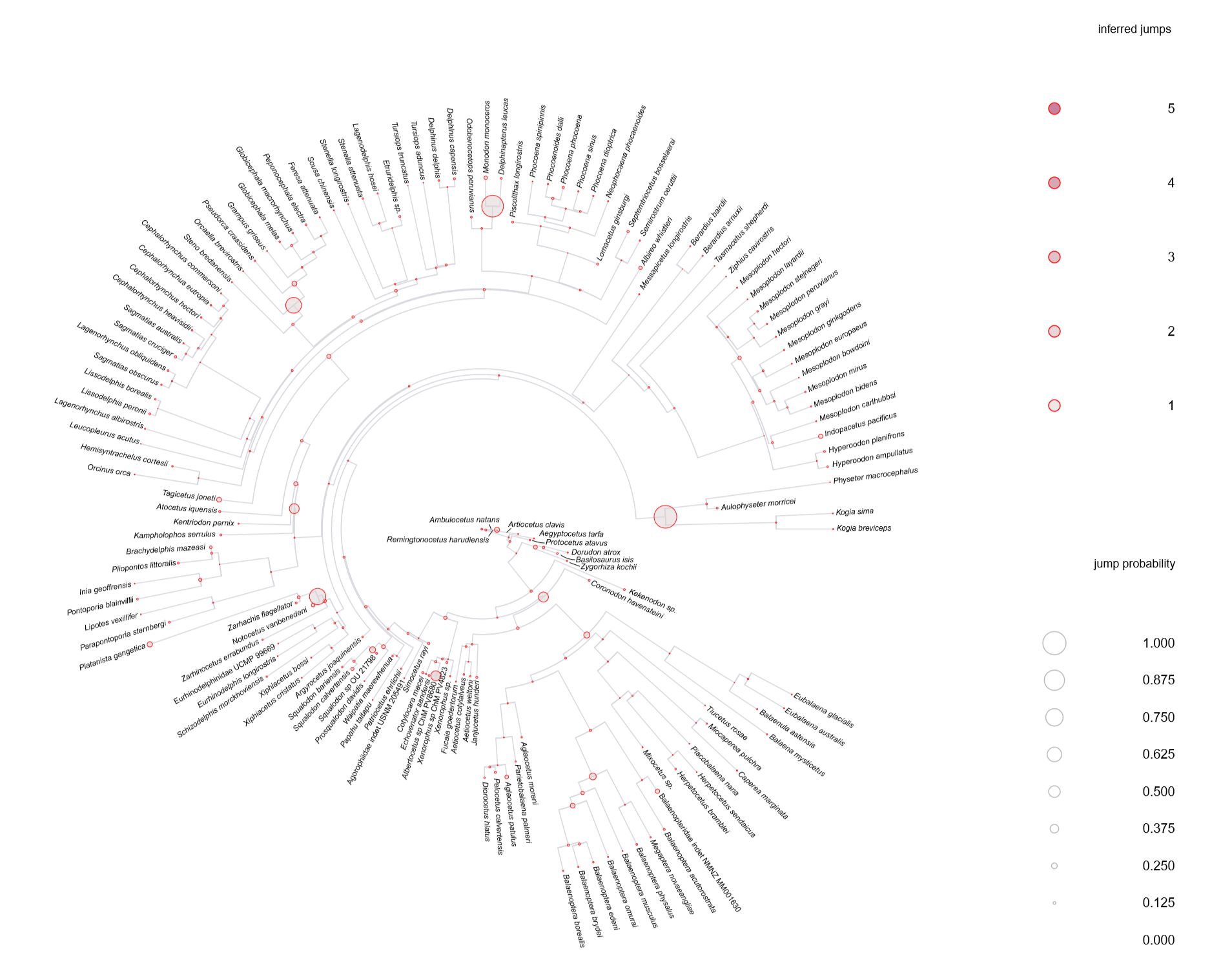


[previous page]

Fig S13. Reconstructed jumps in the rate of cetacean cranial asymmetry (∑*p*_spec_) using a phylogeny that includes only taxa that appear in a character matrix. Adapted from Lloyd and Slater [29]. The model also predicts the number of jumps which may have occurred. The size of the circle indicates the probability of the jump occurring there. The colour of the circle indicates the number of inferred jumps, where dark red = 5 and pale red = 1. Full complement of landmarks used.

Table S7. ANOVA results for each potential scenario for asymmetry in the cetacean cranium. ANOVAs are run with Pagel's Lambda Correlation Structure (corPagel). Degrees of freedom (DF), p-value (*p*), F-value (F), and *p*-value adjusted using the Benjamini-Hochberg method for false discovery rate [55, 56] are shown.

| **Model** | **Summary** | **ANOVA (corPagel)** | | | |
| --- | --- | --- | --- | --- | --- |
|  |  | **DF (degrees of freedom)** | **F** | ***p*** | **Adjusted *p***  **(Benjamini-Hochberg)** |
| Geological age | Geological age of the species | 155 | 1.10 | 0.36 | 0.36 |
| Suborder | Split via suborder: archaecoete, mysticete, odontocete | 159 | 1.57 | 0.21 | 0.28 |
| Regime | Assumes selective evolutionary regimes for highly asymmetric taxa | 158 | 26.97 | < 0.001 | < 0.001 |
| Regime split | Each highly asymmetric group is evolving under its own separate selective regime | 156 | 15.78 | < 0.001 | < 0.01 |
| Echolocation | Presence or absence of the ability to echolocate | 158 | 1.44 | 0.23 | 0.28 |
| Echolocation frequency | The frequency at which a cetacean echolocates/produces sound | 155 | 5.83 | < 0.001 | < 0.001 |

**Echolocation frequency categories - supporting paragraph**

This model was based on categorising by echolocation in the extant odontocetes, and sound production in the extant mysticetes. Data on frequency specifics is not available for fossil data. The narrowband high-frequency (NBHF) cetaceans were designated as so according to Kastelein et al. [40] and Morisaka et al. [72], among others. The non-NBHF delphinids were assigned to broadband low frequency (BBLF) according to to Jensen et al. [43], and Ladegaard et al. [71] among others. The sperm whale sits in its own category because the hypertrophied nasal structures and deep-diving behaviour produce a low-frequency multi-pulsed sound [45] which does not fit into any of our categories. The beaked whales (Ziphiidae) did not fit in with any of the above categories so were put into their own category based on their acoustic repertoire of frequency-modulated buzz clicks [46-50]. Mysticetes do not echolocate and were put into a separate category; low frequency mysticetes (LFM). The monodontidae were separated into their own category based on their unique sound repertoire (narrowband structured (NBS)) comprising pulses [52-54] ideal for projecting and receiving signals in shallow and cluttered, icy water [53] and further on the unique morphology of the inner ear which occupies a tightly constrained area showing clear separation from odontocetes which use NBHF echolocation [54].

Table S8. Frequency categories used to group all extant cetaceans for the ‘frequency echolocation’ model. A description of the band width type, the taxa within that bandwidth, and supporting references are given.

| **Band width (acronym)** | **Taxa** | **References** |
| --- | --- | --- |
| Unknown | All fossil taxa | NA |
| Broad band low frequency (BBLF) | All non-narrow-band high frequency (NBHF) delphinids | [43, 44, 64, 70, 71] |
| Frequency modulated buzz clicks (FMbuzz) | Ziphiids | [46-50] |
| Low frequency mysticetes (LFM) | All mysticetes | [24, 51] |
| Low frequency multi pulsed (LFMP) | *Physeter macrocephalus* | [45] |
| Narrow-band high frequency (NBHF) | Phocoenidae, *Inia*, *Pontoporia*, *Cephalorhynchus*, *Kogia*, *Lissodelphis* | [40-42, 72] |
| Narrow-band structured (NBS) | Monodontidae | [52-54] |


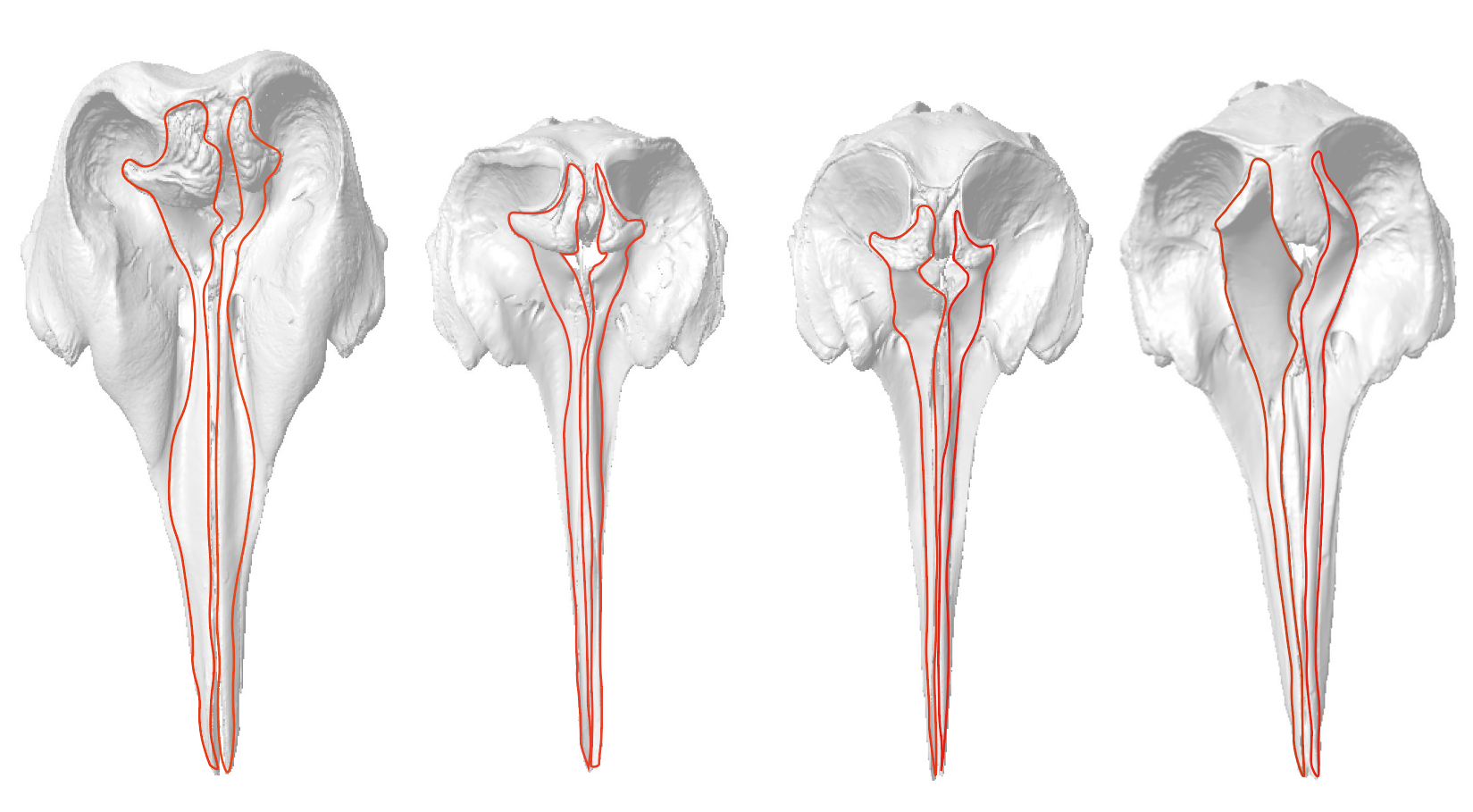


Fig S14. Ziphiid skulls showing the marked asymmetry in the premaxillary crests (outlined in red). From left to right: *Hyperoodon planifrons* NHMUK 1952.9.30.1, *Mesoplodon carlhubbsi* USNM 504128, *Mesoplodon mirus* USNM 504612, *Ziphius cavirostris* NHMUK 2006.15. Not to scale.

[next page]

Table S9. List of specimens used in the study. *Denotes terrestrial artiodactyls. Data on the specimen and ID, species, suborder, family, and geologic age are given. ^+^Name in brackets denotes genus name given in phylogeny.

|  | **Specimen** | **Species** | **Group** | **Family** | **Geologic age** |
| --- | --- | --- | --- | --- | --- |
| **1** | *Aegyptocetus tarfa* MSNTUP I-15459 | *Aegyptocetus tarfa* | Archaeocete | Protocetidae | Eocene |
| **2** | *Aetiocetus cotylalveus* USNM 25210 | *Aetiocetus cotylalveus* | Mysticete | Aetiocetidae | Oligocene |
| **3** | *Aetiocetus weltoni* UCMP 122900 | *Aetiocetus weltoni* | Mysticete | Aetiocetidae | Oligocene |
| **4** | *Aglaocetus patulus* USNM 23690 | *Aglaocetus patulus* | Mysticete | Aglaocetidae | Miocene |
| **5** | *Aglaocetus moreni* FMNH P13407 | *Aglaocetus moreni* | Mysticete | Aglaocetidae | Miocene |
| **6** | Agorophiid *sp* USNM 205491 | Agorophiid *sp* | Odontocete | Agorophiidae | Oligocene |
| **7** | *Albertocetus* ChM PV8680 | *Albertocetus sp* | Odontocete | Xenorophidae | Oligocene |
| **8** | *Albireo whistleri* UCR 14589 | *Albireo whistleri* | Odontocete | Albireonidae | Miocene |
| **9** | *Ambulocetus natans* MSNUP I-16826 | *Ambulocetus natans* | Archaeocete | Ambulocetidae | Eocene |
| **10** | *Argyrocetus joaquinensis* USNM 11996 | *Argyrocetus joaquinensis* | Odontocete | Unclear | Miocene |
| **11** | *Artiocetus clavis* GSP-UM 3458 | *Artiocetus clavis* | Archaeocete | Protocetidae | Eocene |
| **12** | *Atocetus iquensis* MNHN.F.PPI. 113 | *Atocetus iquensis* | Odontocete | Delphinida | Miocene |
| **13** | *Aulophyseter* *morricei* UCMP 81661 | *Aulophyseter morricei* | Odontocete | Physeteridae | Miocene |
| **14** | *Balaena mysticetus* NHMUK 1986.1.16 | *Balaena mysticetus* | Mysticete | Balaenidae | Extant |
| **15** | *Balaenoptera acutorostrata* NHMUK 1965.11.2.1 | *Balaenoptera acutorostrata* | Mysticete | Balaenopteridae | Extant |
| **16** | *Balaenoptera borealis* NHMUK 1934.5.25.1 | *Balaenoptera borealis* | Mysticete | Balaenopteridae | Extant |
| **17** | *Balaenoptera brydei* USNM 572922 | *Balaenoptera brydei* | Mysticete | Balaenopteridae | Extant |
| **18** | *Balaenoptera sp* SDNHM 83695 | *Balaenoptera sp* | Mysticete | Balaenopteridae | Pliocene |
|  |  |  |  |  |  |
| **19** | *Balaenoptera edeni* NHMUK 1920.12.31.1 | *Balaenoptera edeni* | Mysticete | Balaenopteridae | Extant |
| **20** | *Balaenoptera floridana* USNM 529244 | *Balaenoptera floridana* | Mysticete | Balaenopteridae | Pliocene |
| **21** | *Balaenoptera musculus* NHMUK 1892.3.1.1 | *Balaenoptera musculus* | Mysticete | Balaenopteridae | Extant |
| **22** | *Balaenoptera omurai* NN – data: <https://www.cityu.edu.hk/lib/about/event/tk_brown/> | *Balaenoptera omurai* | Mysticete | Balaenopteridae | Extant |
| **23** | *Balaenoptera physalus* NHMUK 1862.2.7.181 | *Balaenoptera physalus* | Mysticete | Balaenopteridae | Extant |
| **24** | Balaenopteridae NMNZ MM001630 | *Balaenopteridae* | Mysticete | Balaenopteridae | Miocene |
| **25** | *Balaenula astensis* MSNUP I-12555 | *Balaenula astensis* | Mysticete | Balaenidae | Pliocene |
| **26** | *Basilosaurus isis* SMNS 11787 | *Basilosaurus isis* | Archaeocete | Basilosauridae | Eocene |
| **27** | *Berardius arnuxii* NHMUK 1935.10.23.1 | *Berardius arnuxii* | Odontocete | Ziphiidae | Extant |
| **28** | *Berardius bairdii* NHMUK 1954.9.21.1 | *Berardius bairdii* | Odontocete | Ziphiidae | Extant |
| **29** | *Berardius minimus* USNM 276366 | *Berardius bairdii* | Odontocete | Ziphiidae | Extant |
| **30** | **Bos* *sp* NHMUK 1981.984 | *Bos sp* | Terrestrial artiodactyl | Bovidae | Extant |
| **31** | *Brachydelphis mazeasi* MNHN.F.PPI. 266 | *Brachydelphis mazeasi* | Odontocete | Pontoporiidae | Miocene |
| **32** | **Camelus dromedarius* NHMUK NN | *Camelus dromedarius* | Terrestrial artiodactyl | Camelidae | Extant |
| **33** | *Caperea marginata* NHMUK 1876.2.16.1 | *Caperea marginata* | Mysticete | Cetotheriidae | Extant |
| **34** | **Capricornis sumatrensis* NHMUK 24.5.29.1 | *Capricornis sumatrensis* | Terrestrial artiodactyl | Bovidae | Extant |
| **35** | *Cephalorhynchus commersonii* USNM 252568 | *Cephalorhynchus commersonii* | Odontocete | Delphinidae | Extant |
| **36** | *Cephalorhynchus eutropia* NHMUK 1881.8.17.1 | *Cephalorhynchus eutropia* | Odontocete | Delphinidae | Extant |
| **37** | *Cephalorhynchus heavisidii* NHMUK 1948.7.27.1 | *Cephalorhynchus heavisidii* | Odontocete | Delphinidae | Extant |
| **38** | *Cephalorhynchus hectori maui* NMNZ MM002607 | *Cephalorhynchus hectori maui* | Odontocete | Delphinidae | Extant |
| **39** | *Cephalorhynchus hectori* NMNZ MM002288 | *Cephalorhynchus hectori* | Odontocete | Delphinidae | Extant |
| **40** | **Cervus elaphus* NHMUK 2005.16 | *Cervus elaphus* | Terrestrial artiodactyl | Cervidae | Extant |
| **41** | **Choeropsis liberiensis* NHMUK 1967.3.20.1 | *Choeropsis liberiensis* | Terrestrial artiodactyl | Hippopotamidae | Extant |
| **42** | *Chonecetus goedertorum* LACM 131146  (^+^*Fucaia*) | *Chonecetus goedertorum* | Mysticete | Aetiocetidae | Oligocene |
| **43** | *Coronodon havensteini* CCNHM 108 | *Coronodon havensteini* | Mysticete | Aetiocetidae | Oligocene |
| **44** | *Cotylocara macei* CCNHM 101 | *Cotylocara macei* | Odontocete | Xenorophidae | Oligocene |
| **45** | *Delphinapterus leucas* USNM 305071 | *Delphinapterus leucas* | Odontocete | Monodontidae | Extant |
| **46** | *Delphinus capensis* NHMUK 1981.7.11 | *Delphinus capensis* | Odontocete | Delphinidae | Extant |
| **47** | *Delphinus delphis* AMNH 75332 | *Delphinus delphis* | Odontocete | Delphinidae | Extant |
| **48** | *Diorocetus hiatus* USNM 16783 | *Diorocetus hiatus* | Mysticete | Pelocetidae | Miocene |
| **49** | *Dorudon* *atrox* PV M 100149 cast | *Dorudon atrox* | Archaeocete | Basilosauridae | Eocene |
| **50** | *Echovenator sandersi* GSM 1098 | *Echovenator sandersi* | Odontocete | Xenorophidae | Oligocene |
| **51** | *Etruridelphis* *sp* MGPT PU 13884 | *Etruridelphis sp* | Odontocete | Delphinidae | Pliocene |
| **52** | *Eubalaena australis* NHMUK 1873.3.3 | *Eubalaena australis* | Mysticete | Balaenidae | Extant |
| **53** | *Eubalaena glacialis* MSNUP NN | *Eubalaena glacialis* | Mysticete | Balaenidae | Extant |
| **54** | Eurhinodelphinidae UCMP 99669 | *Eurhinodelphinidae* | Odontocete | Eurhinodelphinidae | Miocene |
| **55** | *Eurhinodelphis longirostris* USNM 244404 | *Eurhinodelphis longirostris* | Odontocete | Eurhinodelphinidae | Miocene |
| **56** | *Feresa attenuata* USNM 504916 | *Feresa attenuata* | Odontocete | Delphinidae | Extant |
| **57** | **Giraffa camelopardalis* NHMUK NN | *Giraffa camelopardalis* | Terrestrial artiodactyl | Giraffidae | Extant |
| **58** | *Globicephal*a *sp* USNM 21867 | *Globicephala sp* | Odontocete | Delphinidae | Pleistocene |
| **59** | *Globicephala macrorhynchus* NHMUK 1912.10.27 | *Globicephala macrorhynchus* | Odontocete | Delphinidae | Extant |
| **60** | *Globicephala melas* NMNZ MM001946 | *Globicephala melas* | Odontocete | Delphinidae | Extant |
| **61** | *Grampus griseus* USNM 571602 | *Grampus griseus* | Odontocete | Delphinidae | Extant |
| **62** | *Hemisyntrachelus cortesii* MBGPT NN cast | *Hemisyntrachelus cortesii* | Odontocete | Delphinidae | Pliocene |
| **63** | *Herpetocetus bramblei* UCMP 219111 | *Herpetocetus bramblei* | Mysticete | Cetotheriidae | Pliocene |
| **64** | *Herpetocetus sendaicus* NMNS-PV 19540 cast | *Herpetocetus sendaicus* | Mysticete | Cetotheriidae | Pliocene |
| **65** | **Hydropotes inemis* NHMUK 1551c | *Hydropotes inemis* | Terrestrial artiodactyl | Cervidae | Extant |
| **66** | *Hyperoodon ampullatus* NHMUK 1992.42 | *Hyperoodon ampullatus* | Odontocete | Ziphiidae | Extant |
| **67** | *Hyperoodon planifrons* NHMUK 1952.9.30.1 | *Hyperoodon planifrons* | Odontocete | Ziphiidae | Extant |
| **68** | *Indopacetus pacificus* USNM 593534 | *Indopacetus pacificus* | Odontocete | Ziphiidae | Extant |
| **69** | *Inia geoffrensis* AMNH 93415 | *Inia geoffrensis* | Odontocete | Iniidae | Extant |
| **70** | *Janjucetus hunderi* NMV P216929 | *Janjucetus hunderi* | Mysticete | Mammalodontidae | Oligocene |
| **71** | *Kampholophus serrulus* UMCP 36045 | *Kampholophus serrulus* | Odontocete | Kentriodontidae | Miocene |
| **72** | *Kekenodon* *sp* OU 22294 | *Kekenodon sp* | Archaeocete | Kekenodontidae | Oligocene |
| **73** | *Kentriodon pernix* USNM 10670 | *Kentriodon pernix* | Odontocete | Kentriodontidae | Miocene |
| **74** | *Kentriodon sp NN* | *Kentriodon sp* | Odontocete | Kentriodontidae | Miocene |
| **75** | *Kogia breviceps* USNM 22015 | *Kogia breviceps* | Odontocete | Kogiidae | Extant |
| **76** | *Kogia simus* NHMUK.1952.8.28.1 | *Kogia simus* | Odontocete | Kogiidae | Extant |
| **77** | *Lagenodelphis hosei* USNM 571619 | *Lagenodelphis hosei* | Odontocete | Delphinidae | Extant |
| **78** | *Lagenorhynchus acutus* USNM 504196  (^+^*Leucopleurus*) | *Lagenorhynchus acutus*  (^+^*Leucopleurus*) | Odontocete | Delphinidae | Extant |
| **79** | *Lagenorhynchus albirostris* AMNH 37162 | *Lagenorhynchus albirostris* | Odontocete | Delphinidae | Extant |
| **80** | *Lagenorhynchus australis* 1944.11.30.1  (^+^*Sagmatias*) | *Lagenorhynchus australis*  (^+^*Sagmatias*) | Odontocete | Delphinidae | Extant |
| **81** | *Lagenorhynchus cruciger* NHMUK 1960.8.24.1  (^+^*Sagmatias*) | *Lagenorhynchus cruciger*  (^+^*Sagmatias*) | Odontocete | Delphinidae | Extant |
| **82** | *Lagenorhynchus obliquidens* NHMUK 1992.83 | *Lagenorhynchus obliquidens* | Odontocete | Delphinidae | Extant |
| **83** | *Lagenorhynchus obscurus* NHMUK 1846.3.11.8  (^+^*Sagmatias*) | *Lagenorhynchus obscurus*  (^+^*Sagmatias*) | Odontocete | Delphinidae | Extant |
| **84** | *Lipotes vexillifer* AMNH 57333 | *Lipotes vexillifer* | Odontocete | Lipotidae | Extant |
| **85** | *Lissodelphis borealis* USNM 550188 | *Lissodelphis borealis* | Odontocete | Delphinidae | Extant |
| **86** | *Lissodelphis peronii* NMNZ MM002116 | *Lissodelphis peronii* | Odontocete | Delphinidae | Extant |
| **87** | *Lomacetus ginsburgi* MNHN.F.PPI.104 | *Lomacetus ginsburgi* | Odontocete | Phocoenidae | Miocene |
| **88** | *Megaptera novaeangliae* GERM.792a (NHMUK) | *Megaptera novaeangliae* | Mysticete | Balaenopteridae | Extant |
| **89** | *Mesoplodon bidens* USNM 593438 | *Mesoplodon bidens* | Odontocete | Ziphiidae | Extant |
| **90** | *Mesoplodon bowdoini* NMNZ MM001900 | *Mesoplodon bowdoini* | Odontocete | Ziphiidae | Extant |
| **91** | *Mesoplodon carlhubbsi* USNM 504128 | *Mesoplodon carlhubbsi* | Odontocete | Ziphiidae | Extant |
| **92** | *Mesoplodon europaeus* USNM 571665 | *Mesoplodon europaeus* | Odontocete | Ziphiidae | Extant |
| **93** | *Mesoplodon ginkgodens* USNM 298237 | *Mesoplodon ginkgodens* | Odontocete | Ziphiidae | Extant |
| **94** | *Mesoplodon grayi* USNM 49880 | *Mesoplodon grayi* | Odontocete | Ziphiidae | Extant |
| **95** | *Mesoplodon hectori* NHMUK 1949.8.19.1 | *Mesoplodon hectori* | Odontocete | Ziphiidae | Extant |
| **96** | *Mesoplodon hotaula* USNM 593426 | *Mesoplodon hotaula* | Odontocete | Ziphiidae | Extant |
| **97** | *Mesoplodon layardii* USNM 550150 | *Mesoplodon layardii* | Odontocete | Ziphiidae | Extant |
| **98** | *Mesoplodon mirus* USNM 504612 | *Mesoplodon mirus* | Odontocete | Ziphiidae | Extant |
| **99** | *Mesoplodon peruvianus* USNM 571258 | *Mesoplodon peruvianus* | Odontocete | Ziphiidae | Extant |
| **100** | *Mesoplodon stejnegeri* USNM 504330 | *Mesoplodon stejnegeri* | Odontocete | Ziphiidae | Extant |
| **101** | *Mesoplodon traversii (juvenile)* NMNZ TMP012996 | *Mesoplodon traversii (juvenile)* | Odontocete | Ziphiidae | Extant |
| **102** | *Messapicetus longirostris* MSNUP NN | *Messapicetus longirostris* | Odontocete | Ziphiidae | Miocene |
| **103** | *Miocaperea pulchra* SMNS 46978 | *Miocaperea pulchra* | Mysticete | Cetotheriidae | Miocene |
| **104** | *Mixocetus sp* LACM 143474 | *Mixocetus sp* | Mysticete | Tranatocetidae | Miocene |
| **105** | *Monodon monoceros* USNM 267959 | *Monodon monoceros* | Odontocete | Monodontidae | Extant |
| **106** | *Neophocaena asiaeorientalis* USNM 240001 | *Neophocaena asiaeorientalis* | Odontocete | Phocoenidae | Extant |
| **107** | *Neophocaena phocaenoides* NHMUK 1903.9.12.3 | *Neophocaena phocaenoides* | Odontocete | Phocoenidae | Extant |
| **108** | *Notocetus vanbenedeni* MLP 55 | *Notocetus vanbenedeni* | Odontocete | Squalodelphinidae | Miocene |
| **109** | *Odobenocetops* *peruvianus* SMNK PAL 2491 | *Odobenocetops peruvianus* | Odontocete | Odobenocetopsidae | Pliocene |
| **110** | *Orcaella brevirostris* NHMUK.1883.11.20.2 | *Orcaella brevirostris* | Odontocete | Delphinidae | Extant |
| **111** | *Orcaella heinsohni* USNM 284430 | *Orcaella heinsohni* | Odontocete | Delphinidae | Extant |
| **112** | *Orcinus orca* USNM 11980 | *Orcinus orca* | Odontocete | Delphinidae | Extant |
| **113** | *Orycterocetus crocodilinus* USNM 22926 | *Orycterocetus crocodilinus* | Odontocete | Physeteridae | Miocene |
| **114** | *Pakicetus attocki* PV M 100148 cast | *Pakicetus attocki* | Archaeocete | Pakicetidae | Eocene |
| **115** | *Papahu taitapu* OU 22066 | *Papahu taitapu* | Odontocete | Waipatiidae | Miocene |
| **116** | *Parapontoporia sternbergi* SDNHM 75060 | *Parapontoporia sternbergi* | Odontocete | Lipotidae | Pliocene |
| **117** | *Parietobalaena palmeri* USNM 24883 | *Parietobalaena palmeri* | Mysticete | Pelocetidae | Miocene |
| **118** | Patriocetid *sp* new genus ChM PV4753 | Patriocetid | Odontocete | Patriocetidae | Oligocene |
| **119** | *Patriocetus ehrlichii* 1999-3 Cet. 4 | *Patriocetus ehrlichii* | Odontocete | Patriocetidae | Oligocene |
| **120** | *Patriocetus sp* MB Ma. 42882 | *Patriocetus sp* | Odontocete | Patriocetidae | Oligocene |
| **121** | *Pelocetus calvertensis* USNM 11976 | *Pelocetus calvertensis* | Mysticete | Pelocetidae | Miocene |
| **122** | *Peponocephala electra* USNM 504511 | *Peponocephala electra* | Odontocete | Delphinidae | Extant |
| **123** | *Phocoena dioptrica* NHMUK 1939.9.30.1 | *Phocoena dioptrica* | Odontocete | Phocoenidae | Extant |
| **124** | *Phocoena phocoena* AMNH 212161 | *Phocoena phocoena* | Odontocete | Phocoenidae | Extant |
| **125** | *Phocoena sinus* SDNHM 20697 | *Phocoena sinus* | Odontocete | Phocoenidae | Extant |
| **126** | *Phocoena spinipinnis* NHMUK 1900.5.7.29 | *Phocoena spinipinnis* | Odontocete | Phocoenidae | Extant |
| **127** | *Phocoenoides dalli* USNM 276062 | *Phocoenoides dalli* | Odontocete | Phocoenidae | Extant |
| **128** | *Physeter macrocephalus* NHMUK 2007.1 | *Physeter macrocephalus* | Odontocete | Physeteridae | Extant |
| **129** | *Piscobalaena nana* MNHN 1618 cast | *Piscobalaena nana* | Mysticete | Cetotheriidae | Miocene |
| **130** | *Piscolithax longirostris* SAS 933 | *Piscolithax longirostris* | Odontocete | Phocoenidae | Miocene |
| **131** | *Platanista gangetica* USNM 172409 | *Platanista gangetica* | Odontocete | Platanistidae | Extant |
| **132** | *Pliopontos littoralis* SAS 193 | *Pliopontos littoralis* | Odontocete | Pontoporiidae | Pliocene |
| **133** | *Pontoporia blainvillei* USNM 482727 | *Pontoporia blainvillei* | Odontocete | Pontoporiidae | Extant |
| **134** | *Prosqualodon davidis* USNM 467596 | *Prosqualodon davidis* | Odontocete | Prosqualodontidae | Oligocene |
| **135** | *Protocetus atavus* SMNS 11084 | *Protocetus atavus* | Archaeocete | Protocetidae | Eocene |
| **136** | *Pseudorca crassidens* USNM 11320 | *Pseudorca crassidens* | Odontocete | Delphinidae | Extant |
| **137** | *Remingtonocetus harudiensis* USNM PAL 559313 | *Remingtonocetus harudiensis* | Archaeocete | Remingtonocetidae | Eocene |
| **138** | **Saiga tatarica* NHMUK 1961.5.30.1 | *Saiga tatarica* | Terrestrial artiodactyl | Bovidae | Extant |
| **139** | *Schizodelphis barnesi* MNHN AMN 19 | *Schizodelphis barnesi* | Odontocete | Eurhinodelphinidae | Miocene |
| **140** | *Schizodelphis morckhoviensis* USNM 13873 | *Schizodelphis morckhoviensis* | Odontocete | Eurhinodelphinidae | Miocene |
| **141** | *Schizodelphis sp* CCNHM 141 | *Schizodelphis sp* | Odontocete | Eurhinodelphinidae | Miocene |
| **142** | *Semirostrum cerutti* SDNHM 65276 | *Semirostrum cerutti* | Odontocete | Phocoenidae | Pliocene |
| **143** | *Septemtriocetus bosselaersi* IRSNB M.1928 | *Septemtriocetus bosselaersi* | Odontocete | Phocoenidae | Pliocene |
| **144** | *Shark tooth Squalodon* *sp* OU 21798 | *Squalodon sp* | Odontocete | Squalodontidae | Oligocene |
| **145** | *Simocetus rayi* USNM 256517 | *Simocetus rayi* | Odontocete | Simocetidae | Oligocene |
| **146** | *Sotalia guianensis* USNM 571558 | *Sotalia guianensis* | Odontocete | Delphinidae | Extant |
| **147** | *Sousa chinensis* NHMUK 1992.97 | *Sousa chinensis* | Odontocete | Delphinidae | Extant |
| **148** | *Sousa plumbea* USNM 550941 | *Sousa plumbea* | Odontocete | Delphinidae | Extant |
| **149** | *Sousa sahulensis* NHMUK 1992.92 | *Sousa sahulensis* | Odontocete | Delphinidae | Extant |
| **150** | *Sousa teuszii* NHMUK 1992.138 | *Sousa teuszii* | Odontocete | Delphinidae | Extant |
| **151** | *Squalodon bariensis* IRSNB 2372 | *Squalodon bariensis* | Odontocete | Squalodontidae | Miocene |
| **152** | *Squalodon calvertensis* NMNZ MM001996 | *Squalodon calvertensis* | Odontocete | Squalodontidae | Miocene |
| **153** | *Stenella attenuata* NHMUK 1966.11.18.5 | *Stenella attenuata* | Odontocete | Delphinidae | Extant |
| **154** | *Stenella longirostris* USNM 395270 | *Stenella longirostris* | Odontocete | Delphinidae | Extant |
| **155** | *Steno bredanensis* USNM 572789 | *Steno bredanensis* | Odontocete | Delphinidae | Extant |
| **156** | *Tagicetus joneti* IRSNB M. 1892 | *Tagicetus joneti* | Odontocete | Delphinida | Miocene |
| **157** | *Tasmacetus shepherdi* USNM 484878 | *Tasmacetus shepherdi* | Odontocete | Ziphiidae | Extant |
| **158** | **Tayassu pecari labiatus* NHMUK 47.4.6.8 | *Tayassu pecari labiatus* | Terrestrial artiodactyl | Tayassuidae | Extant |
| **159** | *Tiucetus rosae* MNHN.F. PPI261 | *Tiucetus rosae* | Mysticete | Cetotheriidae | Miocene |
| **160** | **Tragulus kanchil* NHMUK 9.1.5.850 | *Tragulus kanchil* | Terrestrial artiodactyl | Tragulidae | Extant |
| **161** | *Tursiops aduncus* NHMUK 1882.1.2.3 | *Tursiops aduncus* | Odontocete | Delphinidae | Extant |
| **162** | *Tursiops truncatus* SDNHM 23798 | *Tursiops truncatus* | Odontocete | Delphinidae | Extant |
| **163** | *Waipatia maerewhenua* OU 22095 | *Waipatia maerewhenua* | Odontocete | Waipatiidae | Oligocene |
| **164** | *Xenorophus* *sp* ChM PV4823 | *Xenorophus sp* | Odontocete | Xenorophidae | Oligocene |
| **165** | *Xenorophus sp* Yap CCNHM 168 | *Xenorophus sp* | Odontocete | Xenorophidae | Oligocene |
| **166** | *Xiphiacetus bossi* USNM 8842 | *Xiphiacetus bossi* | Odontocete | Eurhinodelphinidae | Miocene |
| **167** | *Xiphiacetus cristatus* USNM 21363 | *Xiphiacetus cristatus* | Odontocete | Eurhinodelphinidae | Miocene |
| **168** | *Zarhachis flagellator* USNM 10911 | *Zarhachis flagellator* | Odontocete | Platanistidae | Miocene |
| **169** | *Zarhinocetus donnamatsonae* UCMP 86139 | *Zarhinocetus* | Odontocete | Allodelphinidae | Miocene |
| **170** | *Zarhinocetus errabundus* LACM 149588 | *Zarhinocetus* | Odontocete | Allodelphinidae | Miocene |
| **171** | *Ziphius cavirostris* NHMUK 2006.15 | *Ziphius cavirostris* | Odontocete | Ziphiidae | Extant |
| **172** | *Zygorhiza kochii* USNM 11962 | *Zygorhiza kochii* | Archaeocete | Basilosauridae | Eocene |

Table S10. Skulls scanned but excluded from analysis. Specimens were excluded for being too distorted, too heavily reconstructed or because of the unsure placement of them in the phylogeny*.

| Species | Specimen number |
| --- | --- |
| *Cephalotropis coronatus*  *Cynthiacetus peruvianus*  *Delphinodon dividum*  *Dilophodelphis fordycei*  *Eschrichtius robustus*  Patriocetid or Waipatiid***  *Pomatodelphis sp*  Waipatiid new gen  *Xenorophus* *sp** | USNM 489194   MNHN.F.PRU10   USNM 7278   USNM 214911   USNM 13803  CCNHM 1078  USNM 187414  ChM PV7679  ChM PV7677 |


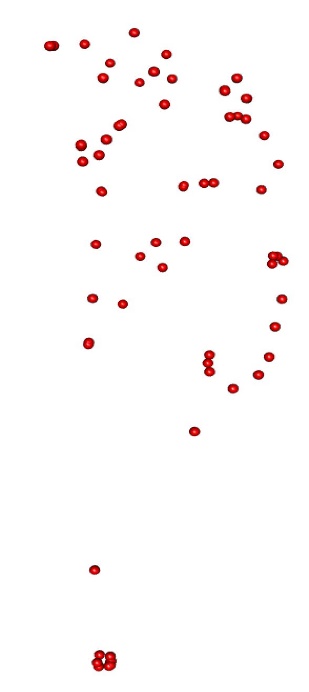

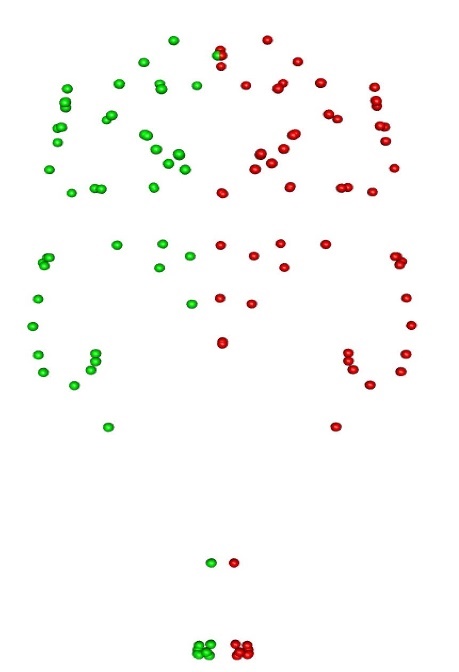

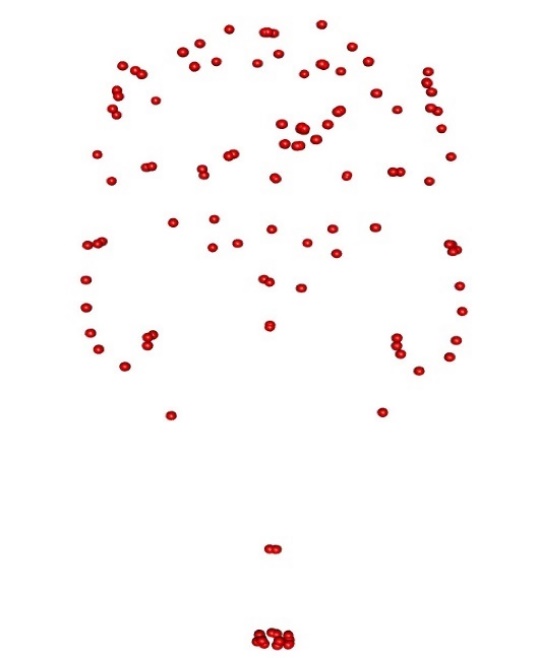


**a. b. c.**

Fig S15. The landmark configuration with (a) manually placed landmarks on half of the skull to be mirrored to the other half of the skull, (b) the inaccurate computer mirrored landmarks, and (c) the correct manually placed landmarks. Note the differences between the positioning of landmarks b. and c. Shown on the skull of *Delphinapterus leucas* USNM 305071.

[next page]

**Landmarks used in the study**

Table S11. 123 landmarks added to the entire surface of the skull. Landmarks highlighted in red were placed on the midline. Landmarks 1-66 (with 38, 40, 48, 49, 51, 54, 55, 56, 61 on the midline) were placed on the LHS, landmarks 67-123 were placed on the RHS.

| **Landmark description** | **Number on LHS of the skull** | **Number on RHS of the skull** |
| --- | --- | --- |
| Nasal anterior | 1 | 120 |
| Left anterior lateral nasal | 2 | 67 |
| Posterior lateral corner of nasal | 3 | 68 |
| Posterior point of nasal | 4 | 121 |
| Tip of rostrum, anterior dorsal side, anterior midline of tooth row (usually premaxilla) | 5 | 69 |
| Anterior dorsal premaxilla | 6 | 70 |
| Posterior dorsal premaxilla | 7 | 71 |
| Anterior lateral ventral premaxilla | 8 | 72 |
| Anterior lateral ventral maxilla | 9 | 73 |
| Dorsal medial maxilla (suture with nasal and premaxilla) | 10 | 74 |
| Nasal-frontal-maxilla suture (posterior medial maxilla) | 11 | 75 |
| Dorsal posterior maxilla on orbit (including lacrimal - dorsal suture frontal) on orbit | 12 | 76 |
| Jugal maxilla orbit suture - front orbit lateral | 13 | 77 |
| Posterior ventral lateral most point of maxilla – tooth row - Jugal-maxilla ventral suture | 14 | 78 |
| Posterior tooth row lateral maxilla or lateral maxilla in species with no/negligible dentition | 15 | 79 |
| Jugal anterior dorsal | 16 | 80 |
| Jugal anterior ventral | 17 | 81 |
| Jugal posterior ventral | 18 | 82 |
| Anterior medial frontal | 19 | 122 |
| Posterior medial frontal | 20 | 123 |
| Lateral posterior frontal (posterior lateral parietal suture) | 21 | 83 |
| Postorbital process/bar tip (anterior on crest) | 22 | 84 |
| Anterior lateral frontal (on orbit) | 23 | 85 |
| Anterior dorsal corner of frontal (on orbit) | 24 | 86 |
| Anterior medial parietal | 25 | 87 |
| Posterior medial parietal | 26 | 88 |
| Posterior lateral parietal (squamosal/occipital suture) | 27 | 89 |
| Anterior lateral parietal (on vault) | 28 | 90 |
| Dorsal anterior lateral parietal (suture with frontal) | 29 | 91 |
| Dorsal anterior squamosal suture (with parietal, maybe alisphenoid/frontal) | 30 | 92 |
| Medial anterior zygomatic vault junction (squamosal) | 31 | 93 |
| Anterior dorsal jugal-squamosal suture | 32 | 94 |
| Posterior ventral jugal-squamosal suture, lateral | 33 | 95 |
| Anterior medial most point of the mandibular articular process | 34 | 96 |
| Posterior lateral most point of the mandibular articular process | 35 | 97 |
| Lateral posterior squamosal (occipital suture) | 36 | 98 |
| Posterior medial dorsal squamosal (parietal/occipital suture) | 37 | 99 |
| **MIDLINE:** posterior margin of skull roof | *38* | *38* |
| Medial anterior supraoccipital (parietal-occipital suture, usually) | 39 | 100 |
| **MIDLINE:** dorsal/superior margin of foramen magnum | *40* | *40* |
| Dorsal medial occipital condyle | 41 | 101 |
| Dorsal lateral occipital condyle | 42 | 102 |
| Tip of paraoccipital process - lateral tip | 43 | 103 |
| Lateral ventral occipital + process | 44 | 104 |
| Lateral dorsal occipital | 45 | 105 |
| Ventral medial occipital condyle | 46 | 106 |
| Ventral lateral occipital condyle | 47 | 107 |
| **MIDLINE:** ventral margin of foramen magnum | *48* | *48* |
| **MIDLINE:** anterior basioccipital | *49* | *49* |
| Lateral anterior basioccipital | 50 | 108 |
| **MIDLINE:** anterior most point of basisphenoid, just posterior to the pterygoids and palate | *51* | *51* |
| Lateral anterior basisphenoid | 52 | 109 |
| Lateral posterior basisphenoid | 53 | 110 |
| **MIDLINE:** Medial posterior basisphenoid | *54* | *54* |
| **MIDLINE:** Posterior ventral medial point of palate | *55* | *55* |
| **MIDLINE:** Palatine anterior midline ventral suture | *56* | *56* |
| Pal-pterygoid suture | 57 | 111 |
| Pal-max lateral posterior suture | 58 | 112 |
| Pterygoid posterior | 59 | 113 |
| Ventral posterior pterygoid | 60 | 114 |
| **MIDLINE:** Maxilla ventral midline posterior suture | *61* | *61* |
| Maxilla ventral midline anterior suture | 62 | 115 |
| Maxilla anterior lateral ventral | 63 | 116 |
| Premaxilla ventral midline posterior suture | 64 | 117 |
| **MIDLINE:** Anterior-most point of palatal surface immediately posterior to tooth row | 65 | 118 |
| Premaxilla posterior lateral ventral | 66 | 119 |


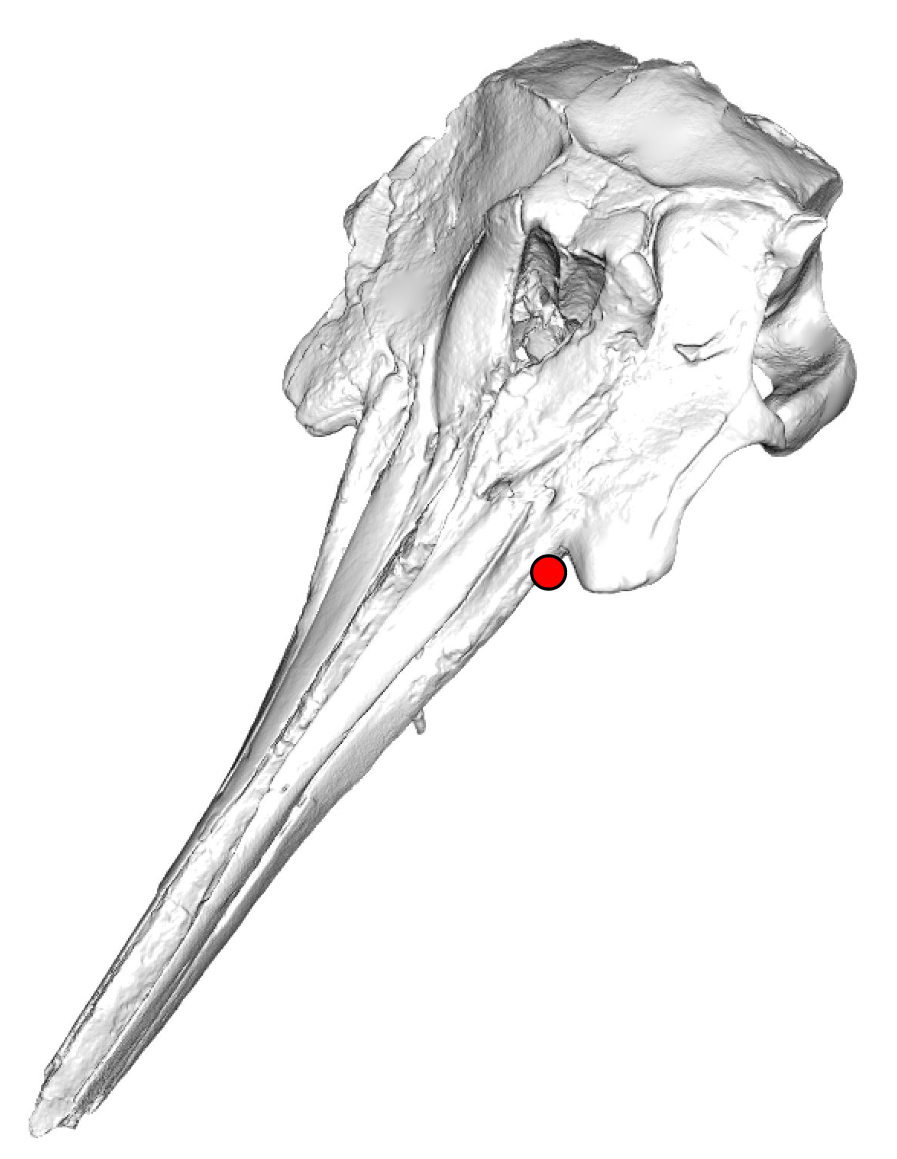

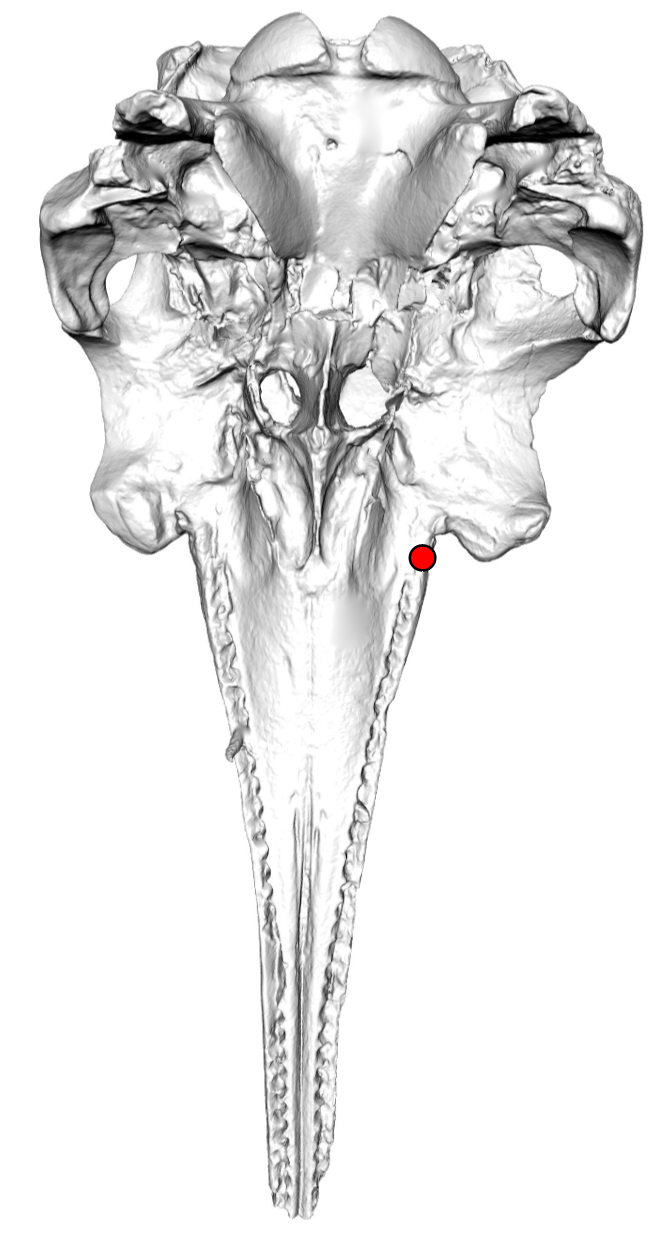


**a. b.**

Fig S16. The position of landmark 15 (to be mirrored as landmark 79): Posterior tooth row lateral maxilla or lateral maxilla in specimens with no/negligible dentition. This positioning is used for all specimens including those that lack a true tooth row with mandibular prognathism, absent, maxillary-only, or vestigial dentition (including Ziphiids, narwhals (*Monodon monoceros*) and sperm whales (*Physeter macrocephalus*)). Landmarks are shown on *Kentriodon pernix* (USNM 10670) (which has a visible tooth row) in an oblique (a) and ventral (b) view. Not to scale.

Table S12. Likelihood model results (AIC) for each potential scenario for asymmetry in the cetacean cranium. We used the ‘fitDiscrete’ function in ‘geiger’ v.1.3-1 [114] to fit various likelihood models for discrete character evolution. These model arguments were an ‘equal-rates’ model (ER) where all transitions occur at equal rates, a ‘symmetric transitions are equal’ model (SYM), and ‘all rates different model’ (ARD) where each rate is a separate parameter [117].

| **Model** | **Likelihood model** | **AIC** | **AICc** | **Rank** |
| --- | --- | --- | --- | --- |
| Ancestral  Ancestral  Ancestral  Regime  Regime  Regime  Regime split  Regime split  Regime split  Echolocation  Echolocation  Echolocation  Echolocation frequency  Echolocation frequency  Echolocation frequency | “ER”  “ARD”  “SYM”  “ER”  “SYM”  “ARD”  “ER”  “SYM”  “ARD”  “ER”  “SYM”  “ARD”  “ER”  “SYM”  “ARD” | 32.844513  33.656537  34.937848  64.520625  68.892622  73.136210  69.724158  93.108987  115.719725  52.666484  56.887372  58.250569  241.957102  247.316761  279.003293 | 32.869513  34.198473  35.089747  64.545625  69.434558  75.230170  69.749158  96.396659  129.918198  52.691484  57.429308  60.344528  241.982102  253.916761  309.356235 | 1  2  3  1  2  3  1  2  3  1  2  3  1  2  3 |
